# Unifying Scaling Relations and Multiple Reaction Mechanisms for Screening Transition Metal‐Doped Co_3_O_4_ for Oxygen Evolution Reaction

**DOI:** 10.1002/anie.202524523

**Published:** 2026-05-04

**Authors:** Kapil Dhaka, Hatem M. A. Amin, Davide Beschi, Dana Schellenburg, Benjamin Mockenhaupt, Stephan Barcikowski, Stephan Schulz, Kai S. Exner

**Affiliations:** ^1^ University of Duisburg‐Essen, Faculty of Chemistry, Theoretical Catalysis and Electrochemistry Universitätsstraße 5 Essen Germany; ^2^ University of Duisburg‐Essen, Faculty of Chemistry, Institute of Inorganic Chemistry Universitätsstraße 5 Essen Germany; ^3^ University of Duisburg‐Essen, Faculty of Chemistry, Technical Chemistry I Universitätsstraße 5 Essen Germany; ^4^ Center For Nanointegration (CENIDE) Duisburg‐Essen Duisburg Germany; ^5^ Cluster of Excellence RESOLV Bochum Germany

**Keywords:** Co_3_O_4_, density functional theory, descriptor approach, nanoparticle catalyst, oxygen evolution reaction, reaction mechanism

## Abstract

Accelerating the discovery of oxygen‐evolution reaction (OER) catalysts requires high‐throughput screening strategies combining descriptor‐based frameworks with dedicated mechanistic analyses. In this study, we present a unified methodology using the example of doped Co_3_O_4_ in the OER by developing a mechanistically resolved, potential‐dependent volcano approach that accounts for the uncertainty of adsorption free energies when analyzing activity trends. We evaluate the influence of different dopants (Cr, Mn, Fe, Ni, Cu, and V) on the OER activity by selectively substituting octahedral Co sites on the (001) facet of Co_3_O_4_ using density functional theory calculations (DFT). We identify Cr, Fe, Ni, and V as promising dopants as they exhibit increased OER activity compared to undoped Co_3_O_4_, while Cr shows the strongest promoting effect among all dopants considered in this study. We compare our theoretical predictions with two different series of synthesized Co_3_O_4_ nanoparticle catalysts and find good agreement regarding the qualitative trends of OER activity. To validate the strong promoting effect of Cr, we synthesize surface‐enriched, Cr‐doped Co_3_O_4_ nanoparticles, which confirms the theoretical prediction of increased OER activity. The theoretical model developed in this work is a transferable framework that can be equally applied to other materials and electrocatalytic processes for quantifying dopant effects by considering uncertainty and promoting effects when analyzing activity trends.

## Graphical abstract

Doped Co_3_O_4_ climbs a mechanistic volcano: transition‐metal substitution reshapes the OER activity landscape, while multiple competing reaction mechanisms govern the volcano trends across different dopants.

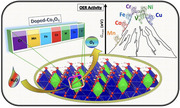

## 1 Introduction

Rapid identification of efficient electrocatalysts for oxygen evolution reaction (OER) — 2 H_2_O → O_2_ + 4 H^+^ + 4 e^−^, *U* ^0^_OER_ = 1.23 V vs. RHE (reversible hydrogen electrode)—calls for screening strategies that traverse large compositional spaces while preserving mechanistic fidelity [1]. Descriptor‐based frameworks and volcano plots have emerged as powerful tools for high‐throughput screening of OER catalysts using adsorption free energies [2, 3]. It is a unifying consensus that the adsorption free energies of the key OER intermediates, *OH, *O, and *OOH, are intrinsically coupled [4, 5]. These correlations manifest as linear scaling relationships, typically expressed as ΔG_*O_ vs. ΔG_*OH_ and ΔG_*OOH_ vs. ΔG_*OH_, which reduce dimensionality but also impose intrinsic constraints on catalyst optimization [6]. As a result, activity trends are often summarized by relying on the ΔG_*OOH_ vs. ΔG_*OH_ relation in the form of a volcano plot that projects materials’ performance onto a single binding‐energy axis [5, 7, 8]. This approach has gained cult status in the community based on the ease of testing catalytic materials in silico using thermodynamic considerations [9].

Most work in the OER literature uses the mononuclear pathway [10], which consists of the sequential formation of the *OH, *O, and *OOH adsorbates, to describe the proton‐coupled electron transfer steps culminating in the formation of gaseous oxygen at a single active site [5, 10]. Real oxide surfaces, especially doped ones, can access dual‐site and multi‐site routes [11, 12]. Dopants may act as the active centers or as promoters that modulate the electrocatalytic activity of active sites through electronic and geometric effects [13]. Such cooperative behavior enables bifunctional, binuclear, Walden‐type, and oxide‐mediated mechanisms, which can compete with or surpass the mononuclear description depending on the local environment [14, 15, 16]. However, screening frameworks that allow for the inclusion of mechanistic diversity while relying on effective descriptors using the concept of adsorption free energies are lacking in the literature.

Substitutional doping is a particularly effective lever to enhance the electrocatalytic activity: introducing 3d transition metals (TM) into a robust oxide host can tune electronic structure, modulate metal–oxygen covalency, reshape adsorption energetics, and stabilize reactive surface states. Several works in the literature have addressed doping effects for spinel cobalt oxide (Co_3_O_4_) for OER [17, 18, 19, 20, 21, 22, 23, 24, 25, 26, 27, 28, 29, 30], although detailed atomic‐level insights explaining the targeted doping are lacking. This requires theoretical considerations analyzed with volcano‐based approaches that go beyond the mononuclear pathway and a single active site to carefully scan the vast parameter space of doped Co_3_O_4_ structures accessible to OER. On the other hand, electrocatalysts identified based on computational high‐throughput screening often cannot be directly compared with experimentally synthesized catalysts due to the obvious length gap between atomic‐scale models and nanoscale catalytic materials.

In the present work, we close the outlined knowledge gaps by introducing a universal methodology for TM‐doped Co_3_O_4_ (dopants: Cr, Mn, Fe, Ni, Cu, V) to solve the impact of dopants on OER activity by analyzing promoting effects. In addition to a variety of different mechanistic descriptions, the scaling relationship ΔG_*O_ vs. ΔG_*OH_, a potential‐dependent activity assessment using the *G*_max_(*U*) descriptor [31, 32], and multiple active‐site configurations are considered in the analysis of adsorption free energies for OER. Our theoretical framework is then coupled with different series of doped Co_3_O_4_ catalysts, which are experimentally tested for OER activity. While our calculations suggest that Cr as a dopant at the Co_3_O_4_ surface has the main promoting effect for OER, we validate our theoretical prediction by the synthesis and experimental characterization of a surface‐enriched, Cr‐doped Co_3_O_4_ nanocube using linear sweep voltammetry [33, 34, 35, 36, 37].

## 2 Theoretical Model

All calculations were carried out within the framework of spin‐polarized density functional theory (DFT) using the projector‐augmented‐wave formalism and the PBE exchange‐correlation functional. On‐site Coulomb interactions were treated with PBE+U for Co and the 3d transition metal dopants, with element‐specific U values listed in the Supporting Information. All further computational details are provided in Section S1.

### 2.1 Surface Models and Doping Schemes

**FIGURE 1:**
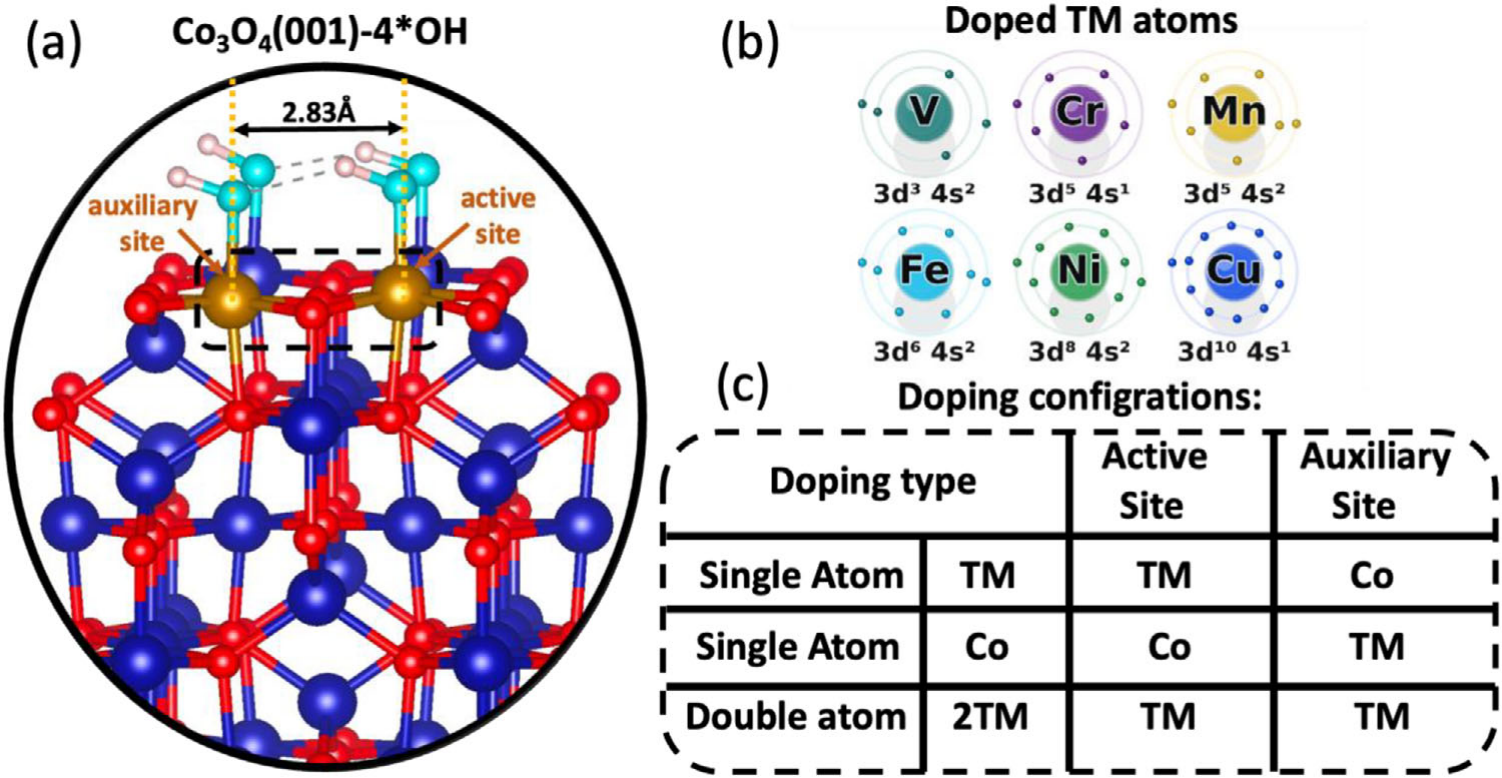
Schematic illustration of transition‐metal (TM) doping for the thermodynamically stable Co_3_O_4_ (001)‐4*OH surface. (a) Representative surface model highlighting the Co_3_O_4_(001) termination, with selected TM dopants (yellow) incorporated into surface Co sites. The structural motif shows the local environment of dopants and the TM‐TM spacing (≅2.83 Å) relevant for catalytic activity. The auxiliary and active sites correspond to octahedrally coordinated cobalt sites (Co_oct_). (b) Electronic configurations of the considered TM 3d dopants (V, Cr, Mn, Fe, Ni, Cu), depicted as atomic orbitals with valence electron distributions (3d^n^,4s^m^). (c) Dopant configurations are summarized as single‐atom doping (TM and Co are located at either the active or auxiliary sites) and double‐atom doping (2TM refers to TM at the active site and auxiliary site both).

The spinel structure of Co_3_O_4_ was adopted with a 2 × 2 (001) termination‐B slab exposing four surface octahedral Co_oct_ cations (cf. Figure S1), which corresponds to the dominant exposed facet in nanostructured Co_3_O_4_ catalysts. Under OER conditions, the undercoordinated Co_oct_ surface sites are fully capped by *OH adsorbates, as evident from the construction of ab initio Pourbaix diagrams in previous works [16, 18, 38]. Therefore, we select the 2 × 2 Co_3_O_4_(001)‐4*OH configuration (cf. Figure 1a) as a starting point for the investigation of dopant effects in the OER. Note that the use of Co_oct_ surface sites as the active site for OER is consistent with experimental and theoretical studies on this topic [39, 40, 41, 42], although it should be emphasized that neighboring Co_oct_ sites can serve as auxiliary sites (cf. Figure 1a) during the elementary steps by acting as Brønsted acid or base [38]. By considering neighboring sites, we extend conventional models based on a single active site to an ensemble of different configurations for investigating the elementary steps for OER.

Substitutional doping is introduced by replacing Co_oct_ surface sites with a 3d transition metal (TM), namely V, Cr, Mn, Fe, Ni, or Cu (cf. Figure 1b). Doping is restricted to the outer octahedral layer that hosts OER intermediates; subsurface tetrahedral cations remain Co. The surface contains four exposed Co sites per cell, and we replace either one or two of them, which corresponds to a surface dopant fraction of 25% (1/4) or 50% (2/4), respectively. Across the entire slab, there are 28 Co atoms in total, so that the total dopant concentration is 3.6% (1/28) or 7.1% (2/28), which is on the order of magnitude of experimental studies on doped Co_3_O_4_ [27, 28, 43, 44, 45, 46, 47]. All doped slabs are fully relaxed while retaining the same 4*OH arrangement to isolate the effect of cation substitution from changes in surface coverage.

Three configurations are considered to separate intrinsic activity at the binding site from promotional effects of a neighbor (cf. Figure 1c):

- Active site TM, Auxiliary site Co (label “**TM**”): the active site is replaced by a dopant (cf. Figure 1b), and the auxiliary site remains Co.
- Active site Co, Auxiliary site TM (label “**Co**”): the active site remains Co, and the auxiliary site is replaced by a dopant.
- Active site TM, Auxiliary site TM (label “**2TM**”): both sites are replaced by the same dopant.

### 2.2 Computational Workflow and Analysis Framework

Although the catalytic activity of electrocatalytic processes is determined by the kinetics (transition states) in the free‐energy diagram along the reaction coordinate, there is consensus in the theoretical electrocatalysis community to rely on the determination of Gibbs free‐energy changes (Δ*G*) for the elementary reaction steps of OER [4, 5, 10]. The main reason for this approach is that the calculation of transition states for proton‐coupled electron transfer steps is computationally intensive and associated with significant errors [48, 49], making this strategy unsuitable for heuristic materials screening [50]. To this end, we apply the computational hydrogen electrode (CHE) approach [51] to determine potential‐dependent Δ*G*(*U*) values for seven different OER mechanisms, which are summarized in Section S2. Further computational details on the determination of Δ*G* values by incorporating zero‐point and entropy corrections for adsorbates and gas molecules, implicit solvation [52], and the application of gas‐phase error corrections [53, 54] to overcome the DFT bias leading to incorrect prediction of reaction energies are given in Section S1. The potential‐dependent Δ*G*(*U*) values for the different OER pathways are used to determine the electrocatalytic activity using the *G*_max_(*U*) descriptor [31, 32], which links thermodynamics to kinetics through a free‐energy span model using the Brønsted‐Evans‐Polanyi relationship [55]. For a given reaction mechanism, the descriptor *G*_max_(*U*) is defined as

(1) $$\begin{array}{ccc} G_{\max}\left(U\right) & = & \mathrm{ma}x_{\left(1\leq p<q\leq N+1\right)}\left[G_{q}\left(U\right)-G_{p}\left(U\right)\right]\\ & = & \mathrm{ma}x_{\left(1\leq p<q\leq N+1\right)}\sum_{k=p}^{q-1}\Delta G_{k}\left(U\right). \end{array}$$

In Equation (1), *N* corresponds to the number of elementary steps (states indexed as 1, …, *N*+1); *p* and *q* are indices of two intermediate states (with the respective Gibbs free energy, *G*), which need to adhere to the condition *p* < *q*; *k* is a running index that ensures that all free‐energy spans between the intermediate states are systematically evaluated. Typically, *N* = 4 for the four proton‐coupled electron transfer steps in OER (thus, *p*, *q* ∈ {1, …, 5}). In case certain mechanistic pathways include a chemical step in the description (cf. Section S2), then *N* = 5 and *p*, *q* ∈ {1, …, 6}.

Based on the *G*_max_(*U*) values for the different mechanistic pathways, we can extract the energetically preferred description by referring to the lowest values; this descriptor is shown on the *y*‐axis of a volcano plot. In addition, the free‐energy change between the *O and *OH intermediates is used as a descriptor on the *x*‐axis of a volcano plot based on previous work on the topic, although these works omitted the potential dependence of the OER volcano curve [1, 8, 56, 57], which is considered here. The trend line in the volcano plot is determined by a dedicated evaluation of the scaling relationships ΔG_*O_ vs. ΔG_*OH_ and ΔG_*OOH_ vs. ΔG_*OH_, as further described in Section S4.

## 3 Results and Discussion

### 3.1 OER Mechanisms for Doped Co_3_O_4_(001) Systems

**FIGURE 2:**
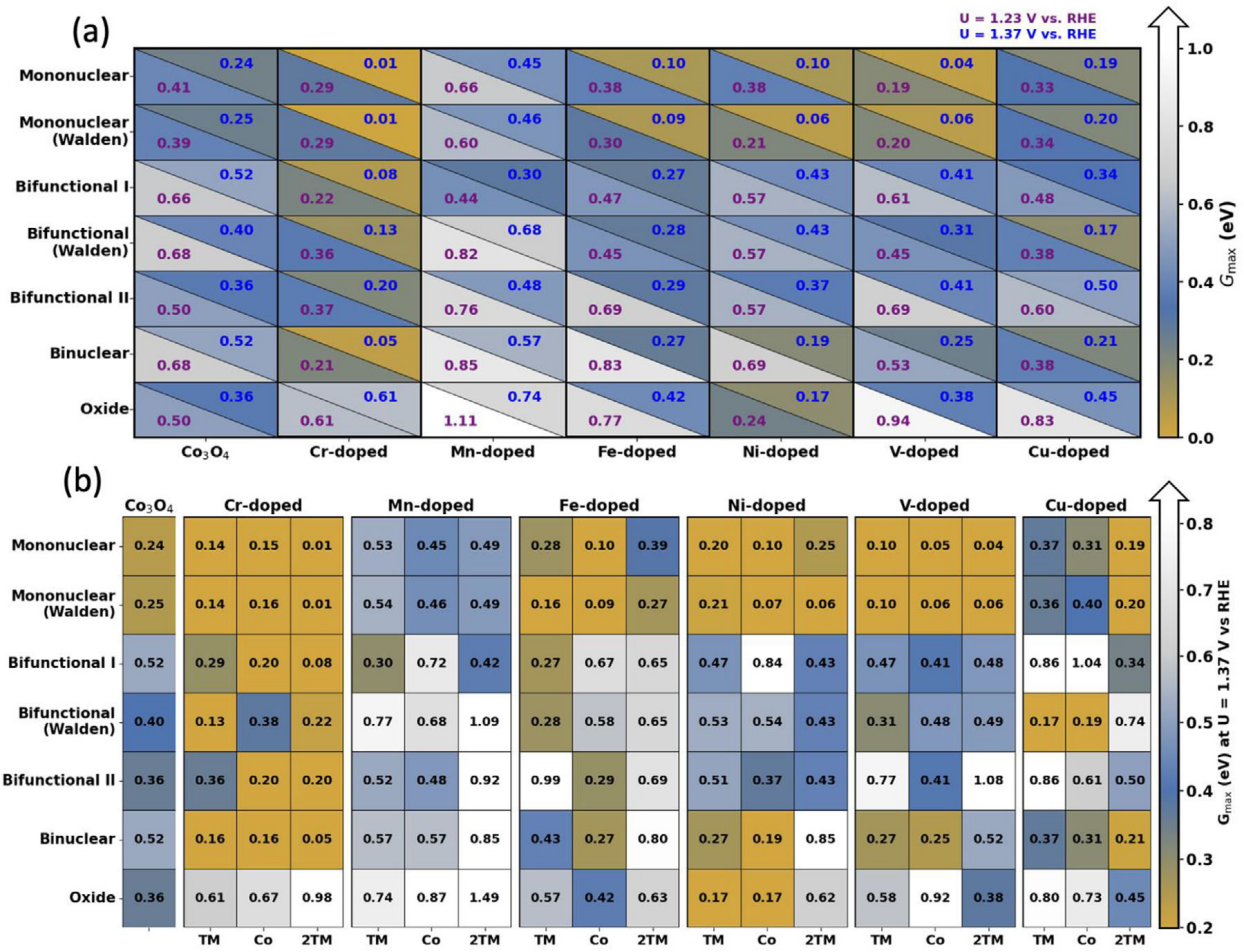
(a) Heatmap of *G*_max_(*U*) for Co_3_O_4_(001) and TM‐doped variants (cf. Figure 1) across seven mechanisms. Rows list mechanisms; columns list catalyst systems. Each cell reports the lowest *G*_max_(*U*) among the different dopant configurations (TM/Co/2TM); purple and blue numbers correspond to *U* = 1.23 V and 1.37 V vs. RHE, respectively. The color bar indicates *G*_max_(*U*) in eV. (b) Heatmap of *G*_max_(*U*) at *U* = 1.37 V vs. RHE by differentiating between the different dopant configurations (TM/Co/2TM) in dependence on the mechanistic pathway. Note that a lower *G*_max_(*U*) value corresponds to higher OER activity.

We investigate seven OER pathways on the pristine and TM‐doped Co_3_O_4_(001)‐4*OH surface using the active‐site motifs in Figure 1. Free‐energy diagrams at *U* = 1.37 V vs. RHE are provided in Section S3 (cf. Figures S3–S8). We choose *U* = 1.37 V vs. RHE (*η*_OER_ = 0.14 V) as the target electrode potential for the energetic analysis to keep the free‐energy spans (cf. equation (1)) meaningful and to exclude cases where *G*_max_(*U*) is negative. Figure 2a compiles these results by extracting for each composition and site motif the smallest *G*_max_(*U*) value among the seven OER pathways at *U* = 1.23 and 1.37 V vs. RHE. On the other hand, Figure 2b shows how the dopant placement (cf. Figure 1c) controls the electrocatalytic activity at *U* = 1.37 V vs. RHE.

Across compositions, Walden‐type mechanisms [50] often exhibit the smallest values of the *G*_max_(*U*) descriptor. However, these pathways are not captured within traditional screening frameworks, which typically rely on adsorption energetics derived from the classical mononuclear adsorbate‐evolution mechanism (*OH → *O → *OOH → O_2_) and its associated scaling relations. In this context, the conventional mononuclear mechanism serves as a proxy for direct substitution of the active metal site (TM), reflecting systematic tuning of metal–adsorbate bond strengths.

In contrast, Walden‐type pathways involve a concerted step in which O_2_ evolution and water adsorption occur simultaneously, such that a formally vacant metal site is never restored. Although a mononuclear‐Walden mechanism still operates on a single metal center, its energetics cannot be inferred from classical mononuclear adsorption descriptors alone. This explains why Walden‐type pathways may become favorable even when the traditional mononuclear mechanism is not.

For auxiliary‐site doping (Co) or dual doping (2TM), the classical mononuclear pathway is often not preferred. Several systems achieve equal or lower spans when the dopant is located at a neighboring auxiliary site (Co), suggesting a promoting effect via local hydrogen‐bond rearrangements and facilitated proton removal in bifunctional‐like descriptions. The dual‐dopant configuration (2TM) does not systematically enhance catalytic activity; in some cases, it matches the optimal single‐site configuration, while in others, it deteriorates the free‐energy span due to over‐stabilization of intermediates.

In summary, the observed trends point to a simple design rule based on Figure 2: optimal performance is most often achieved by a single, judiciously placed dopant that either directly tunes the binding site or promotes it from the nearest neighbor, while the use of dual‐dopant motifs (2TM) is generally not a great advantage given the present hydroxyl coverage of Co_3_O_4_ during OER.

Knowledge of the electrocatalytic activity based on the *G*_max_(*U*) descriptor allows us to compare the effect of each dopant on the OER performance. While we provide a detailed analysis in Section S3 (cf. Figure S9), we summarize here that Cr, Fe, Ni, V, and Cu as dopants enhance OER activity, while Mn‐doped Co_3_O_4_ leads to a decrease in OER activity. A comparison of this result with experimental works on the topic is provided in Sections 3.4–3.6.

### 3.2 Promoting Effect of Dopants on OER Activity

Typically, dopants are added to a material in low concentrations (less than 10%) to improve its catalytic properties. Considering that the majority of active sites retain the chemical nature of the original material, dopants can have two types of influence on catalytic activity: on the one hand, the dopants change the electronic structure of the active center due to their proximity, which is consistent with the case of auxiliary site doping (Co; cf. Figure 1c). On the other hand, the dopants can serve as an active center and form a minority species of active sites with a significantly higher electrocatalytic activity compared to the main active site. This scenario relates to the case of active site doping (TM; cf. Figure 1c). With increasing dopant concentration, the dopants can occupy both the active site and auxiliary site (2TM; cf. Figure 1c), which could further promote the activity of the electrocatalyst.

Based on the activity analysis in Figure 2, we evaluate the impact of the promoting effects of the selected dopants on the OER activity of Co_3_O_4_(001). This is achieved by quantifying Δ*G*_max_(*U*), which is a measure of the change in electrocatalytic activity upon TM doping. This descriptor is defined as

(2) $$\Delta G_{max}^{A\to B}\;\left(U\right)=G_{\max}^{B}\left(U\right)-\;G_{\max}^{A}\left(U\right).$$

In Equation (2), the different states A and B of the *G*_max_(*U*) descriptor refer to undoped‐Co_3_O_4_, Co, TM, or 2TM (cf. Figure 1c). We inspect the transitions of undoped‐Co_3_O_4_→Co, undoped‐Co_3_O_4_→TM, Co→TM, and TM→2TM and quantify the corresponding change in electrocatalytic activity. Note that for each state, we use the $G_{\max}^{X}\left(U\right)$ value, which is the minimum over all reaction mechanisms for the Co_3_O_4_(001)–4*OH model evaluated at *U* = 1.37 V vs. RHE (cf. Figure 2). A negative $\Delta G_{max}^{A\to B}\left(U\right)$ transition indicates an improvement of the OER activity in the transition from A to B, which is related to a promoting effect of the dopant.

**FIGURE 3:**
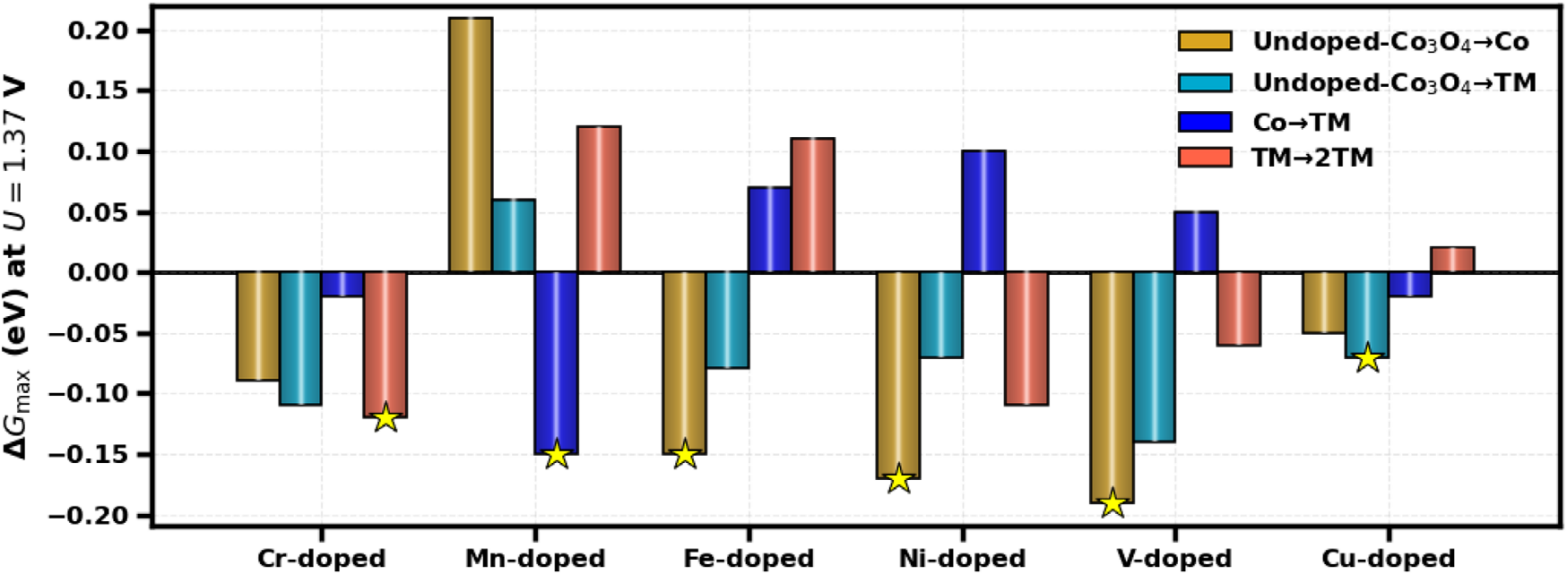
Change in the limiting free‐energy span (Δ*G*_max_(*U*), cf. Equation 2) at *U* = 1.37 V vs. RHE for undoped and different TM‐doped Co_3_O_4_ systems (cf. Figure 1c). Bars represent the effect of different doping configurations: Undoped‐Co_3_O_4_→Co (gold), Undoped‐Co_3_O_4_→TM (turquoise), Co→TM (blue), and TM→2TM (orange). Negative values correspond to a reduction in *G*_max_(*U*), which indicates a promoting effect of the respective dopant. Stars highlight the most favorable site configurations for each dopant.

Figure 3 illustrates that for Fe, Ni, and V doping, the undoped‐Co_3_O_4_→Co transition is negative and corresponds to the minimum value of Δ*G*_max_(*U*), indicating that these dopants are mainly suited as auxiliary promoters adjacent to a Co active site. These dopants also increase the OER activity when serving as the active site (Δ*G*_max_(*U*) < 0 for undoped‐Co_3_O_4_→TM, although the effect is less pronounced compared to the case of the auxiliary site, assisted by a positive Co→TM transition. Increasing the local dopant concentration by inspecting the TM→2TM transition has little effect for both Ni and V, while it is expected to have a detrimental effect on Fe doping due to a positive Δ*G*_max_(*U*) value. Therefore, we conclude that Fe, Ni, and V should be added mainly in small concentrations to the Co_3_O_4_ catalyst to activate active octahedral Co sites through electronic effects.

Mn doping is not preferential for Co_3_O_4_ in the OER, considering that the undoped‐Co_3_O_4_→Co and undoped‐Co_3_O_4_→TM transitions are positive. We note that the activity of Mn‐doped Co_3_O_4_ increases if Mn switches from the auxiliary to the active site (Δ*G*_max_(*U*) < 0 for Co→TM, although we emphasize that the resulting electrocatalytic activity is still lower than that of undoped Co_3_O_4_ (cf. Figure S9 in Section S3).

Cu doping has a small effect on the OER activity of Co_3_O_4_, considering that the Δ*G*_max_(*U*) values for the undoped‐Co_3_O_4_→Co and undoped‐Co_3_O_4_→TM transitions are close to zero. Despite this, the Cu‐doped Co_3_O_4_ is the only catalyst where the dopant as the active site corresponds to the preferential configuration for improved OER activity. Considering that at typical dopant concentrations of less than 10% there are not many active Cu sites on the Co_3_O_4_ surface, we conclude that Cu doping has a smaller positive effect on the OER activity compared to the dopants Fe, Ni, and V.

Finally, we recognize the strong promoting effect of Cr on the OER activity of Co_3_O_4_, which is qualitatively comparable to previous experimental studies in the literature [33, 34, 35, 36]. Notably, all four Δ*G*_max_(*U*) values are smaller than zero, indicating that Cr acts as a promoter of OER activity at the auxiliary site, at the active site, and at higher dopant concentrations in the 2TM state. This finding suggests enrichment of the Co_3_O_4_ surface with Cr sites to enhance OER activity. Our theoretical prediction is experimentally verified below (cf. section 3.6).

### 3.3 Scaling Relations and Volcano Plot for Doped Co_3_O_4_(001) Systems

OER activity is inherently limited due to scaling relations, and the main reason for the low activity of electrocatalysts under anodic polarization was attributed to the scaling relationship ΔG_*OOH_ vs. ΔG_*OH_ in previous work [5]. This well‐accepted paradigm was challenged in a recent contribution by Sokolov and Exner [7], pointing out that the OER volcano plot is more sensitive to the scaling relationship ΔG_*O_ vs. ΔG_*OH_ than to ΔG_*OOH_ vs. ΔG_*OH_. For this purpose, it is imperative to investigate the scaling relationships between all OER adsorbates (that is, *OH, *O, and *OOH) and use these scaling correlations to derive potential‐dependent volcano plots to comprehend trends in OER activity.

**FIGURE 4:**
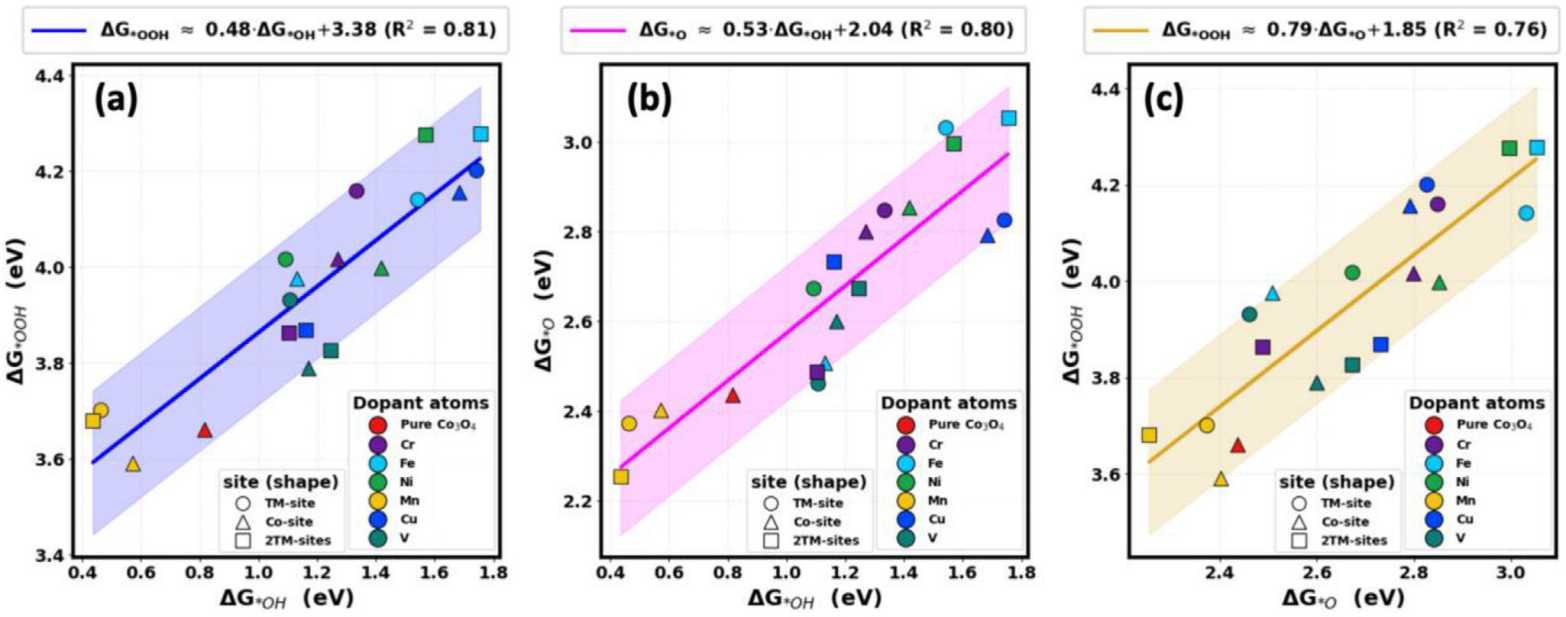
Linear scaling relationships between the OER intermediates (*OH, *O, and *OOH) for pristine and TM‐doped Co_3_O_4_. (a) ΔG_*OH_ vs. ΔG_*OOH_, (b) ΔG_*O_ vs. ΔG_*OH_, and (c). ΔG_*OOH_ vs. ΔG_*O_. Each data point corresponds to a specific dopant atom (Fe, Ni, Cr, Mn, Cu, V, or undoped Co_3_O_4_) and an active site type (TM, Co, and 2TM sites; cf. Figure 1c). The fitted regression lines are shown with confidence intervals (shaded regions), along with the slope, intercept, and coefficient of determination (R^2^).

Figure 4 shows linear scaling relationships between the adsorption free energies of *OH, *O, and *OOH on Co_3_O_4_ across the different dopants and site roles (TM, Co, 2TM). Although a certain degree of scatter can be observed in all three plots, they are still sufficiently linear based on the evaluation of the coefficient of determination. The scaling relationships in Figure 4 are then used to construct a potential‐dependent OER volcano diagram, the details of which are discussed in Section S4.

**FIGURE 5:**
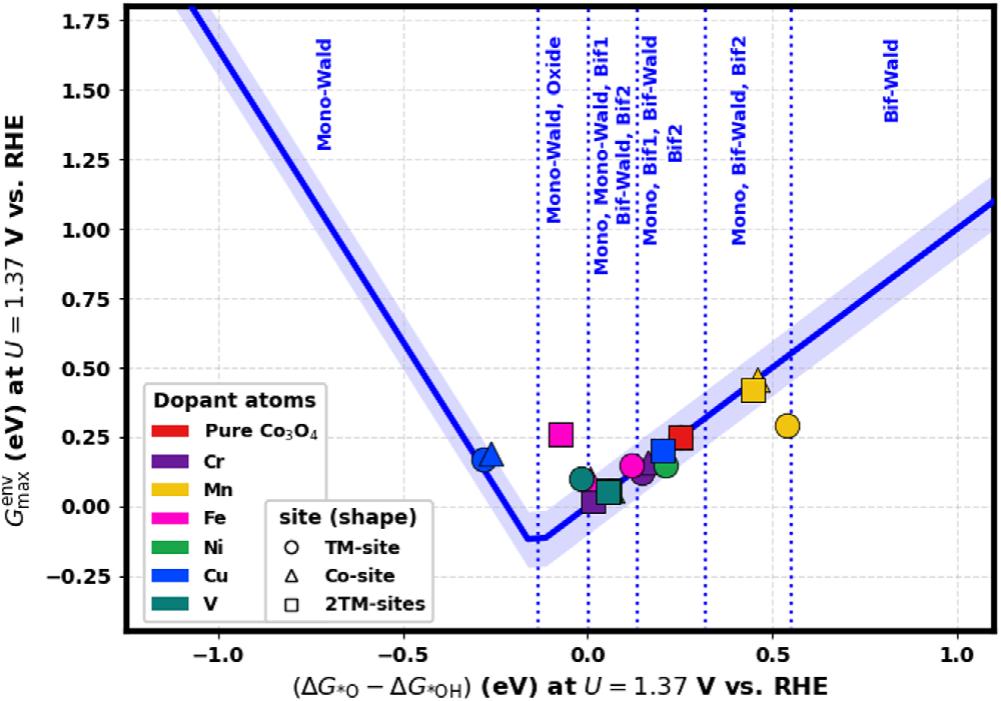
Volcano plot for the oxygen evolution reaction on doped Co_3_O_4_(001) models with additional information on the preferred reaction mechanism (marked in blue) as derived from the scaling relationships ΔG_*OOH_ vs. ΔG_*OH_ and ΔG_*O_ vs. ΔG_*OH_ at an applied electrode potential of *U* = 1.37 V vs. RHE. The solid line with shaded bands represents the predicted activity trends based on the scaling relationships, and the shaded area indicates uncertainty of the trend line based on the error bars of the scaling relationships. The vertical dashed lines mark transitions in the energetically favored reaction mechanism, and the preferred mechanistic description is given in each ΔG_*O_ − ΔG_*OH_ regime. Note that the symbols correspond to the DFT data (cf. Figure 2) for the different dopants (Cr, Fe, Ni, Mn, Cu, V, and undoped Co_3_O_4_) at different active sites (cf. Figure 1c).

Figure 5 shows a mechanistically resolved OER volcano plot at *U* = 1.37 V vs. RHE as a function of the descriptor ΔG_*O_ – ΔG_*OH_, while the solid line (“volcano line”) corresponds to the minimum free‐energy span (envelope $G_{\max}^{\mathrm{env}}\left(U=1.37V\right)$) across the seven reaction mechanisms, which is defined pointwise as

(3) $$G_{max}^{env}\;\left(U,\;\Delta G_{2}\right)=\min_{m}\;G_{max}^{\left(m\right)}\left(U,\;\Delta G_{2}\right).$$

In Equation (3), *m* refers to the respective mechanistic pathway, and Δ*G*_2_ = ΔG_*O_ − ΔG_*OH_. Further details on the construction of the $G_{max}^{env}\left(U=1.37V\right)$ envelope is provided in Figure S2 (cf. Section S2.4).

It is important to note that Figure 5 quantifies the uncertainty in the mechanistically resolved volcano plot by adding a narrow sensitivity band around the volcano trend line. This band results from the error bars of the scaling relationships in Figure 4. It is remarkable that of the 19 different configurations (three per dopant and undoped Co_3_O_4_), 17 fall within the band, and only two outliers (2TM‐site of Fe doping and TM‐site of Mn doping) are observed. This finding supports the suitability of our methodology to categorize electrocatalysts into active or inactive materials compared to a reference structure (i.e., undoped Co_3_O_4_) using an advanced volcano approach.

Figure 5 allows quantifying trends in OER activity for the different dopants: while Cr, Fe, Ni, Cu, and V improve the OER activity compared to undoped Co_3_O_4_ due to a smaller *G*_max_(*U*) value of the doped systems, which is supported by data points closer to the volcano apex, Mn deteriorates the OER performance. These trends are consistent with the analysis presented in Figure S9 in Section S3.

Finally, we comment on the progress of our method compared to conventional volcano analyses. The traditional OER volcano plot is derived based on the scaling relationship ΔG_*OOH_ vs. ΔG_*OH,_ assuming a slope of unity and an offset of 3.20 eV, whereas the scaling relationship ΔG_*O_ vs. ΔG_*OH_ is omitted from the analysis [5]. Activity predictions are rendered by applying the thermodynamic overpotential, η_*TD*_, instead of the span model of *G*_max_(*U*), although the η_*TD*_ descriptor does not allow for potential‐dependent activity analyses. Only the mononuclear mechanism on a single active site is evaluated, while other pathways are ignored. Figure S11 in Section S4 shows that the conventional volcano approach captures some of the activity trends observed in the volcano plot of Figure 5, such as the high activity of Cr‐doped Co_3_O_4_. On the other hand, it should be noted that almost 40% of the data points are not within the sensitivity band around the volcano trend line. In addition, several data points in the volcano in Figure S11 are located above the volcano apex. This observation is usually attributed to a broken scaling ΔG_*OOH_ vs. ΔG_*OH_ and thus to an improved OER performance [58].

We attribute the differences between the conventional volcano approach and the mechanistically resolved volcano diagram in Figure 5 to the omission of potential effects (assessment of activity trends under equilibrium conditions rather than OER conditions), the lack of mechanistic diversity (only use of the mononuclear mechanism), and a single active site in the traditional framework. Indeed, observing data points above the volcano apex and interpreting a broken scaling is erroneous, since our analysis (cf. Figure 4) reveals that the scaling relationship ΔG_*OOH_ vs. ΔG_*OH_ remains intact despite the introduction of dopants. Figure 5 clarifies that none of the doped Co_3_O_4_ models reveal activity above the volcano limit given by the $G_{\max}^{env}$ envelope, and multiple mechanisms govern the OER volcano apex [59], a situation not observed in the traditional approach (cf. Figure S11). Therefore, we conclude that mechanistically resolved trend studies under applied bias are needed to advance catalyst development in OER and avoid false claims of broken scaling relationships. The approach presented in this work can be extended to other OER material classes or electrocatalytic reactions of interest.

### 3.4 Comparison With Bulk‐Doped Co_3_O_4_

Several experimental works in the literature have investigated doping effects in the OER over Co_3_O_4_ [27, 28, 42, 43, 44, 45, 46]. It is often challenging to map these investigations to theoretically calculated volcano plots based on DFT, since most experimental studies are based on polycrystalline samples rather than single‐crystal electrodes. Among the various works, we highlight the contribution by Wei et al. [24] who showed, based on a combination of experiment and theory, that V doping enhances OER activity of Co_3_O_4_. This finding can be explained by the auxiliary site function of the V dopant on the (001) facet of Co_3_O_4_ (cf. Figure 3).

A different situation is encountered with the nanoparticle series reported in a recent work by Schulz and coworkers [27]. There, the authors examined M_x_Co_3‐x_O_4_ with M = Al, V, Cr, Mn, Fe, and Ni at comparable size and morphology with dopant concentrations of 1.6 at%, 3.3 at%, and 6.7 at%. Given that this series of nanoparticles might expose the (001) facet of Co_3_O_4_ to the same extent per material and the dopant concentration is in the same order of magnitude as in our theoretical framework, a qualitative comparison of our theoretical results with the experimental data of Schulz and coworkers is useful [27, 29, 37]. The authors observed increased OER activity for V, Cr, Fe, and Ni as dopants, while OER activity decreased for Mn. Note that Cu as a dopant wasn't investigated in this series, whereas Al as a dopant showed reduced OER activity. Among the dopants with a positive catalytic effect on OER activity, the activity trends for these spherical particles at a dopant concentration of 3.3% are V ≥ Cr > Fe > Ni.

We find that the qualitative activity trends of the V, Cr, Fe, Ni, and Mn dopants on the OER activity of Co_3_O_4,_ based on the experimental data [27] are consistent with our theoretical prediction. On the other hand, the volcano plot of Figure 5 (or Figure S9 in section S3) predicts a quantitative ranking of Cr > V > Ni > Fe. This illustrates that the quantitative trend in OER activity is not entirely reproduced by DFT calculations. We relate these minor deviations to the finding that the nanoparticle series of Schulz and coworkers is based on bulk doping, whereas our atomic‐scale models rely on surface doping (cf. Figure 1c). To further quantify the predictive nature of our theoretical model, we compare the trends of OER activity with surface‐doped Co_3_O_4_ samples subjected to pulsed laser treatment (cf. Section 3.5) and quantify the promoting effect of Cr doping by synthesizing surface‐enriched Cr‐Co_3_O_4_ (cf. Section 3.6).

### 3.5 Comparison With Pulse‐Optimized Samples of Surface‐Doped Co_3_O_4_

Surface‑doped Co_3_O_4_ nanoparticles were prepared via pulsed laser defect engineering in liquids (PUDEL; further details are available in Section S6) [60]. V, Cr, Ni, and Fe were introduced from aqueous metal‐‑salt precursor solutions by applying defined numbers of laser pulses to the Co_3_O_4_ catalyst dispersion in liquid flow. Cation dopants can be introduced into metal oxides using pulsed lasers at low energies and fluences, inducing short, transient heating of the nanoparticles without exceeding their transformation temperature. For Co_3_O_4_, this critical temperature was calculated to correspond to a laser intensity of 1.2 × 10^11^ W m^−2^, that is, 800°C [61]. Under such kinetically controlled conditions [62], the laser‑induced doping process proceeds in a largely isomorphic manner, as demonstrated for several oxide nanoparticle systems including Co_3_O_4_ [61], TiO_2_ [63], and CoFe_2_O_4_ [64].

Because the spinel phase of Co_3_O_4_ is preserved up to at least three PPV and the formation of side phases can be suppressed by cation incorporation [65], the PUDEL‑based dopant series can be regarded as an isomorphic doping series. This allows dopant‑induced changes in catalytic properties to be compared at similar nanoparticle diameters (cf. Figure S13). Depth‑profiling and doping mechanism studies further support a surface‑localized doping regime under these conditions, reaching effective doping depths of approximately 1.25 and 1.50 nm for two and three pulses per volume, respectively [30].

Thus, the PUDEL‑treated, surface‑doped Co_3_O_4_ nanoparticles provide a suitable experimental analogue for a qualitative comparison with our theoretical predictions (cf. Figure 5). Dopant concentrations were optimized individually to maximize the promotional effect per element. X‑ray fluorescence (XRF) analysis revealed higher cation incorporation for iron (3.7 %) and vanadium (2.77 %) compared to chromium (0.16 %) and nickel (below detection limit), likely reflecting differences in incorporation kinetics as well as steric or valence effects.

**FIGURE 6:**
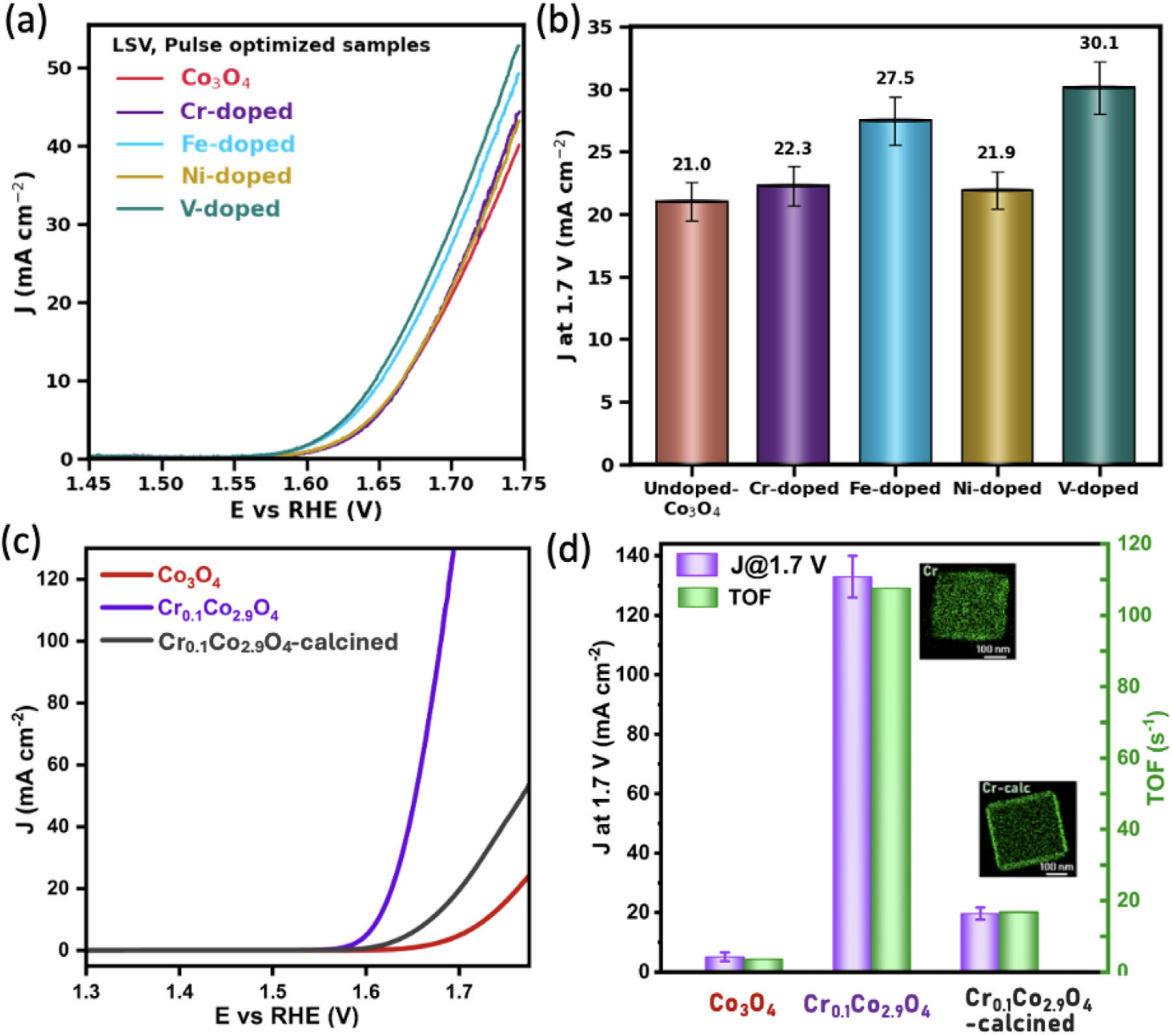
Electrocatalytic OER performance of doped Co_3_O_4_. (a) Linear sweep voltammograms (LSVs) in the OER potential range scanned with a rate of 5 mV s^−1^. Dopants were introduced by laser pulse treatment: 3 pulses per volume (PPV) for Cr, Ni, and V, and 2 PPV for Fe. Electrocatalytic measurements were performed in 1 M KOH with a rotating disk electrode (RDE), Pt/C as the counter electrode, and Ag/AgCl (3 M KCl) as the reference electrode. (b) Current density at 1.70 V vs. RHE. (c) LSVs (*iR*‐compensated) obtained for Co_3_O_4,_ Cr_0.1_Co_2.9_O_4,_ and Cr_0.1_Co_2.9_O_4_‐calcined catalysts in 1.0 M KOH solution at a scan rate of 5 mV s^−1^ and under 1600 rpm rotation of the rotating disc electrode, adapted from our ChemRxiv preprint [37] and (d) Corresponding measured current density for the three electrodes as well as the respective TOF values obtained at 1.7 V vs. RHE; Inset shows EDX elemental mapping of Cr‐doped samples.

All doped samples exhibited increased current densities and hence improved electrocatalytic performance in the OER compared to undoped Co_3_O_4_, confirming the positive effect of these transition metals on OER activity. In rotating disk electrode measurements at 1.70 V vs. RHE for drop‑casted catalysts, the activity trend in terms of increased current density was V > Fe > Cr > Ni, which is possibly linked to variable ion incorporation into the spinel lattice (cf. Figure 6a,b). Small additions of Ni and Cr led to at most modest changes, which remain within experimental uncertainty at higher electrode potentials. For catalysts with higher dopant contents, deviations from the undoped baseline become apparent at 1.60 V vs. RHE. Interestingly, the Tafel slopes (Table S5) indicate a similar apparent reaction pathway for all samples, whereas kinetic differs, as reflected by the dopant‐induced increases in current density. This kinetic improvement can reasonably be attributed to a reduction in charge‑transfer resistance, as evidenced by the electrochemical impedance spectroscopy (EIS) results summarized in Table S6. The lower charge‑transfer resistance facilitates more efficient electron transfer at the electrode–electrolyte interface, thereby promoting faster reaction rates under the applied overpotential. Collectively, these findings indicate that while the overall reaction mechanism remains unchanged, doping effectively enhances the charge‑transfer properties of the material, leading to improved catalytic activity. We note that the activity trend of the laser‑treated surface‑doped Co_3_O_4_ nanoparticles differs from that of the bulk‑doped Co_3_O_4_ samples discussed in Section 3.4. This result is relevant for comparing theoretical studies on dopant effects with experimentally tested materials, since a thorough investigation of bulk‐doped materials is beyond the scope of conventional DFT models due to the limitation to a few hundred atoms. In contrast, surface‐doped electrocatalysts can be described reasonably well by theoretical models such as the one used in this study (cf. Figure 1).

Within this framework theory correctly predicts that all dopants lead to improved OER activity relative to undoped Co_3_O_4_, in qualitative agreement with both bulk‑ and surface‑doped experimental series. However, the quantitative activity ranking from the volcano plot (Cr > V > Ni > Fe; Figures 5 and S9) cannot be fully reproduced. These discrepancies likely arise from differences between the idealized, periodically doped model surface and the real catalyst environment, including local dopant concentration and spatial distribution at the surface as well as the electrochemically active surface area and facet distribution. Experiments show positive effects of cation doping, leading to increased OER activity, reduced charge transfer resistance, and lower overpotentials compared to undoped Co_3_O_4_ (cf. Section S9.1). While a quantitative one‑to‑one comparison between theory and experiment is not possible, since both the doping and activity depend on multiple, partially coupled parameters, qualitative trends, such as the identification of auxiliary promoters that result in enhanced reaction kinetics, as particularly evident for the case of V (cf. Section 3.2), remain robust. These trends are valuable for rational selection of dopants in experimental studies.

### 3.6 Experimental Verification of the Promoted OER Activity of Cr‐Doped Co_3_O_4_

Figure 3 indicates that Cr‐doped Co_3_O_4_ reveals the main promoting effect in the OER, particularly if the dopant concentration is increased. This theoretical prediction is verified by synthesizing Cr‐doped, surface‐enriched Co_3_O_4_ nanocubes using a hydrothermal method [37]. Details on synthesis and characterization can be found in Section S7. Importantly, particles of well‐defined Co_3_O_4_ and Cr‐substituted Cr_0.1_Co_2.9_O_4_ nanocubes were microscopically and spectroscopically characterized and showed the same well‐defined cubic shape and size (ca. 250 nm edge length), as shown in Figure S15 (cf. Section S7). Cr content in the bulk (from EDX) is 4.4% for both pristine and calcined Cr_0.1_Co_2.9_O_4_, whilst the surface Cr content (from XPS) is 36% and 46% for Cr_0.1_Co_2.9_O_4_ and calcined Cr_0.1_Co_2.9_O_4_, respectively [37]. We note that Cr doping results in a phase segregation, which leads to the formation of a partially Cr‐doped Co_3_O_4_ and a Cr‐rich surface oxide phase. The linear sweep voltammetry (LSV) curves in Figure 6c show that Cr_0.1_Co_2.9_O_4_ exhibits a much higher OER current than undoped Co_3_O_4_, confirming that the incorporation of Cr into the surface of Co_3_O_4_ substantially enhances the catalytic performance. After calcination of the pristine Cr_0.1_Co_2.9_O_4_ nanocubes, the Cr_0.1_Co_2.9_O_4_‐calcined sample maintains its cubic shape however shows Cr enrichment at the nanoparticle surface than in the bulk, as evidenced from TEM‐EDX elemental mapping and line‐scan analysis (cf. Figure S16). The TEM line scan (Figure S16) shows a Cr‐enriched shell of approximately 30 nm in the ca. 270 nm large Cr_0.1_Co_2.9_O_4_‐calcined nanocube. Quantitative analysis of the EDX data [37] revealed that the Cr/(Co+Cr) ratio reached up to 40%–50% at the cube edges, compared to only 3% in the bulk. This composition mirrors the surface model used for the computations (cf. Figure 1), where 50% Cr doping on the Co_3_O_4_ surface was predicted to increase the OER activity by several orders of magnitude due to a reduction in the Δ*G*_max_ value (cf. Figure 3).

As shown in Figure 6d, the current density at 1.70 V versus RHE increased by about five and 25 times for Cr_0.1_Co_2.9_O_4_‐calcined and Cr_0.1_Co_2.9_O_4_ compared to the undoped Co_3_O_4_, respectively. The corresponding turnover frequency (TOF) values, calculated assuming that only surface Co atoms on the five cube facets are active [37], also follow this trend: Cr‐doped samples exhibit a significantly higher TOF than Co_3_O_4_. Further, Cr‐doped Co_3_O_4_ samples exhibit significantly smaller Tafel slope and charge‐transfer resistance (*R*_ct_) than the undoped Co_3_O_4_, as summarized in Table S7, suggesting improved intrinsic kinetics and charge transfer properties. Notably, the particles retained their well‐defined cubic morphology and particle size after Cr incorporation (Figures S15–S16), suggesting that the dopant nature and distribution play the dominant role. Based on our XPS analysis [37], the observed enhancement is attributed to Cr‐induced modulation of the surface electronic structure, which promotes the formation of more Co^2+^ species readily, which can be easily converted to the active oxyhydroxide phase with octahedral Co^3^^+^ sites under OER conditions [66, 67]. Moreover, post‐OER characterization of the cubic particles using SEM shows no obvious morphological or particle size changes (see Figure S17) as well as limited Co and Cr leaching (<100 µg L^−1^) after 2 h of OER operation, supporting the relevance of these particles to the DFT model. The experimental results demonstrate that surface doping of Co_3_O_4_ by Cr enhances the intrinsic activity by modulating the surface and electronic structure in a manner favorable for OER, thus confirming the theoretical prediction (cf. Figure 3).

## 4 Conclusion

In the present manuscript, we present a theoretical framework based on electronic structure theory calculations using density functional theory to understand dopant effects in the OER over Co_3_O_4_. By further developing the concept of volcano plots toward mechanistically resolved volcano diagrams that consider multiple scaling relationships, multiple configurations of active sites, and potential‐dependent activity quantification (cf. Figure 5), we find that the dopants V, Cr, Fe, and Ni improve the OER activity of Co_3_O_4_, while doping with Mn or Cu leads to a reduction in OER performance or a negligible contribution, respectively. In contrast to previous theoretical works on this topic, we analyze the promoting effects of the different foreign metals and identify Cr as the most promising dopant (cf. Figure 3), since Cr efficiently enhances the electrocatalytic activity at the auxiliary site, the active site, and at higher dopant concentrations.

To benchmark our theoretical calculations, we compare the predicted trends of OER activity of the doped Co_3_O_4_ models with two different series of Co_3_O_4_ nanoparticles. One of the series is based on the bulk doping of Co_3_O_4_ [27], while the other considers surface doping followed by laser treatment [60]. We find that our theoretical framework accurately captures the qualitative impact of dopants on OER activity, while the quantitative trends are not fully reproduced. This is consistent with the original goal of the present DFT framework, which is primarily intended for materials screening, that is, identifying whether dopant substitution enhances or suppresses OER activity relative to pristine Co_3_O_4_ rather than predicting absolute catalytic currents. We attribute this finding to the obvious size gap between atomic‐scale models and experimental Co_3_O_4_ catalysts at the nanoscale. We experimentally validate the theoretical prediction of the promoting effect of Cr‐doped Co_3_O_4_ by synthesizing a well‐defined surface‐enriched, Cr‐doped Co_3_O_4_ nanocube that significantly enhances the OER activity compared to undoped Co_3_O_4_. Even at far smaller dopant concentrations, surface doping of Cr into Co_3_O_4_ nanoparticles by laser pulsing yielded measurable enhancement.

The implications of our work are two‐fold: first, the reported approach for quantifying activity trends of doped Co_3_O_4_ models is a transferable framework that can be equally applied to other materials and electrocatalytic processes. In particular, the potential‐dependent volcano plot accounts for the uncertainty in the analysis of adsorption free energies and, compared to the conventional approach, avoids the assertion of broken scaling relationships, which could lead to erroneous interpretation of the OER performance. Second, we argue that inspecting the promoting effects of dopants is a suitable approach to bridge the size gap between atomic‐scale models and experimental catalysts and claim that this may be a better way to unambiguously compare theoretical predictions with experimental data. Our work could therefore pave the way for next‐generation theoretical models aimed at catalyst screening in energy conversion and storage, using uncertainty control in volcano plots and promoting effects as levers for the development of advanced catalytic materials.

## Conflicts of Interest

The authors declare no conflicts of interest.

## Supporting information




**Supporting File**: anie72456‐sup‐0001‐SuppMat.pdf.

## Data Availability

The data that support the findings of this study are available in the Supporting Information of this article.
