# Supplementary material for: Unifying Scaling Relations and Multiple Reaction Mechanisms for Screening Transition Metal‐Doped Co3O4 for Oxygen Evolution Reaction
Source: Angew Chem Int Ed Engl. 2026 May 4;65(26):e24523. doi: 10.1002/anie.202524523 (PMC13285473; doi:10.1002/anie.202524523)
Supplement: Supplementary file 1 — Supporting File: anie72456‐sup‐0001‐SuppMat.pdf. [file ANIE-65-e24523-s001.pdf]

Supporting Information

Unifying Scaling Relations and Multiple Reaction Mechanisms for Screening Transition Metal-Doped Co_3O_4 for Oxygen Evolution Reaction

Kapil Dhaka,[1] Hatem M.A. Amin,[2] Davide Beschi,[3] Dana Schellenburg,[3] Benjamin Mockenhaupt,[3] Stephan Barcikowski,[3,4] Stephan Schulz,[2,4] Kai S. Exner[1,4,5,*]

[1] *University of Duisburg-Essen, Faculty of Chemistry, Theoretical Catalysis and Electrochemistry, Universitätsstraße 5, 45141 Essen, Germany*

[2] *University of Duisburg-Essen, Faculty of Chemistry, Institute of Inorganic Chemistry, Universitätsstraße 5, 45141 Essen, Germany*

[3] *University of Duisburg-Essen, Faculty of Chemistry, Technical Chemistry I, Universitätsstraße 5, 45141 Essen, Germany*

[4] *Center for Nanointegration (CENIDE) Duisburg-Essen, 47057 Duisburg, Germany*

[5] *Cluster of Excellence RESOLV, 44801 Bochum, Germany*

* Corresponding author: kai.exner@uni-due.de ORCID: 0000-0003-2934-6075 (KSE)

S1 Computational Details

All density functional theory (DFT) calculations were carried out using the Vienna Ab initio Simulation Package (VASP). [1–3] We employed a 2x2 Co_3O_4(001)-4*OH slab of termination B, which exposes four surface octahedral cobalt sites, Co^{oct}. The bare 2x2 Co_3O_4(001) termination without adsorbate coverage is shown in **Figure S1**. For the thermodynamic analysis of the free-energy changes in the oxygen evolution reaction (OER), we then used a 4*OH-covered surface model: one or two of the four Co^{oct} sites were systematically substituted by 3d transition-metal dopants (cf. **Figure 1** in the main text).

Exchange–correlation effects were treated with the Perdew–Burke–Ernzerhof (PBE) functional[4] within the generalized gradient approximation (GGA). To properly describe the localized Co 3d states, we used the PBE+U method in the Dudarev formulation (U_{eff}).[5] Effective U values were set to 3.0 eV for Co,[6] 1.0 eV for V,[7] 3.7 eV for Cr,[8] 3.5 eV for Mn,[9] 4.5 eV for Fe,[10] 7.0 eV for Cu,[11] and 3.8 eV for Ni.[12] Long-range interactions were captured by Grimme's D3 dispersion correction[13] to account for van der Waals forces, which are particularly relevant for weakly bound intermediates and solvent molecules. The projector augmented wave (PAW) method[14] was employed to describe core–valence interactions, with a plane-wave cutoff energy set to 550 eV.

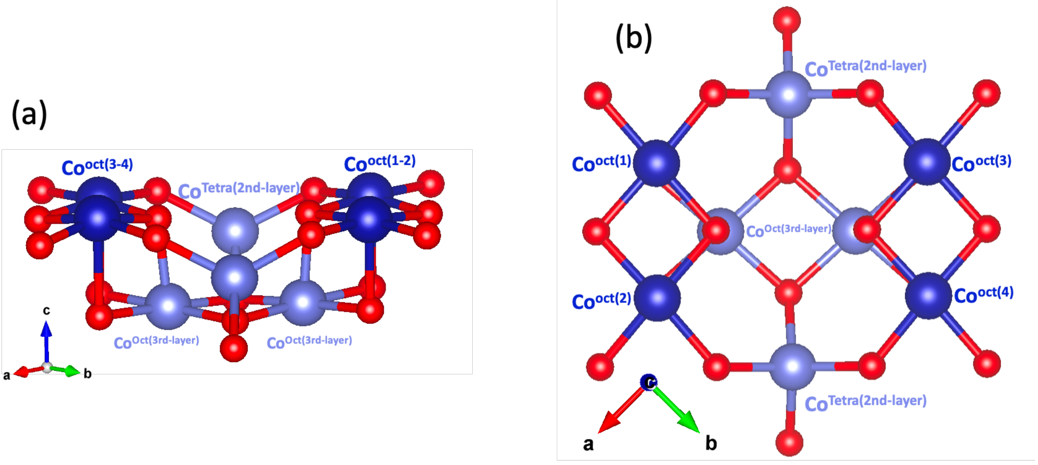

Figure S1. Bare 2×2 Co_3O_4(001) slab highlighting the four surface octahedral cobalt sites, Co_{oct} (1 to 4), where adsorption processes take place under anodic polarization. (a) Side view and (b) Top view. Subsurface cobalt atoms are indicated for context: Co_{tetra} in the 2nd layer and Co_{oct} in the 3rd layer. Oxygen and cobalt atoms are shown in red and blue, respectively.

Geometry optimizations were performed until the total energy change was below 10^{-5} eV and atomic forces were reduced to less than 0.03 eV Å^{-1}. Brillouin-zone sampling was conducted using a Γ-centered 3×3×1 k-point mesh. Solvation and electrochemical interface effects were approximated with the VASPsol implicit solvent model,[15] where the relative dielectric constant was set to 78.4 (representing liquid water at room temperature). Ionic screening was mimicked using a Debye length of 3.0 Å.

Vibrational frequency analyses were performed for all adsorbed species to evaluate zero-point energy (ZPE) and entropic contributions. The entropies were derived solely from vibrational modes of surface-bound intermediates. ZPE and entropy corrections were obtained from the following standard expressions:

$$E_{ZPE} = \frac{1}{2}\sum_{i} h\nu_i \tag{S1}$$

$$TS = k_B T \sum_{i}^{n} \left[\frac{\frac{h\nu_i}{k_B T}}{e^{\frac{h\nu_i}{k_B T}} - 1} - ln\left(1 - e^{\frac{-h\nu_i}{k_B T}}\right) \right] \tag{S2}$$

where k_B, h, ν_i, n, and T denote the Boltzmann constant, Planck constant, vibrational frequency, number of modes, and temperature, respectively. Free energies were then calculated by including both ZPE and entropic terms according to:

$$G = E_{DFT} + E_{ZPE} - TS \tag{S3}$$

We employ the computational hydrogen electrode (CHE) approach[16], where the reference state for a proton-electron pair at $U = 0$ V vs. RHE (reversible hydrogen electrode), $T = 298.15$

K, and p_{H_2} = 1 atm is related to gaseous hydrogen by referring to the equilibrium of equation (4):

$$H^+_{(aq)} + e^- \rightleftharpoons \frac{1}{2} H_{2(g)} \quad \text{(S4)}$$

In addition to gaseous hydrogen, gaseous water at T = 298.15 K and p_{H_2O} = 0.035 atm is used as another reference state in our analysis, as gaseous water and liquid water are in equilibrium under these conditions. Further details on the modeling of the elementary reaction steps of OER are provided in section S2 of the SI.

We systematically tested multiple initial antiferromagnetic arrangements and different initial local magnetic moments on surface Co atoms and dopant species for representative pristine and doped surface motifs and key adsorbate-covered states to obtain the lowest-energy solution.

S2. Scaling Relations, OER Mechanisms, and Volcano Plots

S2.1 Mononuclear mechanism and scaling relations

The elementary reaction steps of the OER are usually described in the literature by referring to the mononuclear description. This pathway contains the adsorbed intermediates *OH, *O, and *OOH, which are formed subsequently by proton-coupled electron transfer steps according to equations (S5) – (S8).

$$* + H_2O_{(l)} \rightarrow *OH + (H^+ + e^-),\ \Delta G_1 \quad \text{(S5)}$$

$$*OH \rightarrow *O + (H^+ + e^-),\ \Delta G_2 \quad \text{(S6)}$$

$$*O + H_2O_{(l)} \rightarrow *OOH + (H^+ + e^-),\ \Delta G_3 \quad \text{(S7)}$$

$$*OOH \rightarrow * + (H^+ + e^-) + O_{2(g)},\ \Delta G_4 \quad \text{(S8)}$$

The sum of the four free-energy changes amounts to 4.92 eV at U = 0 V vs RHE (reversible hydrogen electrode).

Based on the free-energy changes of the mononuclear description, one can express the free energies of the intermediate states *OH, *O, and *OOH (with respect to the vacant active site):

$$\Delta G_{*} = 0 \text{ eV} \quad \text{(S9)}$$

$$\Delta G_{*OH} = \Delta G_1 - 1eU \quad \text{(S10)}$$

$$\Delta G_{*O} = \Delta G_1 + \Delta G_2 - 2eU \quad \text{(S11)}$$

$$\Delta G_{*OOH} = \Delta G_1 + \Delta G_2 + \Delta G_3 - 3eU \quad \text{(S12)}$$

$$\Delta G_{*+O2} = 4.92 \text{ eV} - 4eU \quad \text{(S13)}$$

The traditional scaling-relation approach correlates the free energies of *O and *OOH as linear functions of *OH. For the dataset of the doped $Co_3O_4(001)$-4*OH surface, the two fitted linear relations are:

$$\Delta G_{*O} = m_1\,\Delta G_{*OH} + b_1 \tag{S14}$$

$$\Delta G_{*OOH} = m_2\,\Delta G_{*OH} + b_2 \tag{S15}$$

In equations (S14) and (S15), m_1 and m_2 are the slopes and b_1 and b_2 are the intercepts of the corresponding scaling relationships. Note that their values can be extracted from **Figure 4a-b** in the main text.

S2.2 From adsorption free energies to scaling-dependent free energies

Equations (S14) – (S15) allow us to rewrite the free-energy changes of the elementary steps entirely in terms of a single descriptor — namely $\Delta G_2 = \Delta G_{*O} - \Delta G_{*OH}$ — and the fitted parameters m_1, m_2, b_1, and b_2. We arrive at the following expressions:

$$\Delta G_{*OH} = \frac{1}{m_1 - 1}(\Delta G_2 - b_1) \tag{S16}$$

$$\Delta G_{*O} = \frac{m_1}{m_1 - 1}(\Delta G_2 - b_1) + b_1 \tag{S17}$$

$$\Delta G_{*OOH} = \frac{m_2}{m_1 - 1}(\Delta G_2 - b_1) + b_2 \tag{S18}$$

By substituting equations (S16) – (S18) into equations (S9) – (S13) we obtain explicit expressions for ΔG_{*OH}, ΔG_{*O}, and ΔG_{*OOH} as a function of ΔG_2 and the fitted coefficients m_1, m_2, b_1, and b_2. This conversion turns the scaling relationships into predictive, potential-dependent scaling-dependent free energies.

S2.3 Generalization to multiple reaction mechanisms

While the mononuclear mechanism serves as the basis for scaling relationships, complex oxide surfaces, especially doped ones, can also operate through alternative pathways such as Walden-type mechanisms,[17] bifunctional variants,[18–21] binuclear routes,[22,23] and oxide pathways.[24,25] In each case, there are four proton-coupled electron transfer steps, but the way in which intermediate states pair between successive states is different. The conversion procedure from adsorption free energies to scaling-dependent free energies remains the same:

1. Choose the descriptor (we retain $\Delta G_2 = \Delta G_{*O} - \Delta G_{*OH}$).
2. Use equations (S9) – (S15) to express ΔG_{*OH}, ΔG_{*O}, and ΔG_{*OOH} in terms of ΔG_2, m_1, m_2, b_1, and b_2.

3. Add or subtract the relevant intermediate free energies according to the mechanistic sequence. The reaction cycle is closed by considering that the free energy of the last intermediate state amounts to 4.92 eV at U = 0 V vs RHE.

In the following, we provide the respective equations for the scaling-dependent free energies of all seven pathways considered in this work. Note that the following equations compile the free-energy changes of the elementary steps, ΔG_j (j = 1, …, 31), by making use of the conversion scheme discussed above. These free-energy changes can be translated to the free energies of the intermediate states by using the correlations in equations (S9) – (S13).

Mononuclear mechanism:

Single-site pathway via *OH, *O, OOH. The asterisk (*) denotes the active Co/TM site.[26,27]

* + $H_2O_{(l)}$ ➔ *OH + (H^+ + e^-)	$\Delta G_1(U) \cong \frac{\Delta G_2 - b_1}{m_1 - 1} - eU$	(S19)
*OH ➔ *O + (H^+ + e^-)	$\Delta G_2(U) = \Delta G_2 - eU$	(S20)
*O + $H_2O_{(l)}$ ➔ *OOH + (H^+ + e^-)	$\Delta G_3(U) \cong \frac{m_2 - 1}{m_1 - 1}(\Delta G_2 - b_1) + b_2 - \Delta G_2 - eU$	(S21)
*OOH ➔ * + (H^+ + e^-) + $O_{2(g)}$	$\Delta G_4(U) \cong 4.92 - \frac{m_2}{m_1 - 1}(\Delta G_2 - b_1) - b_2 - eU$	(S22)

Bifunctional-I mechanism:

In the context of this work, the term "bifunctional" refers to mechanisms in which two distinct surface sites cooperatively participate in the catalytic cycle.

In contrast to the mononuclear mechanism, where all elementary steps occur at a single metal center, the bifunctional mechanism involves (i) a primary active site that binds oxygenated intermediates (*OH, *O, *OOH, *OO) and (ii) a neighboring auxiliary site (*O_A sites) that facilitates proton transfer, hydrogen bonding, or intermediate stabilization. The auxiliary site often assists in proton-coupled electron transfer (PCET) steps by acting as a proton acceptor or hydrogen-bond donor.

Two sites are involved (* and a neighboring oxygen atom, *O_A). This pathway bypasses the *OOH intermediate through direct *OO formation:[21]

* + *O_A + $H_2O_{(l)}$ ➔ *OH+ *O_A + (H^+ + e^-)	$\Delta G_5(U) \cong \frac{\Delta G_2 - b_1}{m_1 - 1} - eU$	(S23)
*OH + *O_A ➔ *O + *O_A + (H^+ + e^-)	$\Delta G_6(U) = \Delta G_2 - eU$	(S24)
*O + *O_A + $H_2O_{(l)}$ ➔ *OO + *OH_A + (H^+ + e^-)	$\Delta G_7(U) \cong \frac{\Delta G_2 - b_1}{m_1 - 1} - eU$	(S25)

*OO + *OH$_A$ ➔ * + *O$_A$ + ($H^+ + e^-$) + $O_{2(g)}$	$\Delta G_8(U) \cong 4.92 - \left[2\frac{\Delta G_2 - b_1}{m_1 - 1} + \Delta G_2\right] - eU$	(S26)

Bifunctional-II mechanism:

Two sites (* and *O$_A$) are involved, and this pathway contains a non-electrochemical reaction step, in which water is adsorbed on the surface.[18–20] Therefore, the mechanistic sequence contains five steps in total:

* + *O$_A$ + $H_2O_{(l)}$ ➔ *OH+ *O$_A$ + ($H^+ + e^-$)	$\Delta G_9(U) \cong \frac{\Delta G_2 - b_1}{m_1 - 1} - eU$	(S27)
*OH + *O$_A$ ➔ *O + *O$_A$ + ($H^+ + e^-$)	$\Delta G_{10}(U) \cong \Delta G_2 - eU$	(S28)
*O + *O$_A$ + $H_2O_{(l)}$ ➔ *OOH + *OH$_A$	$\Delta G_{11}(U) \cong \frac{m_2 - 1}{m_1 - 1}(\Delta G_2 - b_1) + b_2 - 2\Delta G_2$	(S29)
*OOH + *OH$_A$ ➔ *OOH + *O$_A$ + ($H^+ + e^-$)	$\Delta G_{12}(U) \cong \Delta G_2 - eU$	(S30)
*OOH + *O$_A$ ➔ * + *O$_A$ + ($H^+ + e^-$) + $O_{2(g)}$	$\Delta G_{13}(U) \cong 4.92 - \frac{m_2}{m_1 - 1}(\Delta G_2 - b_1) - b_2 - eU$	(S31)

Binuclear mechanism:

Here, two adjacent metal surface sites work in tandem to facilitate O–O bond formation through the coupling of two adjacent oxygen adsorbates.[22,23]

* + * + $H_2O_{(l)}$ ➔ *OH+ *O + ($H^+ + e^-$)	$\Delta G_{14}(U) \cong \frac{\Delta G_2 - b_1}{m_1 - 1} - eU$	(S32)
*OH + * + $H_2O_{(l)}$ ➔ *OH + *OH + ($H^+ + e^-$)	$\Delta G_{15}(U) \cong \frac{\Delta G_2 - b_1}{m_1 - 1} - eU$	(S33)
*OH + *OH ➔ *O + *OH + ($H^+ + e^-$)	$\Delta G_{16}(U) \cong \Delta G_2 - eU$	(S34)
*O + *OH ➔ *O + *O + ($H^+ + e^-$)	$\Delta G_{17}(U) \cong \Delta G_2 - eU$	(S35)
*O + *O ➔ * + * + $O_{2(g)}$	$\Delta G_{18}(U) \cong 4.92 - \left[2\frac{\Delta G_2 - b_1}{m_1 - 1} + 2\Delta G_2\right]$	(S36)

Oxide mechanism:

This mechanism commences from an oxygen-covered surface and relies on the coupling of two adjacent *OO intermediates to form gaseous oxygen:[24,25]

*O + *O + $H_2O_{(l)}$ ➔ *OOH+ *O + ($H^+ + e^-$)	$\Delta G_{19}(U) \cong \frac{m_2 - 1}{m_1 - 1}(\Delta G_2 - b_1) + b_2 - \Delta G_2 - eU$	(S37)
*OOH + *O + $H_2O_{(l)}$➔ *OOH + *OOH + ($H^+ + e^-$)	$\Delta G_{20}(U) \cong \frac{m_2 - 1}{m_1 - 1}(\Delta G_2 - b_1) + b_2 - \Delta G_2 - eU$	(S38)
*OOH + *OOH ➔ *OOH + *OO + ($H^+ + e^-$)	$\Delta G_{21}(U) \cong \Delta G_2 - eU$	(S39)
*OOH + *OO ➔ *OO + *OO + ($H^+ + e^-$)	$\Delta G_{22}(U) \cong \Delta G_2 - eU$	(S40)

*OO + *OO ➔ *O + *O + $O_{2(g)}$	$\Delta G_{23}(U) \cong 4.92 - \left[2\frac{m_2 - 1}{m_1 - 1}(\Delta G_2 - b_1) + 2b_2\right]$	(S41)

In contrast to conventional mechanisms, Walden pathways are based on concerted O_2 desorption and H_2O adsorption;[28,29] this implies that the active Co/TM sites are always capped by an adsorbate. Note that binuclear and oxide Walden-type mechanisms are excluded for steric reasons due to the need of two adjacent metal sites in the description of the elementary steps.

Mononuclear-Walden mechanism:

This pathway can be categorized as a single-site Walden mechanism:

*OH ➔ *O + (H^+ + e^-)	$\Delta G_{24}(U) \cong \Delta G_2 - eU$	(S42)
*O + $H_2O_{(l)}$ ➔ *OOH + (H^+ + e^-)	$\Delta G_{25}(U) \cong \frac{m_2 - 1}{m_1 - 1}(\Delta G_2 - b_1) + b_2 - eU$	(S43)
*OOH ➔ *OO + (H^+ + e^-)	$\Delta G_{26}(U) \cong \Delta G_2 - eU$	(S44)
*OO + $H_2O_{(l)}$ ➔ *OH + (H^+ +e^-) +$O_{2(g)}$	$\Delta G_{27}(U) \cong 4.92 - \left[\Delta G_2 + \frac{m_2 - 1}{m_1 - 1}(\Delta G_2 - b_1) + b_2\right] - eU$	(S45)

Bifunctional-Walden mechanism:

While this mechanistic pathway is reminiscent of the bifunctional-I description, it can be categorized as a dual-site Walden mechanism:

*OH + $*O_A$ ➔ *O + $*O_A$ + (H^+ + e^-)	$\Delta G_{28}(U) \cong \Delta G_2 - eU$	(S46)
*O + $*O_A$ + $H_2O_{(l)}$ ➔ *OO + $*OH_A$ + (H^+ + e^-)	$\Delta G_{29}(U) \cong \frac{\Delta G_2 - b_1}{m_1 - 1} - eU$	(S47)
*OO + $*OH_A$ ➔ *OO + $*O_A$ + (H^+ + e^-)	$\Delta G_{30}(U) \cong \Delta G_2 - eU$	(S48)
*OO + $*O_A$ + $H_2O_{(l)}$ ➔ *OH + $*O_A$ +(H^+ +e^-) +$O_{2(g)}$	$\Delta G_{31}(U) \cong 4.92 - \left[2\Delta G_2 + \frac{\Delta G_2 - b_1}{m_1 - 1}\right] - eU$	(S49)

2.4 Selection of OER Mechanisms and Treatment of Lattice Oxygen Participation

Seven OER mechanisms were considered in this work to systematically represent the principal mechanistic classes proposed for alkaline OER on transition-metal oxides within a surface-resolved framework. These include mononuclear, bifunctional, binuclear, oxide-type, and Walden-type pathways, thereby covering single-site, cooperative multi-site, and surface-oxygen-assisted O-O bond formation scenarios.[17,30,31]

In the literature, the lattice oxygen mechanism (LOM) is often discussed for spinel oxides.[32] However, two distinct concepts are typically grouped under this term: (i) surface oxygen-assisted pathways, where a surface oxygen atom participates in O-O bond formation while the lattice remains intact, and (ii) true lattice oxygen exchange mechanisms involving oxygen vacancy formation and oxygen exchange between the lattice and the electrolyte.

In the present study, surface oxygen participation is already explicitly included through the bifunctional mechanisms, where surface oxygen atoms act as proton acceptors or cooperate in O-O bond formation. These pathways do not involve lattice oxygen removal or vacancy cycling, and the lattice framework remains preserved along the reaction coordinate. A full LOM treatment would require explicit modeling of oxygen vacancy formation, oxygen exchange steps, and defect thermodynamics, which introduce additional variables beyond the scope of the descriptor-based volcano framework employed here.

2.5 Relation to Previous Studies on Doped Co_3O_4 OER Catalysts

Previous computational studies[33–35] have investigated transition-metal-doped Co_3O_4 catalysts for the oxygen evolution reaction (OER). While these works have provided important insight into dopant effects, they typically focus on a limited number of dopant elements and a single mechanistic description of the reaction.

The present work extends these earlier studies in several aspects. First, the chemical space of investigated dopants is systematically expanded to six transition metals (Cr, Mn, Fe, Ni, Cu, and V). This broader screening enables a more comprehensive assessment of how different substitutions influence catalytic activity trends within the Co_3O_4 host lattice.

Second, the present study explicitly distinguishes between three different local dopant configurations: TM, Co, and 2TM. These configurations represent distinct dopant–environment interactions and allow us to evaluate whether the dopant acts directly as the catalytic center (TM case), modifies the electronic properties of a neighboring Co site (Co case), or participates in cooperative interactions between adjacent dopant atoms (2TM case). This motif-resolved analysis provides additional atomistic insight into dopant-induced promotion effects.

Third, instead of restricting the analysis to a single OER pathway, multiple mechanistic scenarios are considered. This allows the investigation of potential-dependent mechanistic

crossovers and reveals how the preferred reaction pathway may vary depending on the dopant environment and applied potential.

Fourth, the catalytic activity is analyzed using a free-energy-span-based descriptor, $G_{\max}(U)$, which enables construction of potential-dependent volcano plots. In contrast to traditional scaling-based volcano relationships, this framework explicitly accounts for all elementary reaction steps and identifies the dominant kinetic bottleneck under operating conditions.

Finally, the present work integrates experimental measurements for doped Co_3O_4 catalysts, enabling direct comparison between theoretical predictions and experimentally observed activity trends. The combined theoretical–experimental analysis provides a mechanistically resolved interpretation of dopant-induced promotion effects and allows identification of promising dopants within the investigated series.

S2.6 Construction of volcano plots

At a fixed electrode potential U, the electrocatalytic activity for each pathway is governed by the $G_{\max}(U)$ descriptor (cf. equation (1) in the main text). We use $G_{max}^{(m)}$ to indicate the $G_{\max}(U)$ value for a given reaction mechanism (m). In order to identify which reaction mechanisms controls the volcano line in the volcano plot in dependence of the descriptor ΔG_2, we analyze equation (S50):

$$G_{max}^{env}(U,\ \Delta G_2) = \min_{m} G_{max}^{(m)}(U,\ \Delta G_2) \qquad \text{(S50)}$$

In equation (S50), $G_{max}^{env}(U,\ \Delta G_2)$ corresponds to the 'envelope' (volcano line), which serves as an upper boundary of the electrocatalytic activity. We visualize the corresponding procedure in **Figure S2** at two different electrode potentials, namely U = 1.23 V and 1.37 V vs RHE. There it becomes evident that in different free-energy intervals of the ΔG_2 descriptor, different reaction mechanisms are favored for the doped Co_3O_4(001)-4*OH surface. The reaction mechanisms with the lowest free energy form the envelope that serves as a proxy for the electrocatalytic activity. We discuss the mechanistically resolved, potential-dependent volcano plot further in section S5 of the SI when comparing this framework with the conventional volcano approach (cf. **Figure S11**).

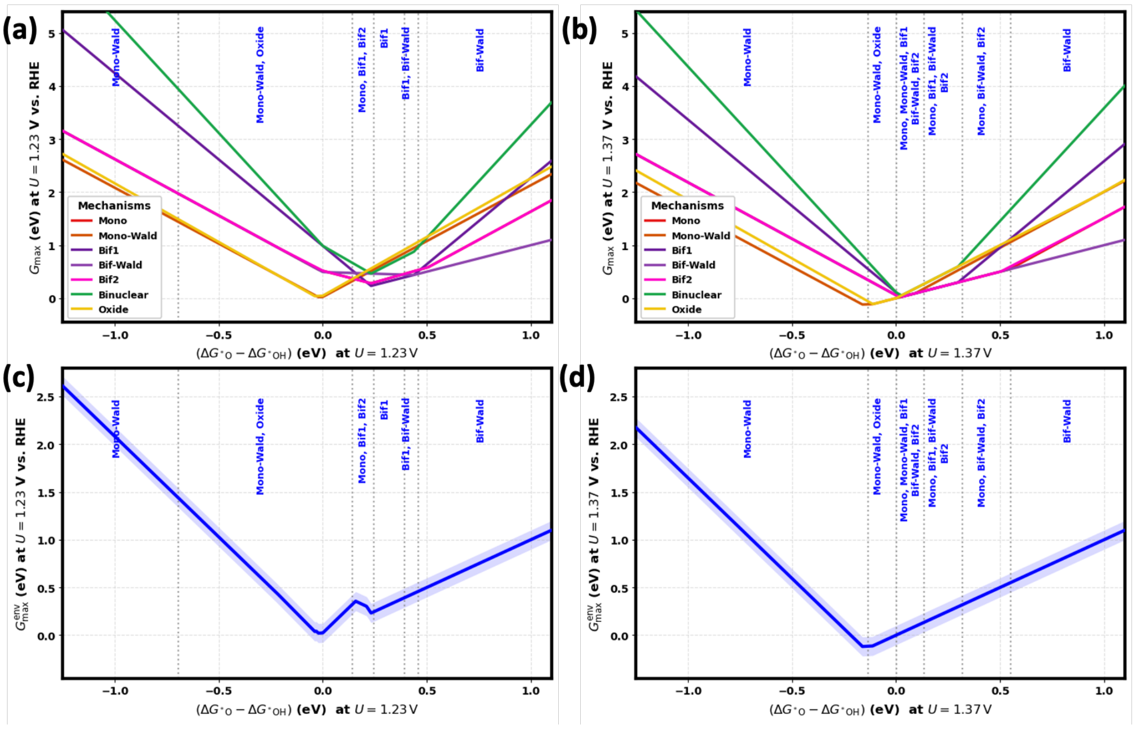

Figure S2. Procedure to determine mechanistically resolved, potential-dependent volcano plots for the oxygen evolution reaction on doped Co_3O_4 surfaces at (a, c) U = 1.23 V and (b, d) 1.37 V vs. RHE. (a, b) The scaling-dependent free energies analyzed by the $G_{max}(U)$ proxy are plotted as a function of the descriptor ($\Delta G_{*O} - \Delta G_{*OH}$) at different electrode potentials. The energetically favored mechanistic descriptions are extracted, which give rise to the envelope G_{max}^{env} (cf. equation (3)) in (c, d) for U = 1.23 V and 1.37 V vs. RHE, respectively. The shaded area indicates uncertainty of the trend line based on the error bars of the scaling relationships. The vertical dashed lines mark transitions in the energetically favored reaction mechanism, and the preferred mechanistic description is given in each $\Delta G_{*O} - \Delta G_{*OH}$ regime. Data points for the doped $Co_3O_4(001)$ surface models are added in **Figure 5** of the main text, which is based on panel (d) in this figure.

S3 Mechanistic Insights into the OER on Doped Co_3O_4(001)

While **Figure 2** in the main text summarizes the $G_{max}(U)$ values for the different pathways of all doped Co_3O_4(001)-4*OH surfaces, we show the corresponding free-energy profiles that enable deriving the $G_{max}(U)$ values in **Figures S3–S8** at U = 1.37 V vs RHE. Based on these free-energy diagrams, the dopant effects of the six different foreign transition metals are quantified in **Figure S9** by analyzing

$$\Delta G_{\max}(U) = G_{\max}^{TM,doping\ site}(U) - G_{\max}^{ref}(U) \quad \text{(S51)}$$

Note that in equation (S51), $G_{\max}^{TM,doping\ site}(U)$ and $G_{\max}^{ref}(U)$ are the $G_{max}(U)$ values for the TM-doped Co_3O_4(001) models (cf. **Figure 2** of the main text) and the undoped-Co_3O_4(001) surface by referring to the energetically preferred mechanism at U = 1.37 V vs RHE, respectively.

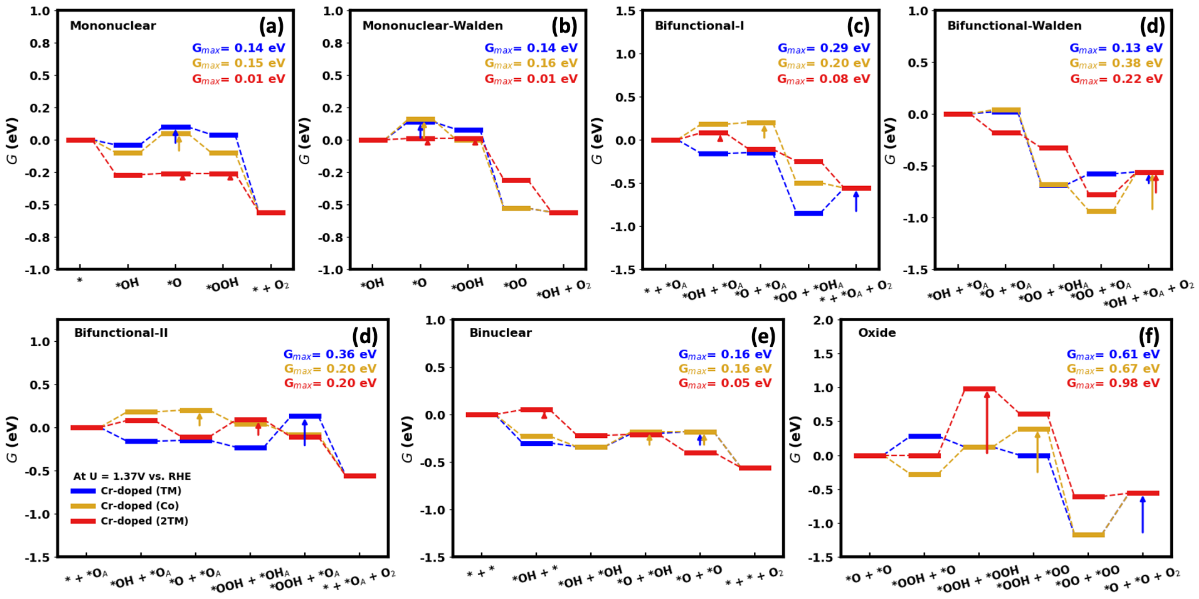

Figure S3. Free-energy diagrams of seven different reaction mechanisms for the oxygen evolution reaction (OER) on Cr-doped Co_3O_4 at U = 1.37 V vs. RHE. Panels (a–f) correspond to the mononuclear, mononuclear-Walden, bifunctional-I, bifunctional-Walden, bifunctional-II, binuclear, and oxide mechanisms, respectively. Results are shown for three different active site configurations: Cr doped at the active site (TM-site, blue), Cr doped at a neighboring Co site (Co-site, gold), and dual Cr substitution (2TM-sites, red). The free-energy span governing the $G_{\max}$ descriptor is indicated in each plot for the respective mechanism and configuration.

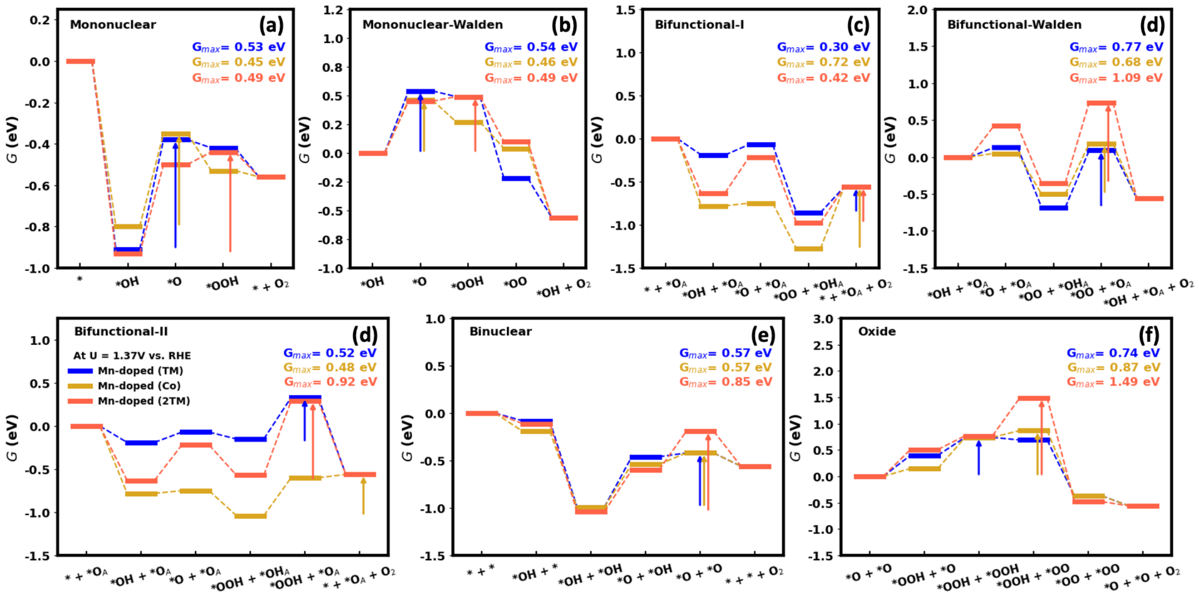

Figure S4. Free-energy diagrams of seven different reaction mechanisms for the oxygen evolution reaction (OER) on Mn-doped Co_3O_4 at U = 1.37 V vs. RHE. Panels (a–f) correspond to the mononuclear, mononuclear-Walden, bifunctional-I, bifunctional-Walden, bifunctional-II, binuclear, and oxide mechanisms, respectively. Results are shown for three different active site configurations: Mn doped at the active site (TM-site, blue), Mn doped at a neighboring Co site (Co-site, gold), and dual Mn substitution (2TM-sites, red). The free-energy span governing the $G_{\max}$ descriptor is indicated in each plot for the respective mechanism and configuration.

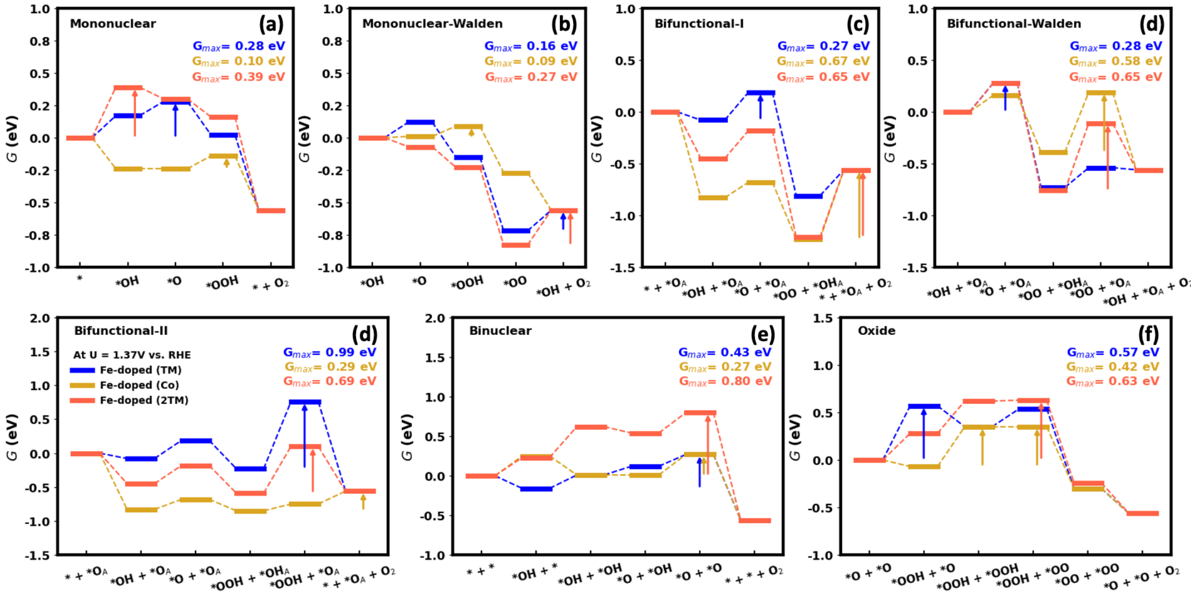

Figure S5. Free-energy diagrams of seven different reaction mechanisms for the oxygen evolution reaction (OER) on Fe-doped Co_3O_4 at U = 1.37 V vs. RHE. Panels (a–f) correspond to the mononuclear, mononuclear-Walden, bifunctional-I, bifunctional-Walden, bifunctional-II, binuclear, and oxide mechanisms, respectively. Results are shown for three different active site configurations: Fe doped at the active site (TM-site, blue), Fe doped at a neighboring Co site (Co-site, gold), and dual Fe substitution (2TM-sites, red). The free-energy span governing the G_{max} descriptor is indicated in each plot for the respective mechanism and configuration.

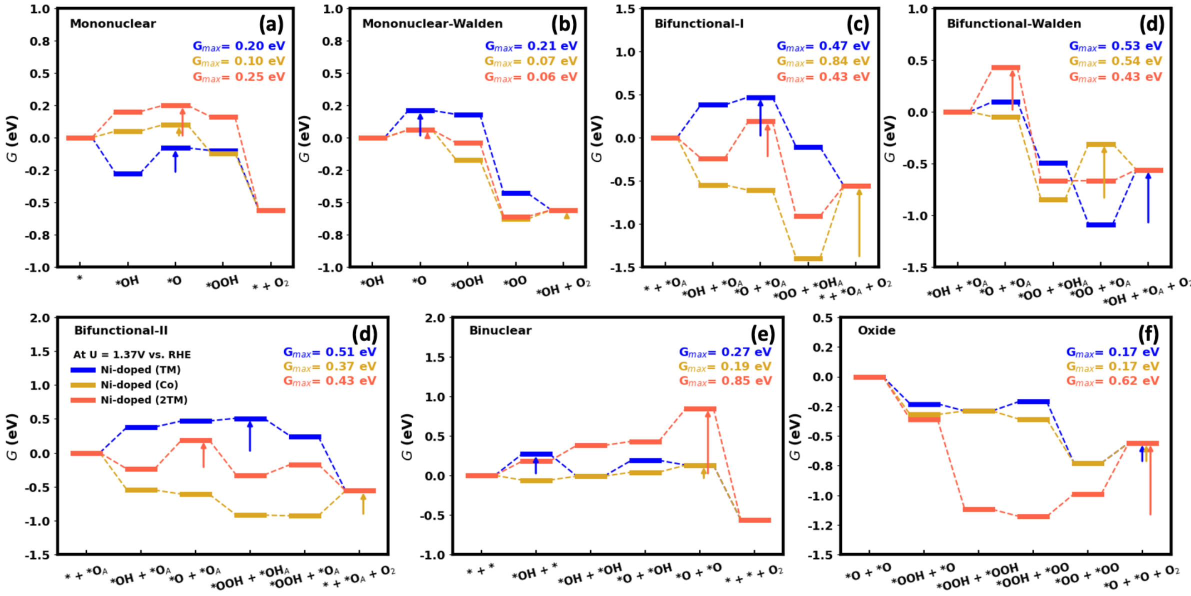

Figure S6. Free-energy diagrams of seven different reaction mechanisms for the oxygen evolution reaction (OER) on Ni-doped Co_3O_4 at U = 1.37 V vs. RHE. Panels (a–f) correspond to the mononuclear, mononuclear-Walden, bifunctional-I, bifunctional-Walden, bifunctional-II, binuclear, and oxide mechanisms, respectively. Results are shown for three different active site configurations: Ni doped at the active site (TM-site, blue), Ni doped at a neighboring Co site (Co-site, gold), and dual Ni substitution (2TM-sites, red). The free-energy span governing the G_{max} descriptor is indicated in each plot for the respective mechanism and configuration.

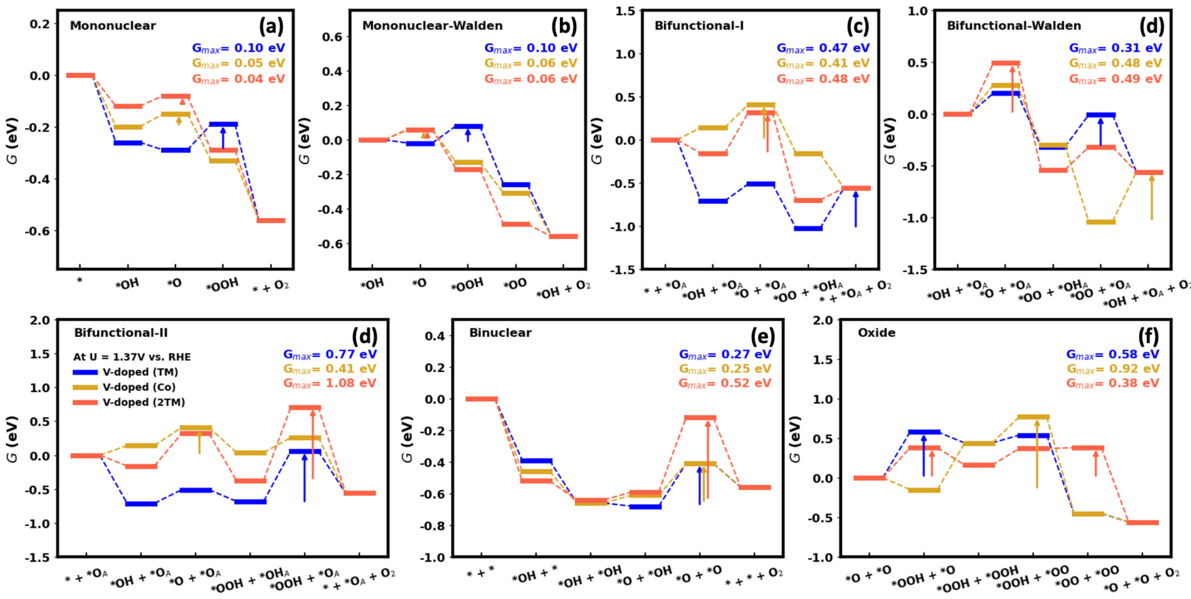

Figure S7. Free-energy diagrams of seven different reaction mechanisms for the oxygen evolution reaction (OER) on V-doped Co_3O_4 at U = 1.37 V vs. RHE. Panels (a–f) correspond to the mononuclear, mononuclear-Walden, bifunctional-I, bifunctional-Walden, bifunctional-II, binuclear, and oxide mechanisms, respectively. Results are shown for three different active site configurations: V doped at the active site (TM-site, blue), V doped at a neighboring Co site (Co-site, gold), and dual V substitution (2TM-sites, red). The free-energy span governing the G_{max} descriptor is indicated in each plot for the respective mechanism and configuration.

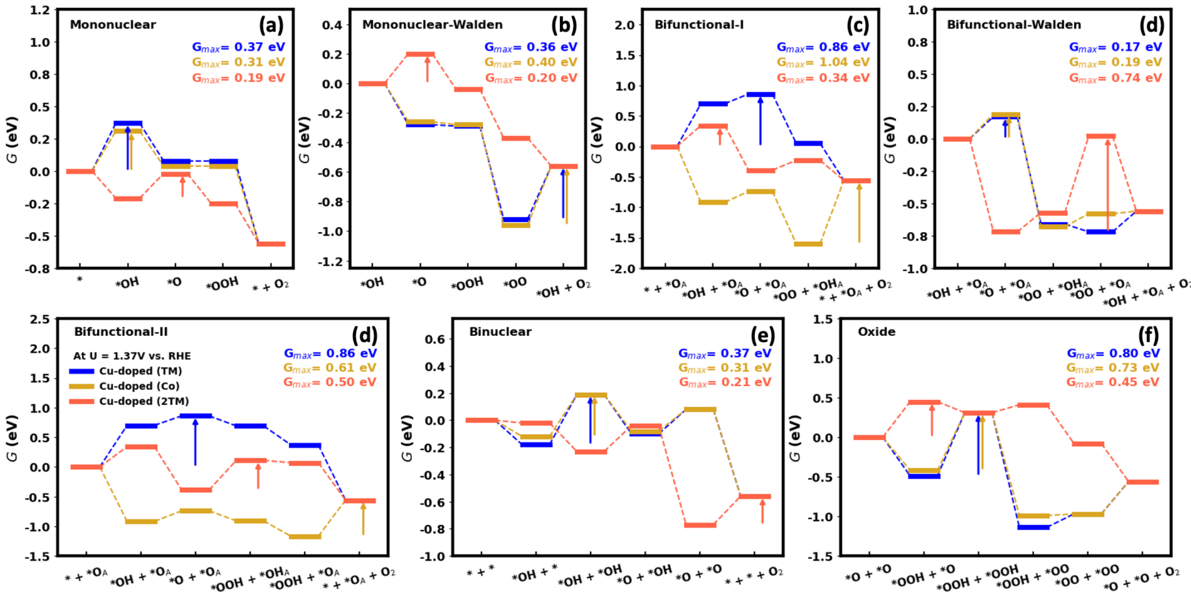

Figure S8. Free-energy diagrams of seven different reaction mechanisms for the oxygen evolution reaction (OER) on Cu-doped Co_3O_4 at U = 1.37 V vs. RHE. Panels (a–f) correspond to the mononuclear, mononuclear-Walden, bifunctional-I, bifunctional-Walden, bifunctional-II, binuclear, and oxide mechanisms, respectively. Results are shown for three different active site configurations: Cu doped at the active site (TM-site, blue), Cu doped at a neighboring Co site (Co-site, gold), and dual Cu substitution (2TM-sites, red). The free-energy span governing the G_{max} descriptor is indicated in each plot for the respective mechanism and configuration.

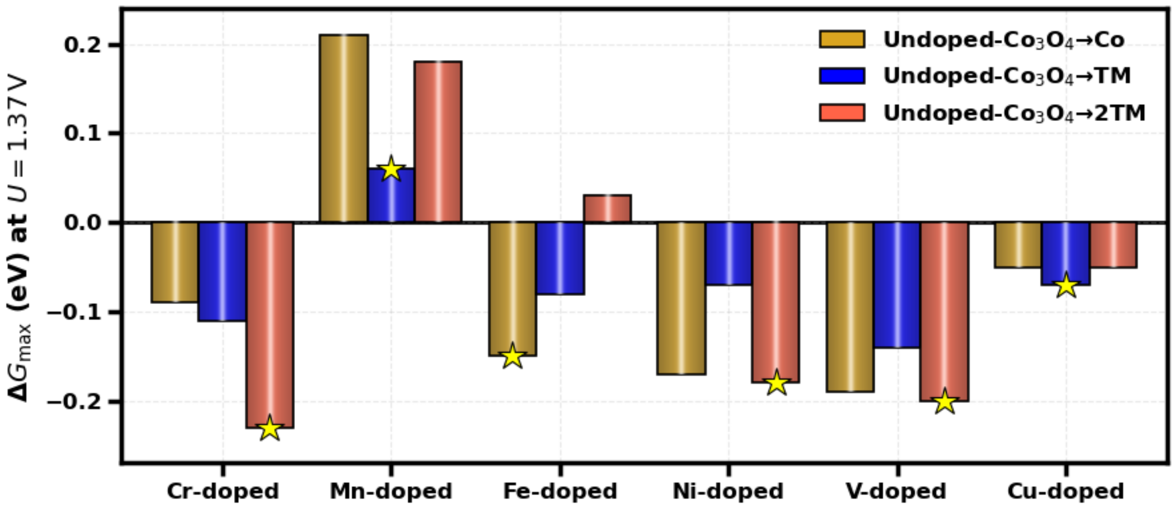

Figure S9. Changes in the calculated ΔG_{max} values (eV) at U = 1.37 V for different doped Co_3O_4 systems relative to the undoped reference. Three transitions are compared: Undoped-Co_3O_4→Co (gold), Undoped-Co_3O_4→TM (blue), and Undoped-Co_3O_4→2TM (orange). Negative ΔG_{max} values indicate an improvement in OER activity compared to the pristine surface. The yellow stars highlight the most favorable transition (largest decrease in ΔG_{max}) for each dopant.

Figure S9 clearly shows that most dopants enhance the OER activity of Co_3O_4 by decreasing the $\Delta G_{max}(U)$ value relative to the undoped system: Cr-, Fe-, Ni-, and V-doped systems display negative $\Delta G_{max}(U)$ values, indicating reaction channels with a favorable thermodynamic description of the elementary steps. Cu doping also improves the activity but only marginally compared to the other dopants. In contrast, Mn doping leads to an increase in ΔG_{max}, suggesting a loss of catalytic efficiency and highlighting its unfavorable electronic interaction with the active sites.

S4 Dual-Intermediate Scaling Relations on Doped Co_3O_4(001)

While the linear scaling relationships of the intermediate states in the OER over doped Co_3O_4(001)-4*OH surfaces are shown in **Figure 4** of the main text, **Figure S10** extends this analysis to co-adsorbed pairs on neighboring sites of Co_3O_4(001). These co-adsorbed states are particularly relevant to bifunctional and binuclear descriptions (cf. section S2.3). A definition of the co-adsorbed pairs is provided in equations (S52) – (S53). Note that these definitions are based on the application of the CHE approach for the proton-electron pair.

$$\Delta G_{*OH*OH} = G(*OH_A , *OH_B) - G(*_A , *_B) - 2\mu_{H2O} + \mu_{H2} \tag{S52}$$

$$\Delta G_{*O*O} = G(*O_A , *O_B) - G(*_A , *_B) - 2\mu_{H2O} + 2\mu_{H2} \tag{S53}$$

For these co-adsorbed pairs, we determine the slopes (a_1 and a_2) and intercepts (c_1 and c_2) according to the scaling relationships in equations (S54) – (S55):

$$\Delta G_{*OH*OH} = a_1 \, \Delta G_{*OH} + c_1 \quad \text{(S54)}$$

$$\Delta G_{*O*O} = a_2 \, \Delta G_{*OH*OH} + c_2 \quad \text{(S55)}$$

We note that the scaling correlations in **Figure S10** are still somewhat linear, although the coefficient of determination is distinctly reduced compared to the traditional scaling relations in **Figure 4** of the main text. The advantage of considering co-adsorbed states is that the application of the scaling relationships for the co-adsorbed pairs of equations (S54) – (S55) allows constructing a volcano plot without exhaustive enumeration of A-B arrangements, as performed in section S2.3 of the SI. The disadvantage of this approach is that the reduced linearity of the scaling relationships impairs the informative value of the volcano plot, as it can lead to scatter and incorrect results. We define a coefficient of determination of 0.75 as a threshold to distinguish between linear and pseudolinear correlations. Given that the ΔG_{*O*O} vs ΔG_{*OH*OH} does not meet this criterion, we rely on the scaling relations from **Figure 4** of the main text to derive the mechanistically resolved, potential-dependent volcano plot in **Figure 5**.

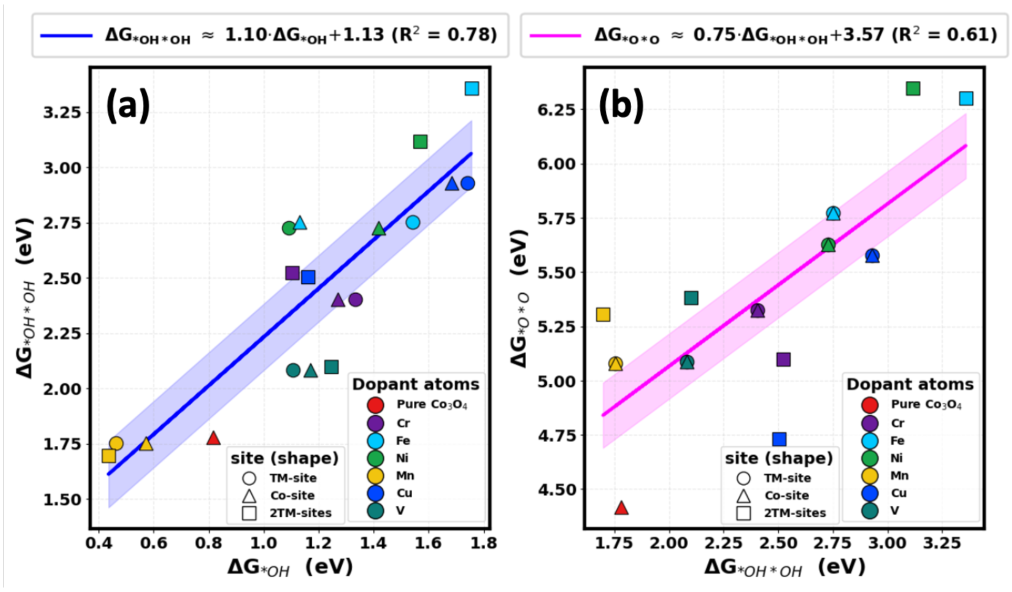

Figure S10. Extended scaling relations involving dual-intermediate descriptors for pristine and 3d TM-doped Co_3O_4. (a) Correlation between ΔG_{*OH*OH} and ΔG_{*OH}; (b) Correlation between ΔG_{*O*O} and ΔG_{*OH*OH}. Each data point corresponds to a specific dopant atom (Cr, Mn, Fe, Ni, Cu, V, or undoped Co_3O_4) and site type (TM, Co, or 2TM-sites). Regression lines are shown with shaded confidence intervals, along with the fitted slope, intercept, and coefficient of determination (R^2). These relations highlight the coupling of multi-site binding energetics that complements conventional *OH, *O, and *OOH scaling in OER catalysis.

S5 Comparison with the Conventional Volcano Approach

The conventional OER volcano plot is only based on the scaling relationship ΔG_{*OOH} vs ΔG_{*OH} by assuming a slope of unity and an offset of 3.20 eV, while the electrocatalytic activity is described by the thermodynamic overpotential, η_{TD}, which is given by equation (S56):

$$\eta_{TD} = \max(\Delta G_1, \Delta G_2, \Delta G_3, \Delta G_4) / e - 1.23 \text{ V} \quad \text{(S56)}$$

Note that the free-energy changes ΔG_j (j = 1, …, 4) only refer to the mononuclear mechanism (cf. equations (S5) – (S8)).

Figure S11 plots the thermodynamic overpotential for the doped Co_3O_4(001)-4*OH surfaces as a function of $\Delta G_2 = \Delta G_{*O} - \Delta G_{*OH}$. While on the right leg of the volcano the formation of surface oxygen limits the electrocatalytic activity (*OH → *O), the *OOH adsorbate governs the activity in the volcano on the left leg (*O → *OOH).

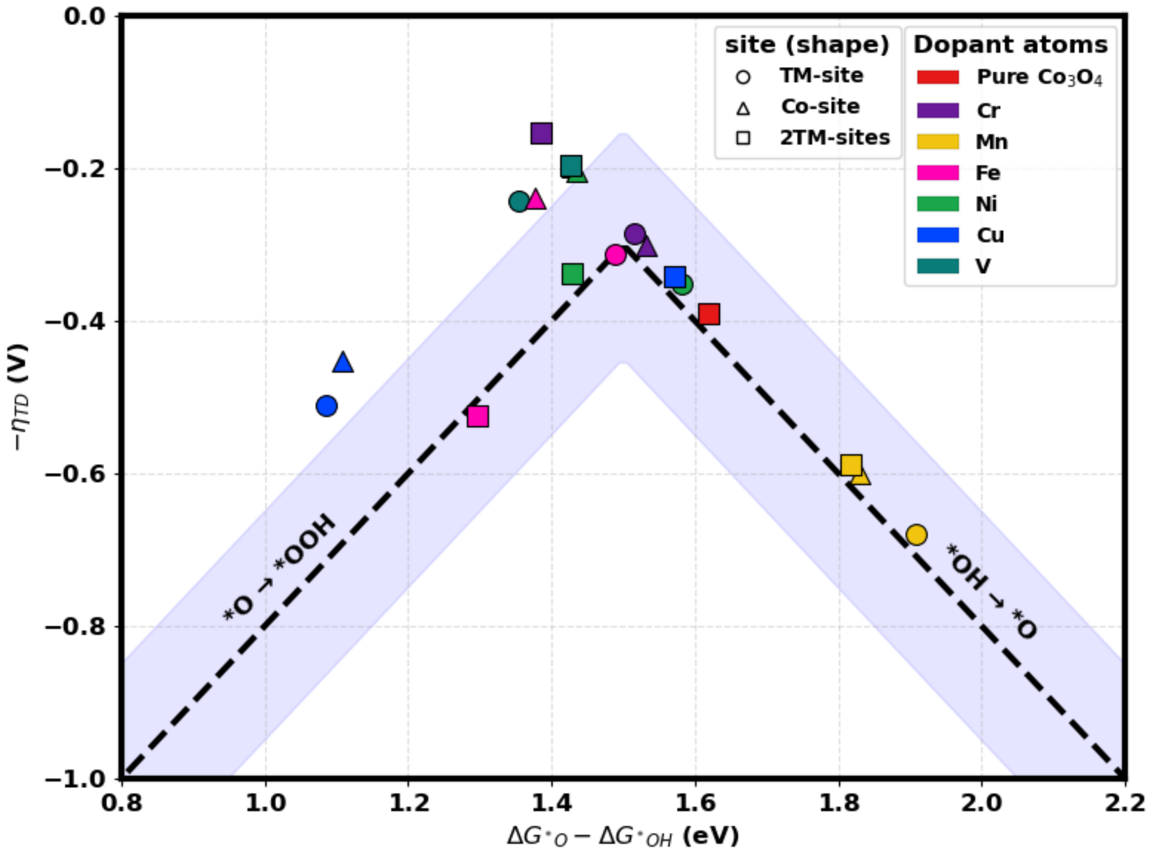

Figure S11. Conventional volcano plot for the oxygen evolution reaction (OER) over doped Co_3O_4(001) surfaces by quantifying the thermodynamic overpotential as a function of ΔG_{*O} - ΔG_{*OH} at U = 1.23 V vs RHE. The dashed line corresponds to the analytic volcano obtained by using the scaling relationship $\Delta G_{*OOH} \approx \Delta G_{*OH}$ + (3.20 ± 0.20) eV. The shaded band indicates the sensitivity range based on the error bars of this scaling relation. Colored markers and shapes denote dopant identity and site roles. Points close or above the volcano apex correspond to high OER activity, while the left and right branches of the volcano are limited by the *O→*OOH and *OH→*O steps, respectively.

The analytic volcano shown in **Figure S11** is constructed using the conventional scaling relation $\Delta G_{*OOH} \approx \Delta G_{*OH} + (3.20 \pm 0.20)$ eV, where the uncertainty in the intercept defines the shaded sensitivity band around the dashed trend line. Thus, even within the conventional framework, a limited scatter around the ideal volcano is already expected.

While the conventional volcano representation captures some qualitative activity trends, noticeable deviations from the analytic volcano line are observed for the present dataset. In particular, approximately 40% of the calculated data points lie outside the sensitivity band of the analytic volcano. In addition, several points appear above the volcano apex. Within the conventional interpretation, such behavior is often attributed to a breaking of the linear scaling relationships.

However, our analysis demonstrates that the scaling relationship between ΔG_{*OOH} and ΔG_{*OH} remains intact across the investigated dopants (cf. **Figure 4** in the main text). The apparent deviations therefore do not arise from a breakdown of the scaling relation itself, but from the limitations of the conventional activity descriptor η_{TD}. In the traditional volcano framework, η_{TD} evaluates only the largest free-energy change among the four steps of the mononuclear adsorbate evolution mechanism. As a result, this descriptor implicitly assumes a single reaction pathway and a single governing rate-limiting step.

These assumptions become restrictive for doped oxide surfaces, where several OER mechanisms may compete. Moreover, η_{TD} does not account for the explicit potential dependence of proton-coupled electron-transfer steps via the computational hydrogen electrode relation, $\Delta G(U) = \Delta G(0) - neU$. Consequently, changes in the limiting step and mechanistic crossovers with applied potential cannot be captured within the η_{TD} framework.

In contrast, the mechanistically resolved volcano plot presented in **Figure 5** of the main text is constructed using the potential-dependent envelope free-energy span $G_{max}^{env}(U = 1.37V)$. This descriptor evaluates the full free-energy profiles of several competing OER mechanisms and selects the lowest free-energy span at a given potential. Because $G_{max}^{env}(U)$ explicitly accounts for both potential dependence and mechanistic competition, it provides a more comprehensive description of catalytic activity trends.

Consequently, the broader distribution of data points observed in the conventional volcano (**Figure S11**) does not indicate broken scaling relations but instead reflects the simplified mechanistic assumptions inherent in the traditional approach. The mechanistically resolved framework introduced in this work (**Figure 5**) therefore provides a more robust interpretation of activity trends for doped Co_3O_4 catalysts under applied electrochemical bias.

S6 Experimental Details: Pulse-Optimized Samples of Surface-Doped Co_3O_4

Synthesis of spherical Co_3O_4 nanoparticles

Spherical cobalt spinel (Co_3O_4) nanoparticles were synthesized in aqueous medium using cobalt nitrate hexahydrate, ammonia, and hydrogen peroxide as the main reagents. Initially, a measured amount of $Co(NO_3)_2 \cdot 6H_2O$ was dissolved in 100 mL of deionized water under continuous stirring (500 rpm) in a 250 mL beaker. To this solution, 25 % ammonia was added dropwise via syringe, causing an immediate color change from pink to blue, indicating cobalt–ammine complex formation. Subsequently, 30 % hydrogen peroxide was added dropwise using a pipette, turning the solution black, signaling the onset of nanoparticle precipitation.

The mixture was then transferred to an oil bath and heated to 60 °C under reflux, with stirring maintained for 15 min. Separately, $Co(NO_3)_2 \cdot 6H_2O$ was dissolved in 40 mL of deionized water to prepare a second pink solution, which was added to the main reaction mixture. The combined solution was stirred at 60 °C for 3 h to complete the reaction.

After reaction completion, the black precipitate was isolated by decanting the supernatant and transferring the solid into four centrifuge tubes with deionized water. Centrifugation was carried out at 3000 rpm for 10 min per cycle. The product was washed twice with 40 mL deionized water and twice with 30 mL acetone, each wash followed by centrifugation under the same conditions. The final precipitate was dried overnight at ambient temperature and subsequently ground into a fine powder.

Laser-induced dopant incorporation into spherical Co_3O_4 nanoparticles

Preparation of colloidal dispersion

PUDEL (Pulsed Laser Doping and Excitation) treatment was performed on stable colloidal dispersions to ensure homogeneous laser–particle interaction and reproducible doping effects. The synthesized Co_3O_4 nanoparticle powder was dispersed in Milli-Q water (18.2 MΩ cm) at a concentration of 333 mg L^{-1}. Colloidal stability was enhanced by pH adjustment to 3.2 using 1 M HCl, yielding optimal dispersion properties.

Dopant addition

For the doping series, 0.5 mM of the selected transition-metal salt ($FeCl_3$, VCl_3, $NiCl_2$, $CrCl_3$) was added. Each salt is chemically stable under the mildly acidic conditions, avoiding precipitation. These conditions ensure all dopant species remain dissolved for efficient incorporation into the Co_3O_4 lattice during laser irradiation.

Laser processing

Dispersions were irradiated with a 532 nm nanosecond laser (EdgeWave IS400 slab-laser) under experimental parameters listed in Table S1.

Table S1. Laser parameter applied for PUDEL.

Description	Value
Pulse length [ns]	7
Wavelength [nm]	532
Repetition rate [kHz]	4-8
Knife distance [mm]	1.3
Flat jet thickness [µm]	39.12
Flat jet height [mm]	1.7
Flow rate [mL/min]	20
Flat spry nozzle angle [°]	80

Suspensions were stirred at 360 rpm in a 400 mL beaker during irradiation to prevent sedimentation. For the doping experiments, a fixed intensity of 0.5×10^{11} W m^{-2} was used, to avoid CoO formation, which was shown to appear if energies exceed 0.5×10^{11} W m^{-2}.[36,37]

PPV was varied by adjusting repetition rate at constant volume flow as followed: 1 PPV at 4 kHz; 2 PPV at 8 kHz; 3 PPV by 4 kHz followed by 8 kHz irradiation. This protocol enabled systematic study of cumulative laser energy effects on doping efficiency and nanoparticle physicochemical properties.

A scheme of the experimental setup is presented in previous study.[38]

Recovery and purification

For undoped samples, pH was raised to 7.3 using 1:16 diluted ammonia, destabilizing the colloid and inducing sedimentation over 2 h. Supernatant was decanted, and the concentrated suspension frozen and freeze-dried (ALPHA 1-4 LDplus, Christ; 0.5 mbar, −56 °C) for gentle water removal without altering nanoparticle structure or surface chemistry.

For doped samples, pH-induced precipitation was avoided to prevent dopant hydroxide/oxide contamination. Recovery was performed by centrifugation at 15 000 rpm for 10 min, followed by redispersion in fresh Milli-Q water. Three washing cycles (centrifugation + redispersion) removed residual metal ions and chloride. Freeze-drying was carried out under the same conditions as above.

Characterization of the nanoparticles after laser-treatment

Dopant concentration analysis by X-ray fluorescence spectroscopy

Dopant concentrations were determined by X-ray fluorescence spectroscopy (XRF) using a Bruker S8 TIGER wavelength dispersive X-ray fluorescence (WDXRF) spectrometer. The instrument was calibrated with certified reference materials (CRMs) of known elemental concentrations. X-ray line intensities from these standards were correlated with their certified values to generate calibration curves, typically using linear or polynomial regression. To minimize matrix effects, standards with compositions closely matching the samples were selected. This empirical calibration approach ensures accuracy, reproducibility, and traceability of quantitative results within the certified ranges.

For each measurement, 5 mg of powder was dispersed in 8 mL of Milli-Q water (18.2 MΩ cm) to obtain a concentration of 625 mg L^{-1}, and the pH was adjusted to 3 to ensure long-term colloidal stability. The measured dopant concentrations are listed in Table S2. Nickel content could not be quantified because its concentration was below the detection limit of the method (compare Figure S12)

Table S2. Mean dopant (D) concentrations (cation-based concentration) and the corresponding standard deviation determined by XRF after laser treatment of three consecutive measurements.

Dopant	$x_{D/(D+Co)}$*100%	Standard deviation
Cr	0.16334	0.01814
Fe	3.65885	0.2173
Ni	Below detection limit	-
V	2.76607	0.55465

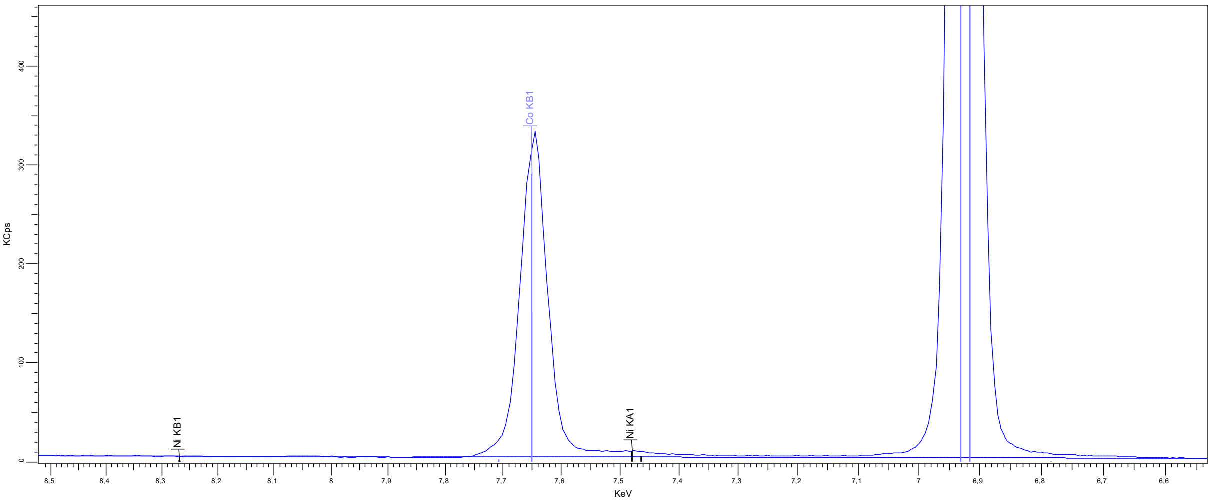

Figure S12. XRF spectrum of the nickel-doped sample. A small shoulder in the baseline region corresponds to the Ni Kα emission line, confirming nickel presence; however, its low intensity prevents quantitative determination of Ni content. The Figure represents a screenshot of the recorded spectrum.

Particle size measurement by analytical disc centrifuge (ADC)

Measurements were performed using a disc centrifuge (model DC 24000, CPS Instruments, Inc.) starting from a nanoparticle dispersion in Milli-Q water at a concentration of 250 mg L^{-1}. The initial pH was adjusted to 3 with 0.1 M HCl. Before each measurement, the dispersion was sonicated for 15 min to ensure good particle dispersion and to overcome weak interparticle forces that cause aggregation.

At the start of the measurements, a density gradient was introduced into the instrument and refreshed after every five measurements. This gradient was prepared from two sugar solutions (8 % and 24 % w/w), each diluted with Milli-Q water. Dodecane was applied separately as an overlay or sealing fluid.

Prior to each sample injection, a calibration standard of PVC particles (0.544 µm in distilled water; CPS Instruments, Inc.) was injected to validate the instrument setup. All measurements were performed down to a minimum detectable particle size of 13 nm, with an acquisition time of 15 min.

Figure S13 presents the mean particle diameter following laser treatment. A slight decrease in size compared to untreated Co_3O_4 is observed, likely resulting from surface heating during irradiation.[39] Multiple pulses could promote incremental sintering, producing marginal size reductions. However, even with the largest observed deviation (~4 nm), particle sizes remain within the same overall distribution range, indicating that morphological change can be neglected.

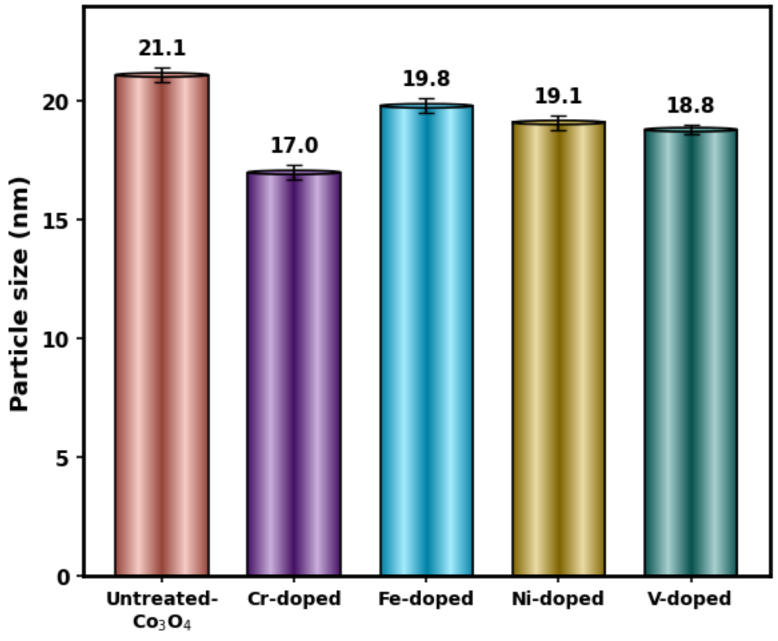

Figure S13. Mean particle size after laser treatment: 3 pulses per volume (PPV) for Cr, Ni, and V, and 2 PPV for Fe compared to the untreated Co_3O_4 sample.

Catalytic performance of the nanoparticles after laser-treatment

Electrochemistry measurements

Electrochemical investigations were performed using a three-electrode setup connected to a potentiostat (Parstat 4000A, Princeton Applied Research, AMETEK, Berwyn, USA). The setup consisted of a leak-free Ag/AgCl reference electrode (3 M KCl internal solution), a platinum wire counter electrode, and a glassy carbon electrode (GCE) as the working electrode. Measurements were conducted in 1 M Fe-free KOH electrolyte with a pH ≈ 14. Prior to each experiment, the electrolyte was purged with high-purity nitrogen for 15 min to remove dissolved oxygen.

Potentials measured versus Ag/AgCl were converted to the reversible hydrogen electrode (RHE) scale using following Equation:

$$E_{RHE} = E_{Ag/AgCl} + 0.059 \times pH + E^{o}_{Ag/AgCl} \qquad \text{(S57)}$$

Where:

E_{RHE} is the potential vs. RHE.

$E_{Ag/AgCl}$ is the measured potential vs. Ag/AgCl.

pH is the electrolyte pH (≈ 14 for 1 M KOH).

$E^{o}_{Ag/AgCl}$ is the standard potential of the Ag/AgCl electrode (0.202 V at 25 °C in 3 M KCl).

Catalyst Ink Preparation and Deposition

For preparation of the catalyst ink, 2 mg of Co_3O_4 were dispersed in 0.4 mL of a 3:1 (v/v) water/isopropanol mixture, along with 20 µL of 5 wt% Nafion solution. The suspension was ultrasonicated for 15 min to ensure homogeneity. Subsequently, 5.26 µL of the ink was drop-cast onto a glassy carbon electrode with a geometric area of 0.1963 cm², followed by drying in an oven at 70 °C for 5 min, yielding a mass loading of around 0.1276 mg cm^{-2}.

Before and immediately after each electrochemical measurement, the electrolyte pH was recorded to monitor any degradation, and the electrode surface was purged with nitrogen gas to remove residual oxygen bubbles.

Electrochemical Protocols

The applied measurement sequence is summarized in **Table S3**. This standardized procedure was used to determine electrolyte resistance, charge-transfer resistance, and catalytic performance by linear sweep voltammetry (LSV).

Table S3. Details of the electrochemical measurement protocol.

**Protocol No.**	**Technique**	**Purpose**	**Parameters**
**1**	EIS	Initial pre-treatment	- Potential: 0.023 V vs RHE<br>- Frequency: 100000 → 0.1 Hz<br>- Amplitude: 5 mV RMS
**2**	CV	Conditioning	- Cycles: 20<br>- Scan rate: 50 mV/s<br>- Range: 0.023 – 0.572 V
**3**	LSV	Performance test	- Potential range: 1.059 V a 1.759 V vs RHE<br>- Scan rate: 5 mV/s
**4**	EIS	Post-performance	- Potential: 0.023 V vs RHE<br>- Frequency: 100000 → 0.1 Hz<br>- Amplitude: 5 mV RMS

PPV Series

For the PPV series, multiple measurements were performed to assess reproducibility and estimate experimental error. A fresh catalyst ink deposition was prepared for each measurement. Prior to each deposition, the ink was redispersed in an ultrasonic bath for 15 min to prevent sedimentation and ensure uniform dispersion.

Standard deviation estimation

The standard deviation of the electrocatalytic experiments was determined from four consecutive measurements using undoped Co_3O_4. For each measurement, a fresh catalyst layer was prepared by drop-casting the catalyst ink onto the glassy carbon electrode. The numerical results are summarized in **Table S4**. The box plot in **Figure S14** visualizes the reproducibility of the electrocatalytic oxygen evolution reaction (OER) experiments.

Table S4. Results of four consecutive experiments using undoped Co_3O_4 in OER reaction as described above.

Sample	Repetition	Current [mA cm^{-2}]
Co_3O_4	1	20.884

Co_3O_4	2	18.855
Co_3O_4	3	21.097
Co_3O_4	4	23.164

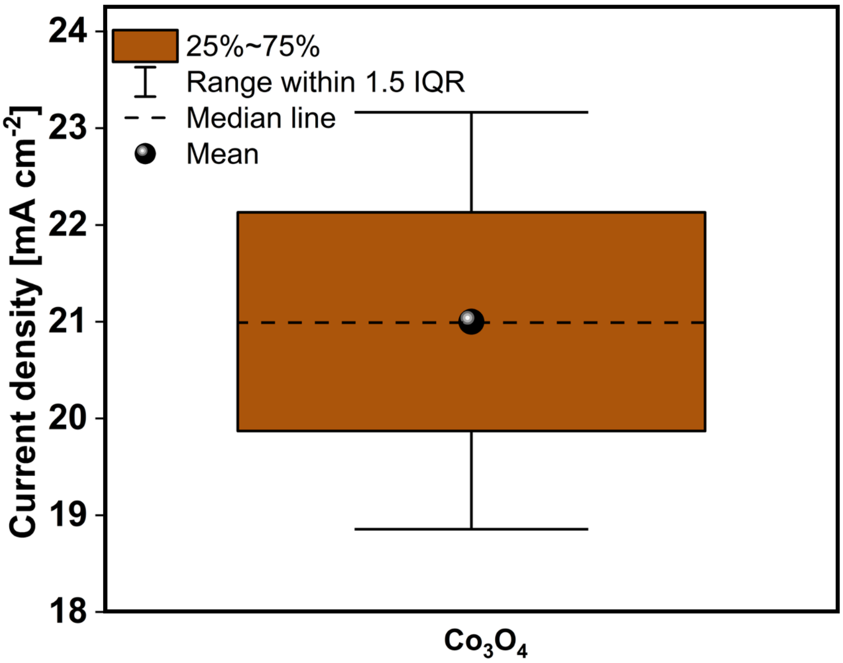

Figure S14. BOX-Plot of the reproduced electrocatalytic experiments. The standard deviation of the current density was determined to be 1.52 mA cm^{-2}.

S7 Experimental Details: Surface-Enriched, Cr-Doped Co_3O_4 nanocubes

Nanocubes Synthesis and Characterization: Co_3O_4 and $Cr_{0.1}Co_{2.9}O_4$ nanocubes were synthesized using a hydrothermal method based on metal nitrate precursors. [40] For the Cr-doped samples, a mixture of 2.9 equivalents of $Co(NO_3)_2$ and 0.1 equivalents of $Cr(NO_3)_3 \cdot 9 H_2O$, corresponding to a relative dopant concentration of 3.3 at% in the resulting particles, was used. This method avoids the use of organic capping agents. To prepare the calcined $Cr_{0.1}Co_{2.9}O_4$ sample, the pristine $Cr_{0.1}Co_{2.9}O_4$ powder was heated at 400 °C for 2 h. The particles were thoroughly characterized using SEM, TEM, XRD, and XPS. SEM and EDX were conducted using a JEOL JSM-IT700HR SEM equipped with an EDX detector (JEOL EX-74212U4L2Q), while TEM was performed on JEOL 2200FS TEM.

Electrochemical measurements were performed in a three-electrode arrangement using glassy carbon rotating disk electrode (RDE) and Autolab potentiostat. A platinum sheet served as the

counter electrode, Ag/AgCl (3 M KCl) as the reference electrode and catalyst-modified RDE as the working electrode. The electrolyte was 1.0 M KOH (Sigma-Aldrich, ≥ 99.99% trace metal basis). The potential is reported against the reversible hydrogen electrode (RHE). Typically, the electrodes were initially activated by several repeated CVs before recording the LSV. Further details and data can be found in our recent preprint.[40] The synthesized particles show well-defined shape of cubes and kept their size (ca. 250 nm) and shape after doping and calcination, as shown in **Figure S15**.

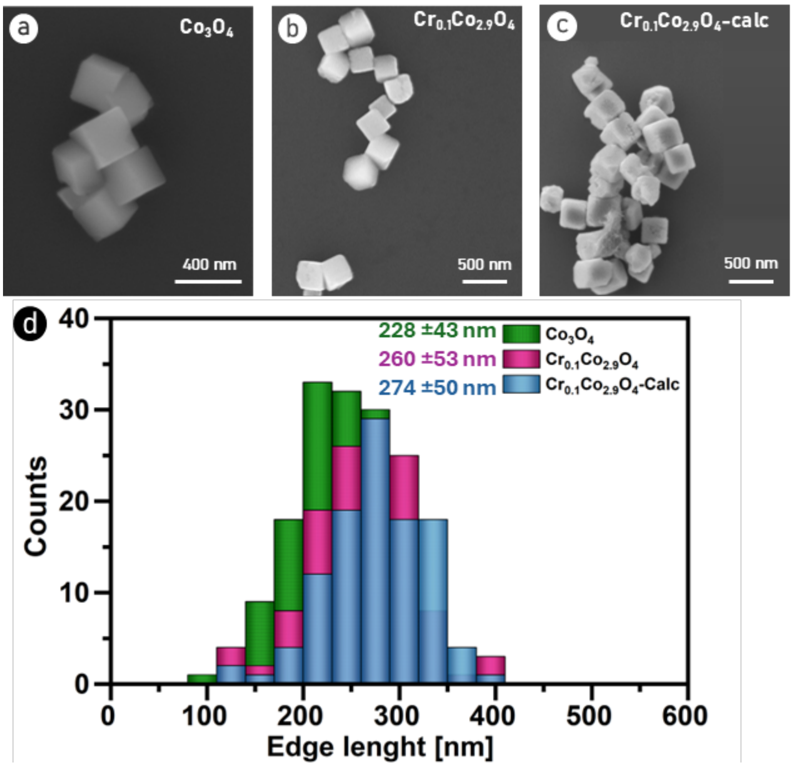

Figure S15. SEM images of (a) Co_3O_4, (b) $Cr_{0.1}Co_{2.9}O_4$ and (c) $Cr_{0.1}Co_{2.9}O_4$-calcined samples, showing that the three samples have the same well-defined cubic shape. Images are reprinted from reference.[40] (d) Particle size distribution histogram of the three samples, showing comparable particle size of about 250 nm.

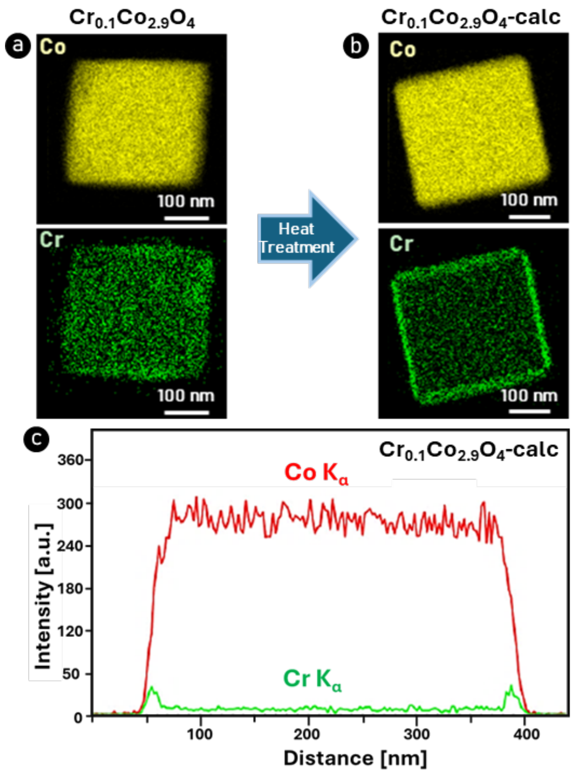

Figure S16. TEM-EDX elemental mapping of Co and Cr elements for (a) $Cr_{0.1}Co_{2.9}O_4$ and (b) $Cr_{0.1}Co_{2.9}O_4$-calcined samples. Images are reprinted from reference[40] (c) TEM-EDX line scan of the $Cr_{0.1}Co_{2.9}O_4$-calcined sample.

For the pristine $Cr_{0.1}Co_{2.9}O_4$ sample, it clearly shows cubic shape, and Cr is apparently more homogeneously distributed than the calcined $Cr_{0.1}Co_{2.9}O_4$ sample. The TEM-EDX line scan of Cr clearly shows Cr-enrichment at the cube surface compared to the bulk for the $Cr_{0.1}Co_{2.9}O_4$-calcined sample. Notably, this Cr segregation from the bulk to the surface during calcination leads to the formation of a core-shell architecture with a Cr-enriched shell of ca. 10-20 nm thickness, as shown in the TEM mapping and line scan. We note that Cr doping results in a phase segregation, which leads to the formation of a partially Cr-doped Co_3O_4 and a Cr-rich surface oxide phase, as evidenced from XPS and EDX data.

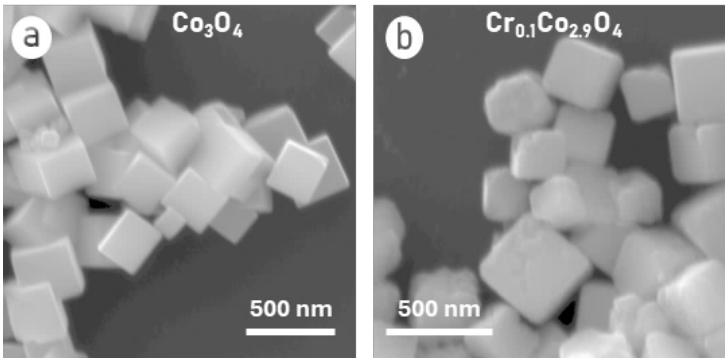

Figure S17. Post-catalysis SEM images of (a) Co_3O_4 and (b) $Cr_{0.1}Co_{2.9}O_4$ nanocubes after OER experiment.

Furthermore, SEM was performed after OER experiment to investigate possible morphological changes. **Figure S17** shows no obvious changes in the cubic shape or particle size after OER compared to their initial state.

S8 Structural analysis

S8.1 Thermodynamic Stability of the 4*OH Surface Termination

The O/OH surface termination used in the mechanistic analysis was selected based on thermodynamic surface phase stability considerations rather than as an arbitrary model assumption. Previous work reported a surface Pourbaix analysis of the $Co_3O_4(001)$ interface under electrochemical conditions, in which the relative surface free energies of several terminations were evaluated as a function of the applied electrode potential.[30,31]

As shown in **Figure S18** ,[31] the stability of different surface configurations, including the bare surface and hydroxylated terminations, was analyzed over the relevant potential range. At the thermodynamic oxygen evolution reaction potential (1.23 V vs RHE) and within the experimentally relevant anodic potential window, the 4*OH-covered $Co_3O_4(001)$ surface is predicted to be the thermodynamically most stable configuration. In contrast, the bare surface becomes unstable under these oxidizing conditions, while more strongly hydroxylated or water-covered states only become competitive at potentials outside the primary OER operating window.

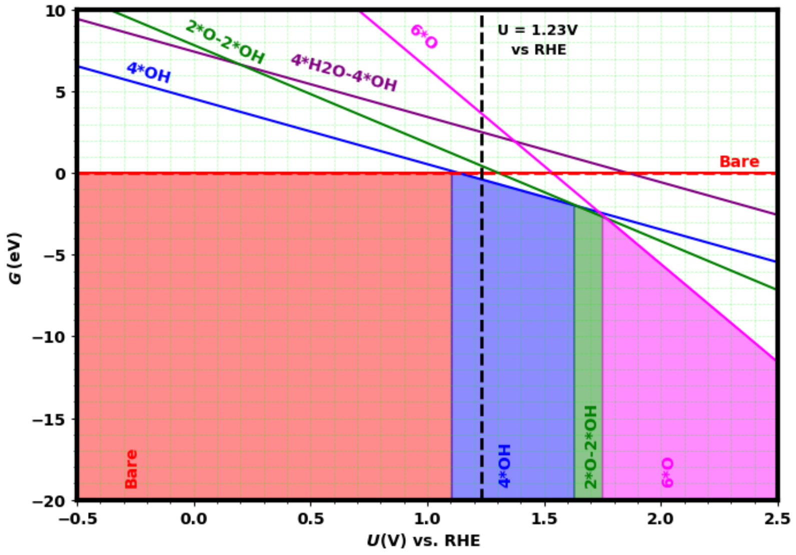

Figure S18. Free-energy diagram of surface phase stability (Pourbaix analysis) for $Co_3O_4(001)$ as a function of applied potential (*U* vs RHE). The relative stability of the bare surface, with 4*OH with other terminations is shown. The dashed vertical line indicates the thermodynamic OER potential (1.23 V vs. RHE). In the relevant anodic potential window, the 4*OH termination is thermodynamically most stable, justifying its use as the reference surface state for mechanistic analysis. Figure reproduced from reference.[31]

Based on this thermodynamic phase stability analysis, the 4*OH termination represents the most realistic surface state under OER conditions. For this reason, the Co_3O_4(001)-4*OH model was used as the reference surface structure for the mechanistic calculations presented in this work.

S8.2 Doping concentration

To evaluate whether the relatively high surface dopant concentration in the 2×2 unit cell artificially influences the predicted activity, we explicitly examined the lateral size effect by expanding the slab to a 4×4 supercell. This reduces the surface dopant concentration from 25% to 6.25%, thereby significantly weakening possible dopant–dopant interactions and better approximating a dilute experimental regime. The structural construction of the 4×4 supercell is illustrated in **Figure S19b**, and the corresponding OER free-energy diagrams for the mononuclear mechanism are shown in **Figure S20**. We deliberately restricted this analysis to a single mechanism in order to isolate the impact on adsorption energetics. The calculated maximum free-energy span (G_{max}) changes only marginally upon increasing the cell size, remaining within a narrow range. This demonstrates that the predicted Cr enhancement is not an artifact of finite-size effects or unrealistically high dopant density, and that the activity trend is preserved under more dilute surface conditions.

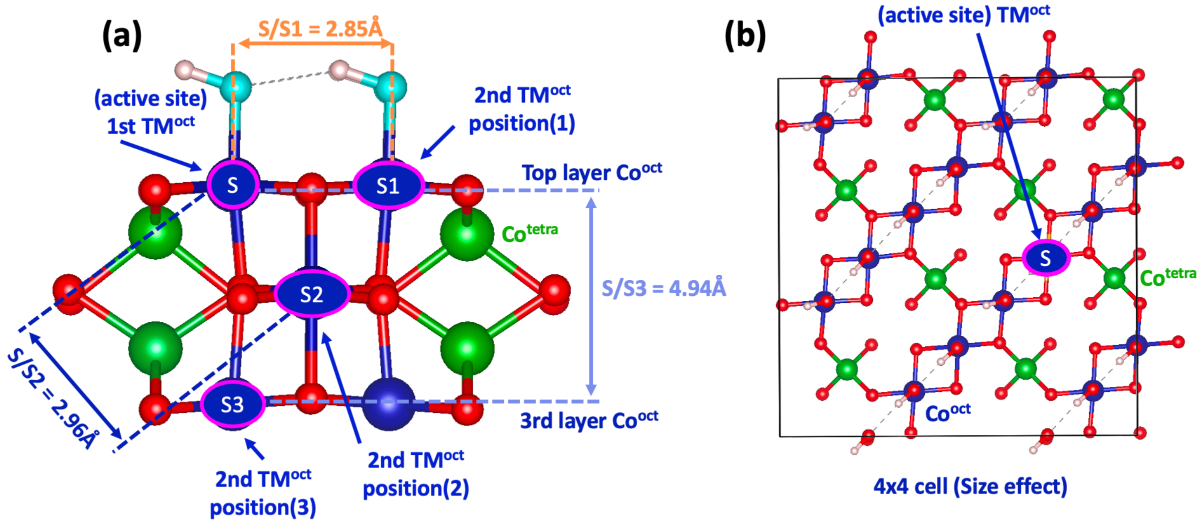

Figure S19. Structural models used to evaluate dopant position and size effects on Co_3O_4(001). (a) Schematic representation of substitution at distinct Co^{oct} sites within the slab, illustrating 1st-layer (surface), 2nd-layer, and 3rd-layer doping positions. The labeled S, S1, S2, and S3 sites indicate the active and subsurface octahedral Co positions considered for depth-dependent substitution. Characteristic intersite distances are indicated to highlight the geometric relationship between neighboring octahedral sites. (b) 4x4 surface supercell used to assess lateral size effects and reduced dopant concentration. The highlighted site denotes the substituted Co^{oct} position within the expanded cell, corresponding to a decrease in surface dopant concentration from 25% (2x2 unit cell) to 6.25% (4x4 supercell).

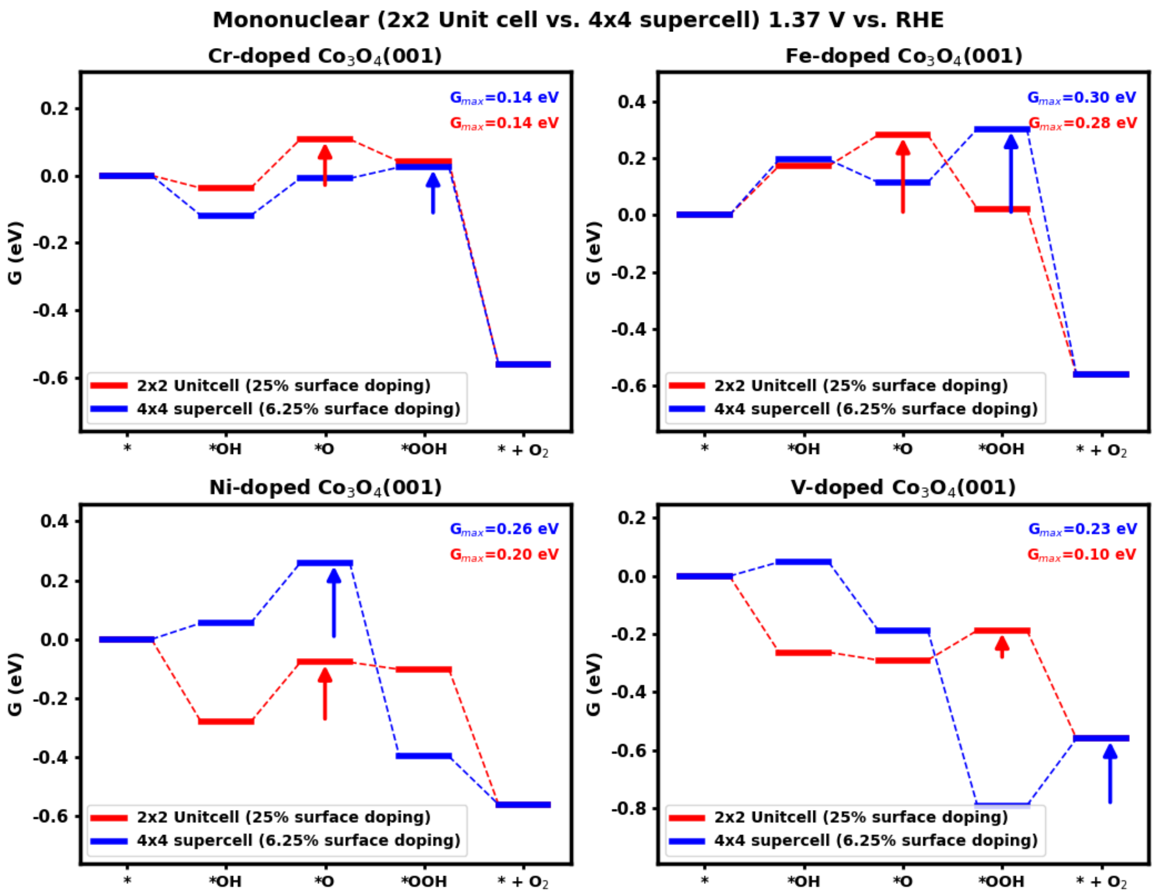

Figure S20. Mononuclear OER free-energy diagrams at U = 1.37 V vs. RHE for TM-doped Co_3O_4(001) comparing 2x2 unit cell and 4x4 supercell models. Results are shown for Cr-, Fe-, Ni-, and V-doped systems. The calculated maximum free-energy span (G_{max}) is indicated in each panel. The small variation in G_{max} upon increasing the cell size demonstrates that the predicted activity trends are robust against finite-size effects and reduced dopant concentration.

S8.3 Depth of doping

To further address uncertainties regarding the position of the dopant within the lattice, we systematically substituted octahedral cobalt atoms in three different layers of the slab, namely the first (surface), second (subsurface), and third layers, as illustrated schematically in **Figure S19a**. This analysis allows us to determine whether the catalytic activity depends sensitively on the precise depth of the dopant within the near-surface region.

The corresponding free-energy diagrams for the mononuclear OER mechanism are presented in **Figure S21**. The calculated G_{max} at U = 1.37V vs RHE vary only slightly among the three substitution depths. Importantly, the relative activity trend among the investigated systems remains unchanged regardless of whether the dopant occupies the first, second, or third octahedral layer.

These results indicate that the catalytic enhancement induced by transition-metal substitution does not critically depend on the exact vertical position of the dopant within the near-surface region. Consequently, even if experimental synthesis leads to partial subsurface incorporation of dopants, the predicted mechanistic trends and activity ranking remain preserved. This analysis therefore confirms that the conclusions of the present work are robust with respect to both lateral cell size and dopant depth within the Co_3O_4 lattice.

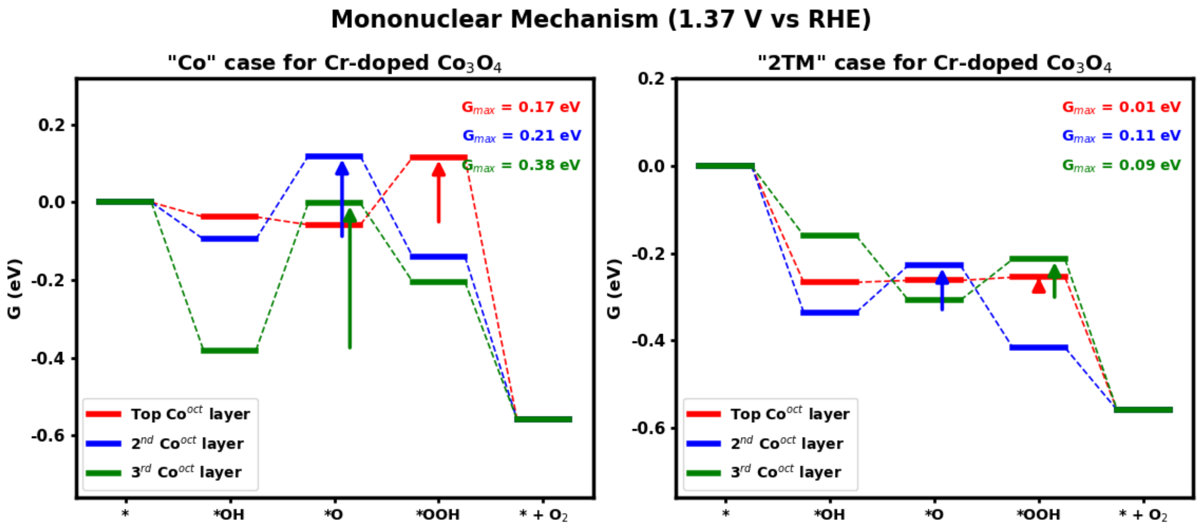

Figure S21. Mononuclear OER free-energy diagrams at U = 1.37 V vs. RHE for Cr-doped Co_3O_4(001) with dopant substitution at different octahedral depths. The red, blue, and green curves correspond to substitution at the 1st (surface), 2nd, and 3rd Co^{oct} layers, respectively. Results are shown for both the "Co" case and the "2TM" case ("TM" case is not relevant here). The calculated maximum free-energy span (G_{max}) is indicated in each panel. The limited variation in G_{max} across the three substitution depths demonstrates that the predicted activity trend is largely insensitive to dopant position within the near-surface region.

S8.4 Surface reconstruction

Understanding the surface transformation of Co_3O_4 under OER conditions is an important aspect to consider when comparing theoretical models with experimental catalysts. In this context, it is important to distinguish between different terminations of the Co_3O_4(001) surface.

Previous theoretical work by Kox et al.[41] has shown that the termination-A surface of Co_3O_4(001) tends to reconstruct under oxidizing conditions, whereas termination-B remains structurally stable. In particular, ab initio molecular dynamics (AIMD) simulations under anodic polarization conditions demonstrate that the B-termination does not undergo significant structural rearrangement, maintaining its overall lattice framework throughout the simulation time scale. This observation provides strong theoretical support for using non-reconstructed Co_3O_4(001) B-terminated surface models as a representative starting point for mechanistic studies of the OER.

Starting from this experimentally and theoretically supported surface model, we further note that adsorbate-induced local reconstructions may still occur during OER. In our previous work,[31] we systematically investigated this aspect and identified seven distinct adsorbate-covered surface motifs that arise from different combinations of O/OH and H_2O/OH coverages. These motifs represent local rearrangements of the surface coordination environment rather than large-scale lattice reconstruction. For clarity, these reconstructed configurations and their corresponding active-site motifs are reproduced (**Figure S22**).

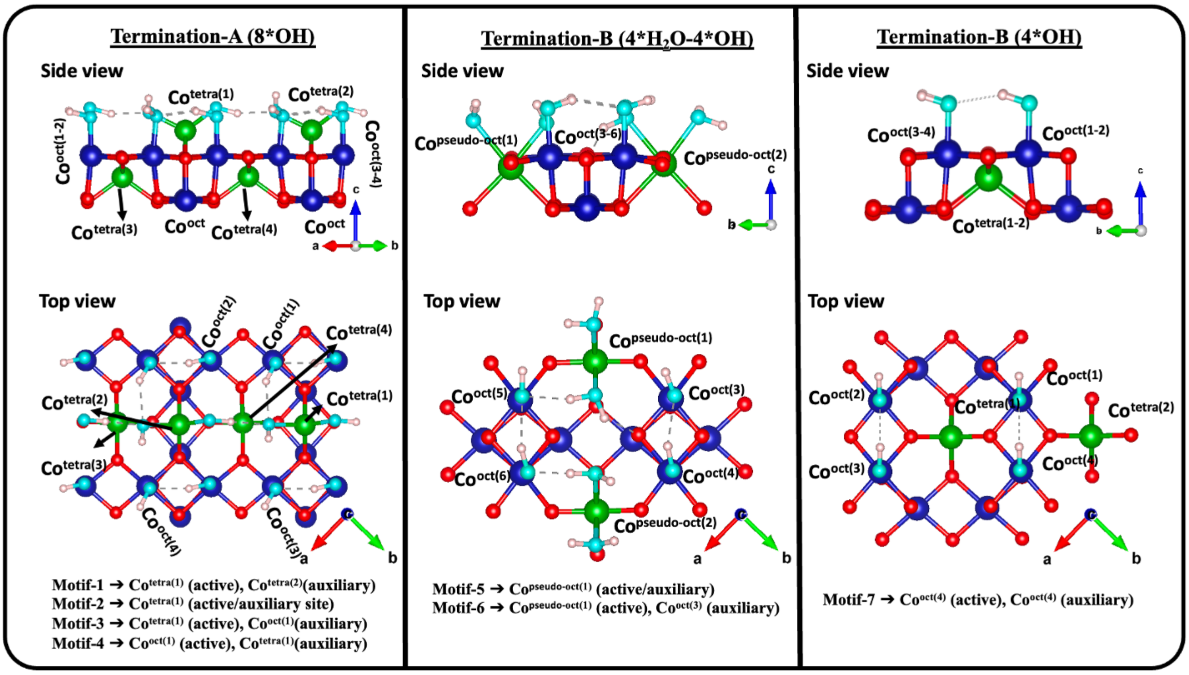

Figure S22. Schematic representation of various surface configurations of Co_3O_4(001), including termination-A (8*OH coverage) and termination-B (4*H_2O-4*OH and 4*OH coverages). All seven structural motifs considered for OER are illustrated. Blue and red spheres denote Co atoms in octahedral coordination and lattice oxygen atoms, respectively. Green spheres indicate tetrahedral Co atoms, including those that transition to pseudo-octahedral coordination, while cyan spheres represent surface-adsorbed oxygen atoms. White color represents hydrogen atoms. Please note that the atom sizes do not scale. Figure reproduced from reference.[31]

To further illustrate the structural stability of the Co_3O_4(001) surface under aqueous conditions, which shows representative snapshots and structural analysis from AIMD simulations reported in the same study.[31] These simulations illustrate that the B-termination largely preserves its lattice framework in contact with water, whereas the A-termination exhibits stronger water dissociation and a more dynamic interfacial environment.

In the present work, considering six dopants and multiple reaction mechanisms, evaluating all seven motifs for every configuration would be computationally prohibitive. Therefore, we selected motif-7, which was identified in our previous study as one of the most stable and catalytically relevant motifs under OER conditions, and used it as the primary structural model.

However, the additionally performed reconstruction calculations for selected dopants (Cr, Fe, Ni, and V). Specifically, we evaluated the mononuclear mechanism on the reconstructed motifs most relevant to this pathway, namely motif-1 and motif-5, in addition to the investigated motif-7. The resulting free-energy diagrams and corresponding maximum free-energy spans (G_{max}) are shown in **Figures S23** and **S24**.

A closer inspection of the G_{max} values shown in **Figure S23** and **S24** further clarifies the effect of surface reconstruction on the calculated activity trends. For Cr- and Fe-doped Co_3O_4, the reconstructed motifs (motif-1 and motif-5) exhibit activity trends that are comparable to the non-reconstructed motif (motif-7). In particular, motif-1 (reconstructed) confirms the trend observed for motif-7 in the main manuscript, namely that Cr substitution yields the most favorable free-energy span within the Co_3O_4 host lattice. This agreement between reconstructed and non-reconstructed motifs demonstrates that the predicted activity enhancement for Cr-doped Co_3O_4 is not an artifact of the specific surface motif considered. In contrast, for Ni- and V-doped Co_3O_4 the reconstructed motifs display somewhat higher activity than the non-reconstructed motif. Despite these differences, a consistent observation emerges when all motifs are compared with undoped Co_3O_4: independent of whether reconstructed (motif-1 or motif-5) or non-reconstructed (motif-7) configurations are considered, all investigated dopants (Cr, Fe, Ni, and V) exhibit higher catalytic activity than pristine Co_3O_4.

Finally, we emphasize that the reconstructed surface motifs (motif-1 and motif-5) together with the stable B-terminated motif-7 represent the most realistic atomistic description of Co_3O_4-based OER catalysts currently accessible within electronic-structure approaches. In particular, the combination of surface phase stability analysis, reconstructed adsorbate-covered motifs, and explicit solvation simulations such as ab initio molecular dynamics provides a consistent framework to describe Co_3O_4 surfaces under anodic polarization. Within this framework, these motifs capture the relevant local coordination environments of surface Co sites and qualitatively resemble the experimentally observed structural evolution of cobalt oxide surfaces under OER conditions.

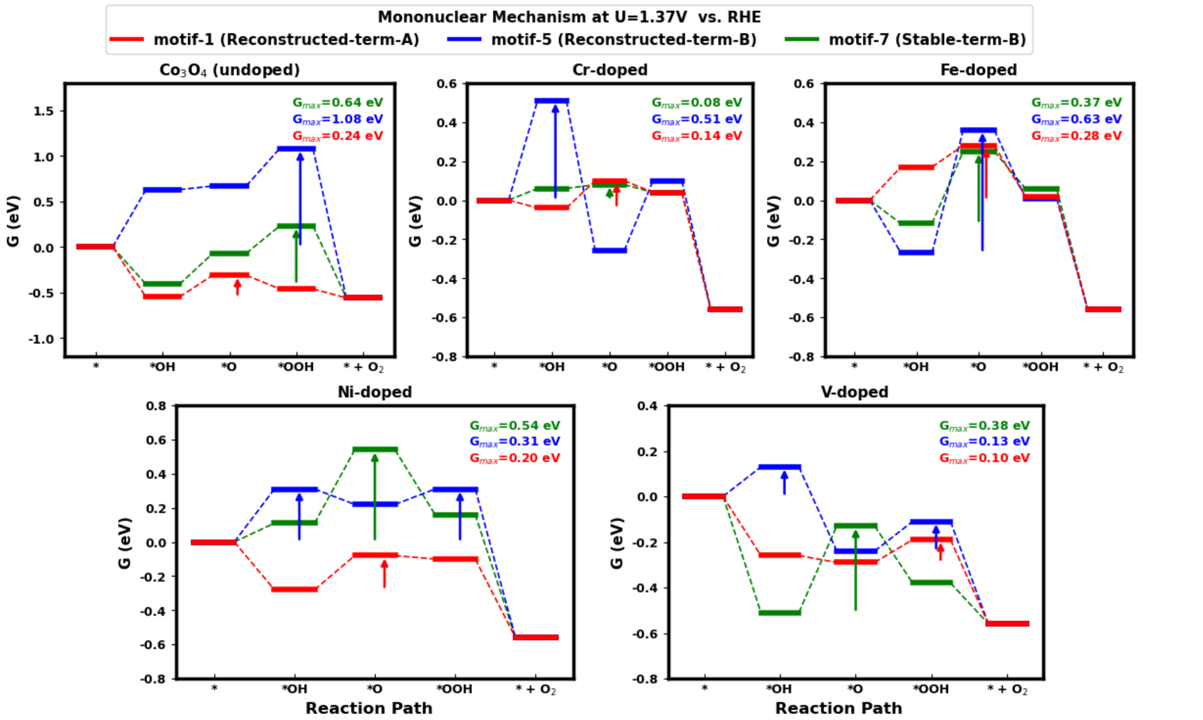

Figure S23. Mononuclear OER free-energy diagrams at U = 1.37 V vs. RHE for pristine and TM-doped Co_3O_4(001) evaluated on reconstructed surface motifs. The red, blue, and green curves correspond to motif-1 (reconstructed Termination-A), motif-5 (reconstructed Termination-B), and motif-7 (stable Termination-B), respectively. Results are shown for pure, Cr-, Fe-, Ni-, and V-doped systems. The calculated maximum free-energy span (G_{max}) is indicated in each panel. Differences in adsorption energetics arise from reconstruction and the consideration of a single reaction mechanism; a comparison of the resulting G_{max} values is provided in **Figure S24**.

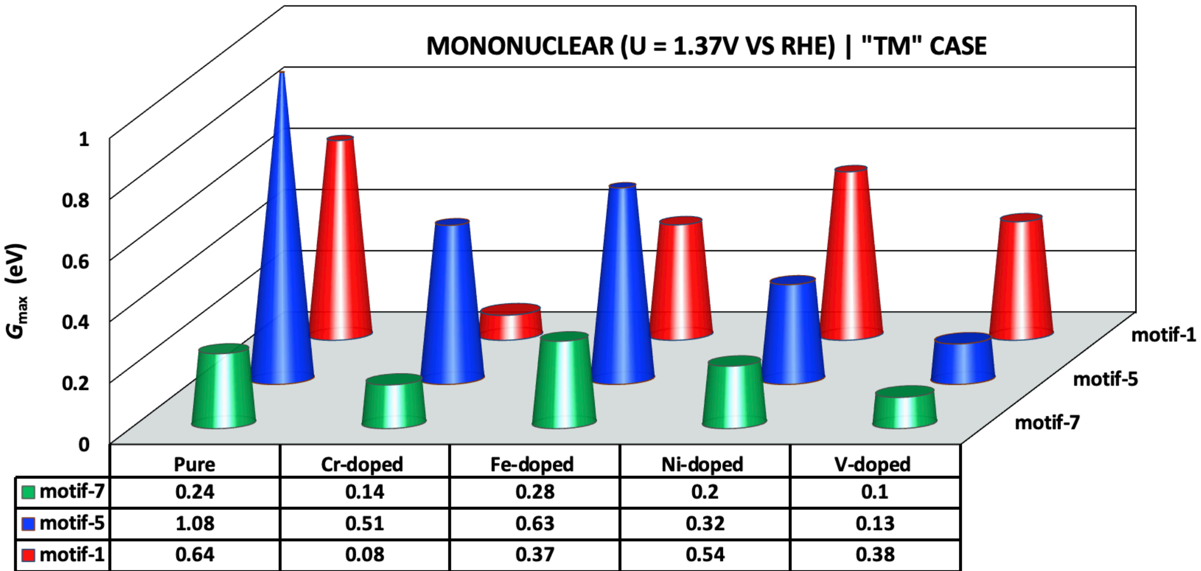

	Pure	Cr-doped	Fe-doped	Ni-doped	V-doped
motif-7	0.24	0.14	0.28	0.2	0.1
motif-5	1.08	0.51	0.63	0.32	0.13
motif-1	0.64	0.08	0.37	0.54	0.38

Figure S24. Comparison of the maximum free-energy span (G_{max}) for the mononuclear OER mechanism at U = 1.37 V vs. RHE on non-reconstructed and reconstructed surface motifs of Co_3O_4(001). Results are shown for pristine and TM-doped systems (Cr, Fe, Ni, and V) evaluated on motif-1, motif-5, and motif-7. While reconstructed motifs introduce moderate shifts in G_{max}, the overall activity ranking remains preserved, and Cr substitution consistently exhibits the lowest free-energy span (except motif-5) within the Co_3O_4 host lattice.

It is important to note that the reconstruction analysis presented here represents only a limited but representative subset of calculations. In particular, the reconstructed motifs were evaluated only for the mononuclear mechanism rather than for all seven OER mechanisms considered in the main study. Moreover, it is also possible that the detailed O/OH surface termination may vary depending on the dopant, which could further modify the local surface environment under operating conditions. Despite these additional complexities and the limited scope of the reconstruction calculations, the results consistently show that the relative activity enhancement induced by transition-metal doping remains unchanged. In other words, even when local reconstruction effects are explicitly considered, the doped Co_3O_4 systems still exhibit improved catalytic performance compared to pristine Co_3O_4. These results therefore strengthen our main conclusion that dopant incorporation in the Co_3O_4 host lattice enhances OER activity. On this basis, motif-7 was selected as the primary structural model for the detailed mechanistic analysis presented in the main manuscript. Differences in the relative activity trends among individual dopants are indeed present; however, resolving these differences rigorously would require evaluating all mechanistic pathways for each reconstructed motif, which lies beyond the scope of the present work.

In addition to theoretical investigations, a growing body of experimental work has demonstrated that cobalt oxide surfaces can dynamically evolve under electrochemical OER conditions. Operando and in situ studies on Co_3O_4 and related cobalt oxides have shown that exposure to aqueous electrolytes and anodic potentials can lead to structural and chemical changes in the near-surface region, often resulting in hydroxylated or oxyhydroxide-like surface layers during catalysis.[42–47] For example, operando surface X-ray diffraction and spectroscopy studies have revealed the formation of reversible reconstructed surface layers on Co_3O_4 under OER conditions while preserving the underlying spinel framework.[42] Other experimental investigations using advanced spectroscopic and electrochemical techniques have further highlighted the dynamic evolution of cobalt oxide surfaces toward oxyhydroxide-like states during operation.[43–46] More recent studies combining multimodal operando characterization also confirm that cobalt oxide catalysts can undergo surface restructuring depending on the applied potential and electrolyte environment.[47]

S8.5 Thicker hydroxide layer formation for the Co_3O_4(001) Model

Within the accessible time scales of previous AIMD simulations[31] and the reconstructed surface motifs explored previously[30,31] and here, no stable phase corresponding to a thicker

hydroxide overlayer has been identified for Co_3O_4 (001). Modeling such extended hydroxide layers would require describing a substantially different surface phase, potentially involving bulk-like oxyhydroxide formation. While such transformations may occur under strongly oxidizing conditions, their investigation would require dedicated phase-stability and dynamical simulations beyond the scope of the present electronic-structure framework. Even if thicker hydroxide layers were to form experimentally, they have not been identified within the AIMD simulations or reconstructed motifs examined here.

This interpretation is consistent with experimental observations by Magnussen and coworkers,[42] who reported the formation of a hydroxylated "skin layer" on cobalt oxide surfaces under OER conditions. Using synchrotron-based techniques, they showed that this layer remains confined to a thin near-surface region rather than forming a thick bulk-like hydroxide phase. This finding supports the present modeling approach, where the Co_3O_4(001) surface is described by O/OH-terminated motifs, while extended hydroxide overlayers are not explicitly considered.

S8.6 Effect of Surface Oxygen Vacancies

Under electrochemical OER conditions, oxygen vacancies may form on oxide surfaces and influence catalytic behavior. To assess the possible impact of such defects, we introduced a surface oxygen vacancy in the reconstructed Cr-doped Co_3O_4(001)-4*OH model and recalculated the mononuclear OER pathway.

The structural response to vacancy formation is shown in **Figure S25**. Upon creating a surface O vacancy, significant local reconstruction occurs. Surface OH groups tend to migrate toward the vacancy site, and the surrounding coordination environment rearranges to partially compensate for the missing oxygen. This behavior reflects the intrinsically dynamic nature of the reconstructed Co_3O_4(001) surface under oxidizing conditions. Importantly, vacancy formation does not simply create an isolated defect; instead, it induces collective surface relaxation and redistribution of adsorbates.

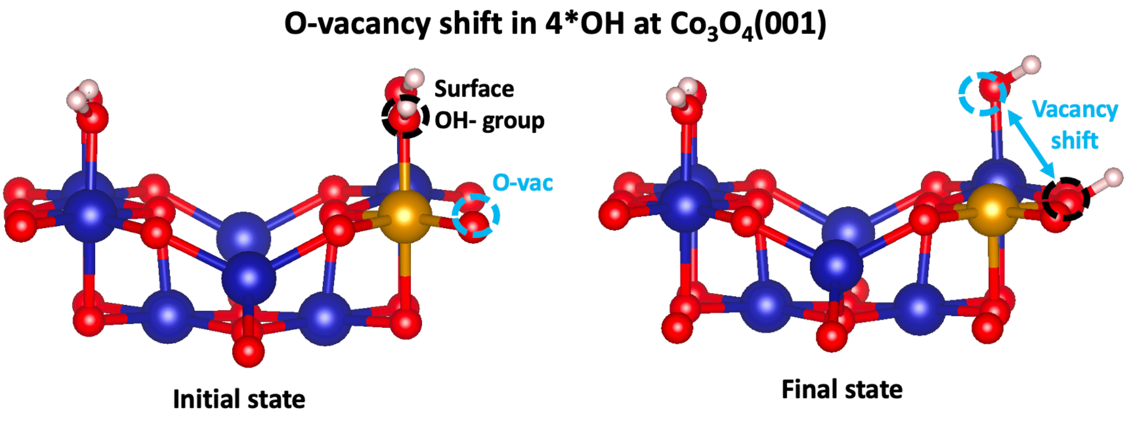

Figure S25. Initial and final structures illustrating oxygen-vacancy formation and vacancy migration on the reconstructed Co_3O_4(001)-4*OH surface in the Cr-doped system. An oxygen vacancy (O-vac) is introduced at the surface octahedral site, followed by structural relaxation showing vacancy-induced rearrangement of neighboring surface OH groups. The images highlight the strong local reconstruction and OH migration toward the vacancy site under OER-relevant conditions.

The corresponding free-energy diagrams are presented in **Figure S26**. In the absence of vacancy (left panel), the Cr-doped system exhibits a low G_{max} in the TM case, consistent with the promotion effect discussed in the main manuscript. Upon introducing an oxygen vacancy (right panel), the adsorption energetics are modified and the relative stability of intermediates changes. In particular, some reaction steps show pronounced shifts in free energy, leading to changes in G_{max} for the Co-, TM-, and 2TM-cases. These variations arise from vacancy-induced changes in local electronic structure and coordination, as well as from OH migration and surface reorganization.

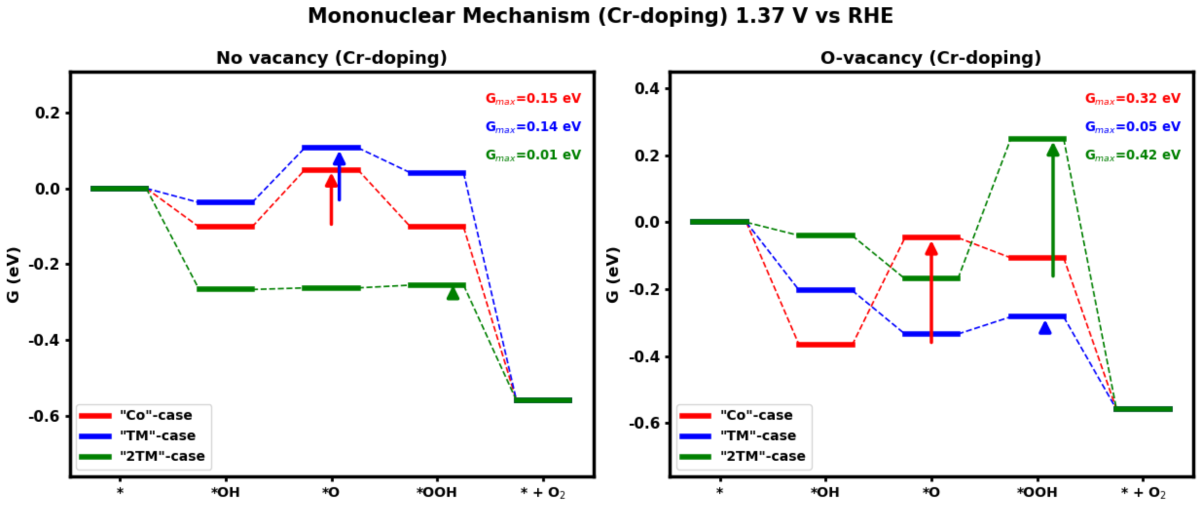

Figure S26. Free-energy diagrams of the mononuclear OER mechanism at 1.37 V vs RHE for Cr-doped Co_3O_4(001) without oxygen vacancy (left) and with a surface oxygen vacancy (right). Results are shown for the Co-case, TM-case, and 2TM-case. The presence of an oxygen vacancy modifies adsorption energetics and alters the maximum free-energy span G_{max}, reflecting vacancy-induced changes in surface structure and reaction energetics.

However, we note that the vacancy-containing surface does not remain static along the reaction pathway. The reconstructed Co_3O_4(001) surface already exhibits *O/*OH mobility, and the introduction of a vacancy enhances this effect. As a result, G_{max} may exhibit non-monotonic changes due to structural rearrangements between intermediates. A rigorous treatment of vacancy formation energies, vacancy concentrations under electrochemical potential, and dynamic surface restructuring would require a more extensive study involving thermodynamic defect analysis and possibly ab initio molecular dynamics, which is beyond the scope of the present work.

S9 Electrochemical Kinetic Analysis and Theoretical Interpretation

S9.1 Tafel analysis and electrochemical impedance spectroscopy of pulsed optimized samples

Previous studies have reported that cobalt-based oxides, including doped Co_3O_4, undergo surface reconstruction into cobalt (oxy)hydroxide species during the oxygen evolution reaction (OER), with the resulting activity strongly depending on the initial catalyst structure and composition.[48] In general, the presence of dopants has been shown to enhance OER activity.[33,49]

Operando studies have revealed that surface reconstruction towards an amorphous yet catalytically active cobalt-(oxy)hydroxide (CoOOH) layer occurs at potentials around 1.37 V, driven by a dynamic change from Co^{2+} to Co^{3+}.[50] This is consistent with the loss of activity observed when Co^{2+} is replaced by redox-inactive Zn^{2+}, which suppresses this oxidation dynamics and highlights the crucial role of the redox flexibility at the tetrahedral site.[51]

In our PUDEL-treated doping series, significant lower value of charge transfer resistance (R_{ct}) for Fe- and V-doped Co_3O_4 after laser treatment indicate enhanced kinetics, whereas Cr and Ni incorporation slightly increment it. Faster charge transfer supports the surface redox cycle and thus promotes catalytic activity via an increased TOF, while slower kinetics dampen this effect.[51,52]

Overall, the experimental doping series confirms the promotional effect of dopants on Co_3O_4 in OER, mainly through modulation of the cobalt redox cycle, in agreement with operando studies.[50,51] At the same time, a direct participation of dopants such as Fe and V in the surface reaction cannot be fully excluded, so that overlapping promotion mechanisms (improved charge-transfer kinetics and possible additional active sites) are likely. These correlations

demonstrate that, even for a dynamically reconstructing surface under OER-relevant redox conditions, the theoretical predictions are sufficiently reliable to identify suitable promoters.

To further evaluate the catalytic performance of pristine and transition-metal-doped Co_3O_4 catalysts, additional electrochemical analyses were performed beyond LSV measurements presented in the main manuscript. In particular, Tafel slope analysis and electrochemical impedance spectroscopy (EIS) were conducted to gain further insight into the reaction kinetics and charge-transfer properties of the investigated catalysts.

The corresponding Tafel plots for the pulsed optimized samples are shown in **Figure S27a**, and the extracted Tafel slopes together with the overpotentials required to reach a current density of 10 mA cm^{-2} are summarized in **Table S5**. The experimentally determined Tafel slopes measured in the OER overpotential region of approximately 400 mV fall within a narrow range of 60-67 mV/dec for pristine and doped Co_3O_4. This indicates that the intrinsic reaction pathways remain largely similar for all investigated catalysts under the measured conditions.

Electrochemical impedance spectroscopy (EIS) measurements were further carried out under OER conditions to investigate the interfacial charge-transfer processes. The corresponding Nyquist plots are presented in **Figure S27b**, and the extracted fitting parameters obtained from the equivalent circuit model are summarized in **Table S6**. The results reveal that the doped systems generally exhibit lower charge-transfer resistance (R_{ct}) compared with pristine Co_3O_4, indicating improved interfacial electron-transfer kinetics during the oxygen evolution reaction.

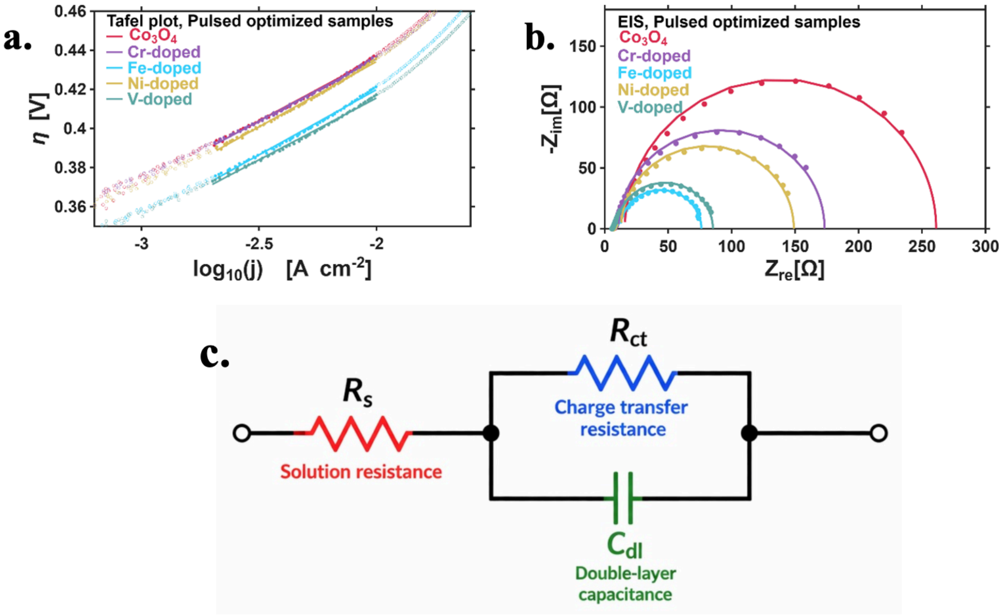

Figure S27 (a) Tafel analysis of doped Co_3O_4 for OER. Dopants were introduced by laser pulse treatment: 3 pulses per volume (PPV) for Cr, Ni, and V, and 2 PPV for Fe., The scan speed of the LSV is 5 mV/s, and the

potential window for the linear fit is 1.059 – 1.4759 V vs RHE. (b) Electrochemical impedance spectroscopy (EIS) in OER conditions of doped Co_3O_4, the potential used for the measurements is 1.5 V vs RHE. Electrocatalytic measurements were performed in 1 M KOH with a rotating disk electrode (RDE), Pt/C as the counter electrode, and Ag/AgCl (3 M KCl) as the reference electrode. (c) equivalent circuit model used for the fitting of the EIS data where the R_{ct}, the solution resistance (R_s) the double layer capacitance (C_{dl}) and the Warburg impedance (Z_W) are taken into account.

Electrocatalytic measurements were performed in 1 M KOH using a rotating disk electrode (RDE) configuration. Pt/C served as the counter electrode and Ag/AgCl (3 M KCl) as the reference electrode. Linear sweep voltammetry was conducted at a scan rate of 5 mV/s, and the Tafel slopes were extracted between 370 mV and 410 mV of OER overpotential. Dopants were introduced by laser pulse treatment using 3 pulses per volume (PPV) for Cr, Ni, and V, and 2 PPV for Fe.

Table S5: Tafel slopes obtained from linear fitting of the Tafel plots for pristine and transition-metal-doped Co_3O_4 catalysts. The corresponding overpotentials required to reach a current density of 10 mA cm^{-2} are also listed. All values are reported as mean ± fitting error.

Sample	Tafel Slope [mV/dec]	Overpotential @10 mA cm^{-2} [mV]
**Co_3O_4**	64.6 ± 0.4	443.1 ± 0.2
**Cr-doped**	63.7 ± 0.4	443.0 ± 0.3
**Fe-doped**	66.8 ± 0.5	431.6 ± 0.3
**Ni-doped**	66.8 ± 0.4	442.4 ± 0.3
**V-doped**	63.2 ± 0.5	427.9 ± 0.2

Table S6. Electrochemical impedance spectroscopy (EIS) fitting parameters for pristine and transition-metal-doped Co_3O_4 catalysts obtained from Nyquist plot analysis. The solution resistance (R_s) and charge-transfer resistance (R_{ct}) were extracted using an equivalent circuit model. All values are reported as mean ± fitting error. Lower R_{ct}values indicate improved interfacial charge-transfer kinetics during the OER process for the doped systems compared with pristine Co_3O_4.

Sample	R_s [Ω]	R_{ct} [Ω]
**Co_3O_4**	16 ± 2	245 ± 49
**Cr-doped**	11 ± 1	162 ± 32
**Fe-doped**	12 ± 1	64 ± 13
**Ni-doped**	13 ± 2	136 ± 27
**V-doped**	9 ± 1	76 ± 15

S9.2 Surface-enriched Cr-doped Co_3O_4: kinetic and charge-transfer comparison

Tafel slopes and charge transfer resistances (obtained from EIS at OER potential) were also compared for the undoped Co_3O_4 and Cr-doped Co_3O_4. Remarkably, both Cr-doped Co_3O_4 samples exhibit significantly smaller Tafel slope and charge-transfer resistance (R_{ct}) than the undoped Co_3O_4, as summarized in **Table S7**. This behavior indicates enhanced intrinsic surface kinetics and improved charge-transfer properties for the Cr-enriched catalyst. For pristine

Co_3O_4, the experimental slope (54 mV/dec) is consistent with our calculated value (~58–60 mV/dec). With Cr substitution, the experimental Tafel slope (~33 mV/dec) is in excellent agreement with the simulated Cr(TM) model where the initial regime at low overpotentials shows ~30–35 mV/dec (*cf.* **Figure S30**). The reduced Tafel slope with Cr incorporation suggests a shift in the rate-determining kinetics. These findings therefore confirm that surface or even bulk enrichment with Cr dopants is beneficial for both the reaction kinetics at the catalytic surface and the overall charge-transfer processes.

Table S7: Tafel slope and charge transfer resistance (obtained from EIS data at OER potential) undoped Co_3O_4 and Cr-doped Co_3O_4.

Catalyst	Tafel slope (mV/dec)	*R_{ct} (Ω)
Co_3O_4	54	539
$Cr_{0.1}Co_{2.9}O_4$	33	9
$Cr_{0.1}Co_{2.9}O_4$-calcined	46	94

*R_{ct} represents the charge transfer resistance of the OER process. Data is extracted from the graphs deposited in our ChemRxiv preprint.[40].

S9.3 Relation between experimental kinetics and the theoretical descriptor $G_{\mathbf{max}}^{\mathbf{min}}(U)$

To further interpret these experimental observations, we link the measured catalytic trends for the surface-enriched Cr-doped samples to the theoretical activity descriptor $G_{\text{max}}(U)$obtained from our DFT analysis.

Below, we clarify from the theoretical side how the descriptor $G_{max}^{min}(U)$ is directly connected to current density, overpotential, and Tafel slope, and how this framework can be compared consistently with the new experimental data.

In our theoretical approach, $G_{\text{max}}(U)$ is defined as the potential-dependent maximum free-energy rise among all elementary steps of a given OER pathway at an applied potential U. For each mechanism, we compute the complete free-energy profile and identify the largest uphill step at that potential. The kinetically relevant transition state is then defined as the minimum thermodynamic free-energy span over all considered mechanisms, following previous work in the literature.[30,53]

$$G_{max}^{min}(U) = \min_{mechanisms} G_{max}(U) \quad \text{(S58)}$$

$G_{max}^{min}(U)$ serves as a measure to approximate the OER kinetics and can be directly linked to macroscopic quantities, such as the current density, *j*(U). This is achieved by combining transition-state theory and Faraday's law,[54] as shown in Eq. (S59),

$$j(U) \propto \exp\left[-\frac{G_{max}^{min}(U)}{K_B T}\right] \tag{S59}$$

The Tafel slope, *b*, is defined as

$$b = \frac{dU}{d\log_{10} j} \tag{S60}$$

Combining Eq. (S60) with the exponential dependence of j on $G_{max}^{min}(U)$ gives a direct connection between the Tafel slope and how the dominant free-energy span changes with applied potential:

$$b \approx \frac{2.303\, K_B T}{e}\left(\frac{dG_{max}^{min}(U)}{dU}\right)^{-1} \tag{S61}$$

Therefore, changes in $G_{max}^{min}(U)$ (magnitude and mechanism switches that alter its (*U*)-dependence) translate into changes in the apparent Tafel slope. Importantly, we do not argue that the above approach allows us to determine Tafel slopes that can be quantitatively compared to experimental data, although changes in the as-determined Tafel slopes with increasing overpotential can be qualitatively linked to experimental trends. Note that the reported approach provides Tafel slopes of 30 mV/dec and 60 mV/dec, as the discussion of $G_{max}^{min}(U)$ as activity descriptor does not include the transfer coefficient in the analysis. Therefore, our particular interest lies in comparing surface models that do not reveal a change in the Tafel slope (unfavorable intrinsic kinetics) and surface models with a change in the Tafel slope from 30 mV/dec to 60 mV/dec (favorable intrinsic kinetics).

The theoretical results are summarized in **Figures S28–S30**. **Figure S28** shows the multi-mechanism $G_{\mathrm{max}}(U)$ curves for pristine Co_3O_4 and for the Cr-doped configurations (TM, Co, and 2TM cases). These plots demonstrate how different mechanistic pathways compete as a function of potential and where crossovers occur. **Figure S29** presents the corresponding $G_{max}^{min}(U)$ envelopes, which represent the lowest accessible barrier at each potential and therefore determine the governing kinetics. Compared to pristine Co_3O_4, the Cr-containing systems exhibit a systematic downward shift of $G_{max}^{min}(U)$ across the relevant OER potential window. **Figure S30** converts $G_{max}^{min}(U)$ into Tafel-type plots. The slopes extracted from these plots reflect the potential dependence of the dominant barrier and change when the governing mechanism changes or when another free-energy span becomes limiting in the picture of $G_{max}^{min}(U)$.

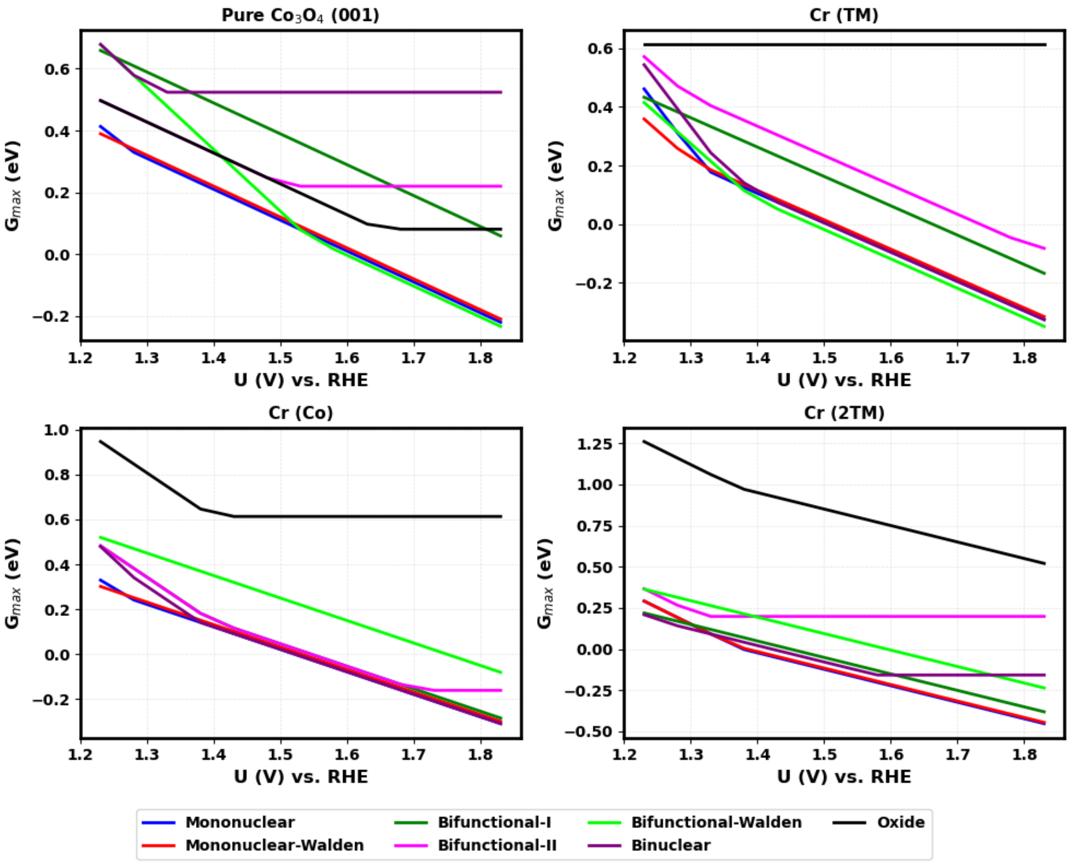

Figure S28. Potential-dependent maximum free-energy barriers $G_{\max}(U)$ for the oxygen evolution reaction (OER) on Co_3O_4(001) and Cr-substituted surfaces. The pristine surface and three substitution motifs (Cr(TM), Cr(Co), and Cr(2TM)) are compared across multiple pathways. At each potential, the lowest barrier identifies the kinetically preferred pathway. Cr substitution alters mechanistic competition and stabilizes alternative O-O bond formation routes, leading to reduced barriers relative to the pristine surface.

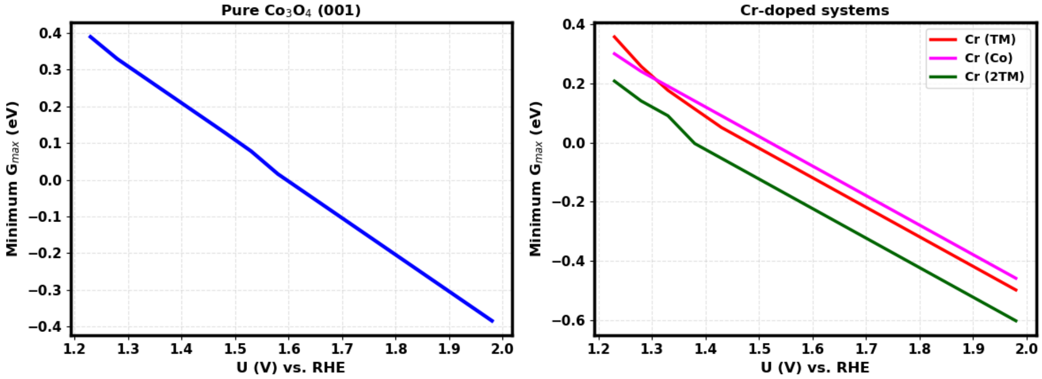

Figure S29. Minimum free-energy barrier $G_{\min}(U)$, obtained as the envelope of all evaluated mechanisms. Left: pristine Co_3O_4(001). Right: Cr-substituted systems (Cr(TM), Cr(Co), and Cr(2TM)). The systematic downward shift of $G_{\min}(U)$ upon Cr substitution indicates a reduced kinetic bottleneck and predicts enhanced catalytic activity. The Cr(2TM) configuration exhibits the lowest barriers over the relevant potential range.

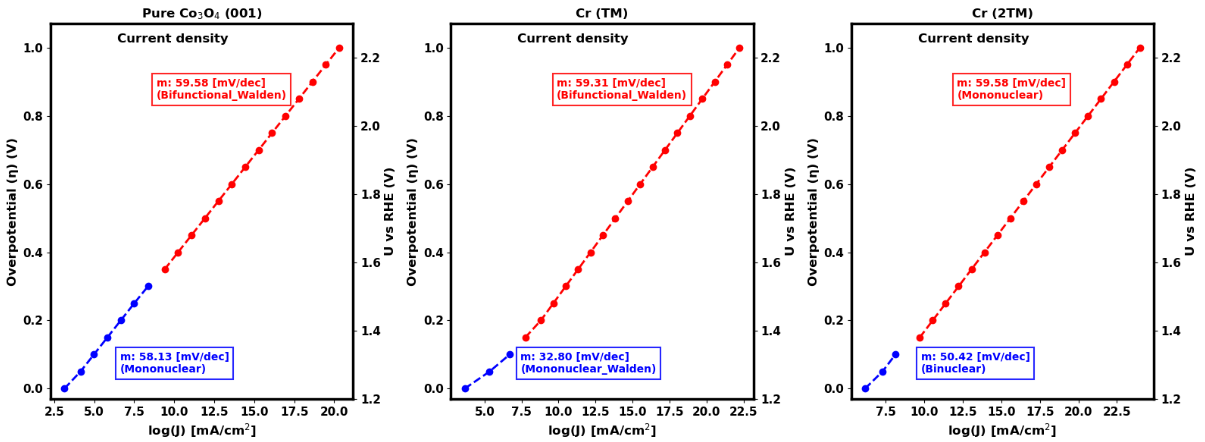

Figure S30. Theoretically derived current density (Tafel-type) plots obtained by mapping the minimum free-energy span $G_{max}^{min}(U)$onto current density using the exponential barrier–rate relationship described in the text. Panels compare pristine Co_3O_4 and Cr-substituted cases (TM and 2TM configurations). The slopes are extracted from the linear regions and represent the apparent Tafel slopes arising from the potential dependence of the dominant kinetic barrier. Differences in slope reflect changes in the governing mechanism and barrier response to applied potential rather than changes in the total number of electrons involved in the OER.

Regarding the comparison with experiment, we note that for pristine Co_3O_4 the experimentally measured Tafel slope (~54 mV/dec, **Table S7**) is consistent with the theoretical value of ~58–60 mV/dec derived from the potential dependence of $G_{max}^{min}(U)$ (**Figure S30**). Importantly, the theoretical analysis shows that this slope remains essentially unchanged for the undoped surface over the relevant potential range. In contrast, for the Cr-substituted systems the calculations predict a switch in the apparent Tafel slope, where the initial kinetic regime yields a smaller theoretical slope of approximately 30-35 mV/dec for the Cr(TM) configuration. This predicted change in slope is in direct agreement with the experimental measurements, where the surface-enriched Cr-doped Co_3O_4 exhibits a smaller Tafel slope (~33 mV/dec) compared to undoped Co_3O_4 (~54 mV/dec) (**Table S7**). The theoretical results therefore reproduce the experimentally observed trend that Cr incorporation modifies the kinetic response of the catalyst, leading to a reduced Tafel slope and enhanced catalytic activity relative to pristine Co_3O_4.

S10 Hubbard *U* Determination and Its Impact on Catalytic Energetics

Two distinct quantities denoted by "U" appear in the present work: (i) the Hubbard U parameter used in the DFT+U calculations and (ii) the applied electrode potential U entering the computational hydrogen electrode (CHE) formalism.

First, concerning the Hubbard U parameter, the on-site interaction values were determined using the linear-response (LR) approach of Cococcioni and de Gironcoli.[5] In this method, a small on-site potential perturbation α is applied to the d orbitals of a selected metal center and the response of the projected d-electron occupation is monitored. From the slopes of the occupation response obtained in self-consistent (SCF) and non-self-consistent (NSCF) calculations, the response functions are defined as

$$\chi = \frac{dn_d^{SCF}}{d\alpha} \tag{S62}$$

$$\chi_0 = \frac{dn_d^{NSCF}}{d\alpha} \tag{S63}$$

and the effective on-site interaction is obtained as:

$$U = \chi_0^{-1} - \chi^{-1} \tag{S64}$$

Following VASP implementation guidelines, perturbations in the range ± 0.20eV were applied and linear fits were performed around $\alpha = 0$ eV. The corresponding LR response curves and fitted slopes for Co and all dopants are presented in **Figure S31**. The LR-derived values adopted in this work are summarized in **Table S8** (Co 3.0 eV, Cr 3.7 eV, Mn 3.5 eV, Fe 4.5 eV, Ni 4.2 eV, Cu 7.0 eV, V 1.0 eV). These values fall within the physically expected range for 3d transition-metal oxides and reproduce reasonable magnetic and electronic trends.

Because the central objective of this work is to compare dopant- and motif-dependent catalytic trends within a large, internally consistent dataset, maintaining a uniform correlation treatment across all systems is essential. To explicitly address the sensitivity of the catalytic conclusions to the chosen Hubbard U, we performed a systematic U-variation test. The corresponding free-energy diagrams are shown in **Figure S32**, and the extracted G_{max} values are summarized in **Figure S33** and **Table S8**.

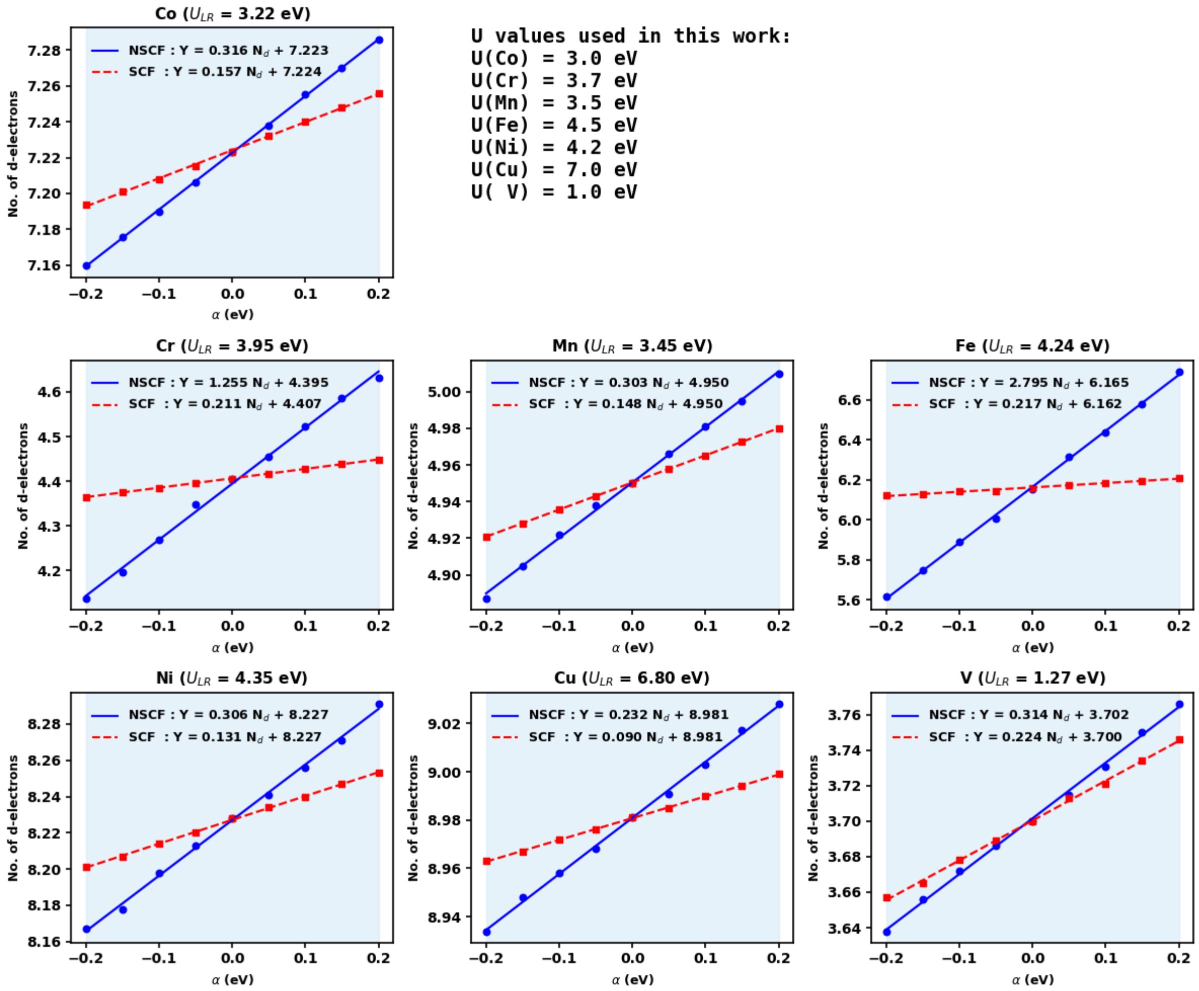

Figure S31. Occupation response of the metal d-electron population as a function of applied on-site perturbation α for Co and the substituted transition metals. Slopes obtained from self-consistent (SCF) and non-self-consistent (NSCF) calculations yield the response functions χ and χ_0, from which the effective on-site interaction is calculated according to $U = \chi_0^{-1} - \chi^{-1}$(Eqs. (S62)–(S64)). Linear fits were performed near $\alpha = 0$ eV within the perturbation range ±0.20 eV.

Table S8. Hubbard U parameters determined from the linear-response (LR) method **(Eqs. (S62)–(S64))** for Co and the substituted 3d transition metals. For each system, additional low and high U values around the LR-derived reference were tested to evaluate the sensitivity of the mononuclear OER mechanism (TM case) at 1.37 V vs RHE. The corresponding maximum free-energy span $G_{\max}$ values demonstrate that the relative catalytic trends remain robust across the physically reasonable U window.

	***U* (low)**		***U* (mid)**		***U* (high)**	
**System**	***U* (eV)**	**$G_{\max}$ (eV)**	***U* (eV)**	**$G_{\max}$ (eV)**	***U* (eV)**	**$G_{\max}$ (eV)**
Co_3O_4(pure)	2.0	0.24	3.0	0.24	4.0	0.29
Cr-doped	2.7	0.06	3.7	0.14	4.7	0.21
Mn-doped	2.5	0.49	3.5	0.53	4.5	0.59
Fe-doped	3.5	0.27	4.5	0.28	5.5	0.29
Ni-doped	3.2	0.23	4.2	0.20	5.2	0.15
Cu-doped	6.0	0.43	7.0	0.37	8.0	0.36
V-doped	1.0	0.17	2.0	0.10	3.0	0.13

Across this physically reasonable range of Hubbard parameters, the calculated free-energy span $G_{\max}$ varies only moderately. For most systems the variation is below ~0.10 eV, indicating a

weak sensitivity of the predicted catalytic energetics to the chosen U value. The largest variation is observed for the Cr-doped system, where G_{max} changes by approximately 0.15 eV across the tested U window. Importantly, despite these small quantitative variations, the qualitative activity ranking and mechanistic conclusions remain unchanged. In particular, the enhanced catalytic performance predicted for the Cr-doped configuration persists across the entire U range. These results demonstrate that the central catalytic trends reported in this work are robust with respect to moderate variations of the Hubbard parameter.

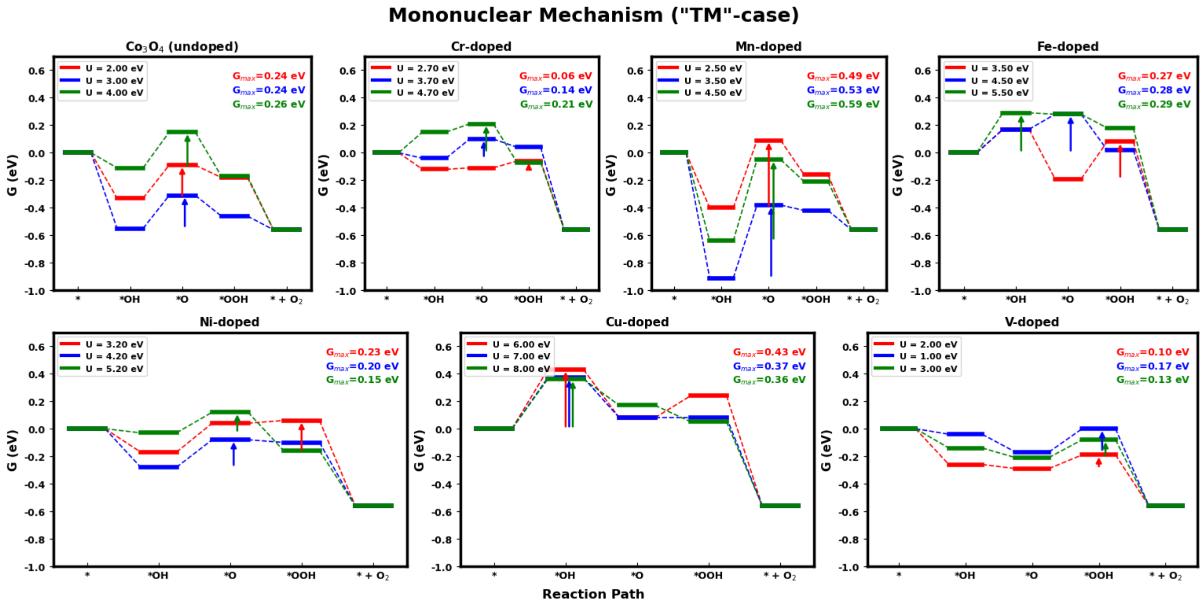

Figure S32. Calculated OER free-energy profiles at 1.37 V vs RHE for pristine and TM-doped $Co_3O_4(001)$ using low, reference (LR-derived), and high Hubbard U values for each metal center. The comparison illustrates how moderate variations of U affect adsorption energetics while the qualitative activity ranking and mechanistic trends remain largely unaffected.

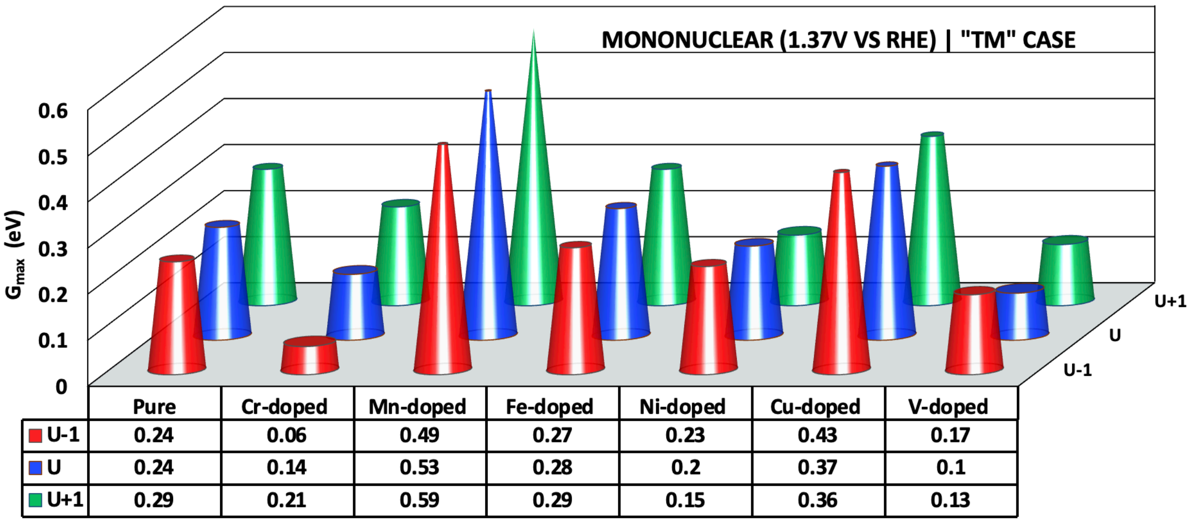

	Pure	Cr-doped	Mn-doped	Fe-doped	Ni-doped	Cu-doped	V-doped
U-1	0.24	0.06	0.49	0.27	0.23	0.43	0.17
U	0.24	0.14	0.53	0.28	0.2	0.37	0.1
U+1	0.29	0.21	0.59	0.29	0.15	0.36	0.13

Figure S33. Maximum free-energy span G_{max}(1.37 V vs RHE) extracted from **Figure S29** for different U values around the LR-derived reference. The limited variation in G_{max} across the tested U window demonstrates the robustness of the descriptor-based screening and confirms that the relative activity trends are not sensitive to moderate changes in the Hubbard parameter.

To further assess the consistency of the electronic-structure description, we also analyze the dependence of the calculated bulk electronic structure of Co_3O_4 on the chosen Hubbard parameter U_{Co}. The corresponding total and projected density-of-states calculations for U_{Co}=2, 3, and 4 eV are shown in **Figure S34**.

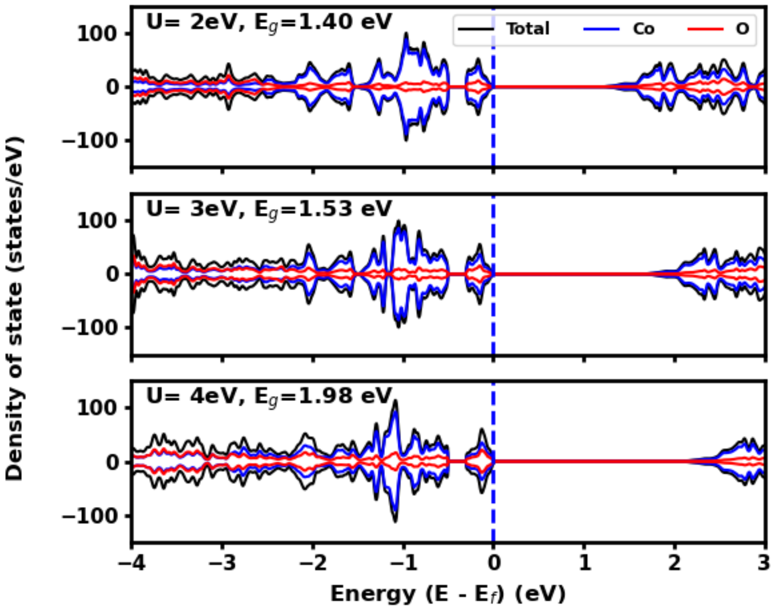

Figure S34. Calculated electronic band gap of bulk Co_3O_4 as a function of U_{Co}: 2 eV, 3 eV, and 4 eV. The band gap increases with increasing U, reflecting enhanced localization of Co 3d states. This analysis confirms that the selected U range yields physically reasonable electronic structure trends while not altering the relative catalytic conclusions.

As expected, the band gap increases with increasing U_{Co}, reflecting the enhanced localization of Co 3d states. Importantly, while the absolute band gap varies with the choice of U, the qualitative electronic-structure characteristics remain unchanged. Consequently, the relative adsorption energetics and mechanistic trends that underpin the descriptor-based catalytic analysis are not affected by the selected U value.

Finally, we clarify that the applied electrode potential U in the CHE formalism is conceptually distinct from the Hubbard U. In the CHE framework, the potential explicitly modifies the free energy of proton–electron transfer steps according to

$$\Delta G(U) = \Delta G(0) - neU \qquad \text{(S65)}$$

where n is the number of transferred electrons and e refers to the elementary charge. Consequently, shifting the applied potential changes the relative stability of intermediates, which can alter the free-energy span of $G_{\max}(U)$ and thus induce crossovers between competing mechanistic pathways. This potential-dependent mechanistic competition is

explicitly captured in our $G_{\max}(U)$-based analysis and constitutes a central element of the volcano-type screening presented in this work (**Figure 5** in the manuscript).

S11 Electronic Structure Modulation by Dopant Substitution

To rationalize the catalytic promotion observed upon transition-metal substitution, we performed an explicit electronic-structure analysis beyond the energetic descriptors discussed in the main manuscript. In particular, total and projected density-of-states (DOS) calculations were carried out for both bulk Co_3O_4 and the OER-relevant Co_3O_4(001)-4*OH surface models in the TM substitution configuration. The results are summarized in **Figure S35**. These calculations provide insight into how dopant incorporation modifies the electronic structure of the host lattice and thereby establishes the electronic basis for the catalytic promotion discussed in the manuscript.

In the present work, the promotion effect is defined mechanistically through three local configurations: (i) the TM case, where the dopant itself acts as the active site; (ii) the Co case, where the dopant modifies the electronic environment of a neighboring Co site; and (iii) the 2TM case, which represents cooperative interactions between two dopant centers. The DOS analysis presented in **Figure S35** focuses on the TM configuration, which captures the primary electronic perturbation introduced by substitution. Once this fundamental perturbation of the host lattice is understood, the electronic influence on neighboring Co sites and cooperative configurations can be interpreted as an extension of the same local electronic modulation.

The calculated bulk projected DOS (**Figure S35**, left column) for pristine Co_3O_4 exhibits the expected semiconducting electronic structure with a clear band gap around the Fermi level. Upon transition-metal substitution, additional dopant-derived d-states appear near the band edges and, in some cases, partially populate the region close to the Fermi level. These features reflect changes in the metal-oxygen hybridization and modifications of the electronic density near E_f. Such dopant-induced electronic perturbations influence the charge distribution and screening within the lattice and therefore represent the intrinsic electronic modification introduced by the substitution.

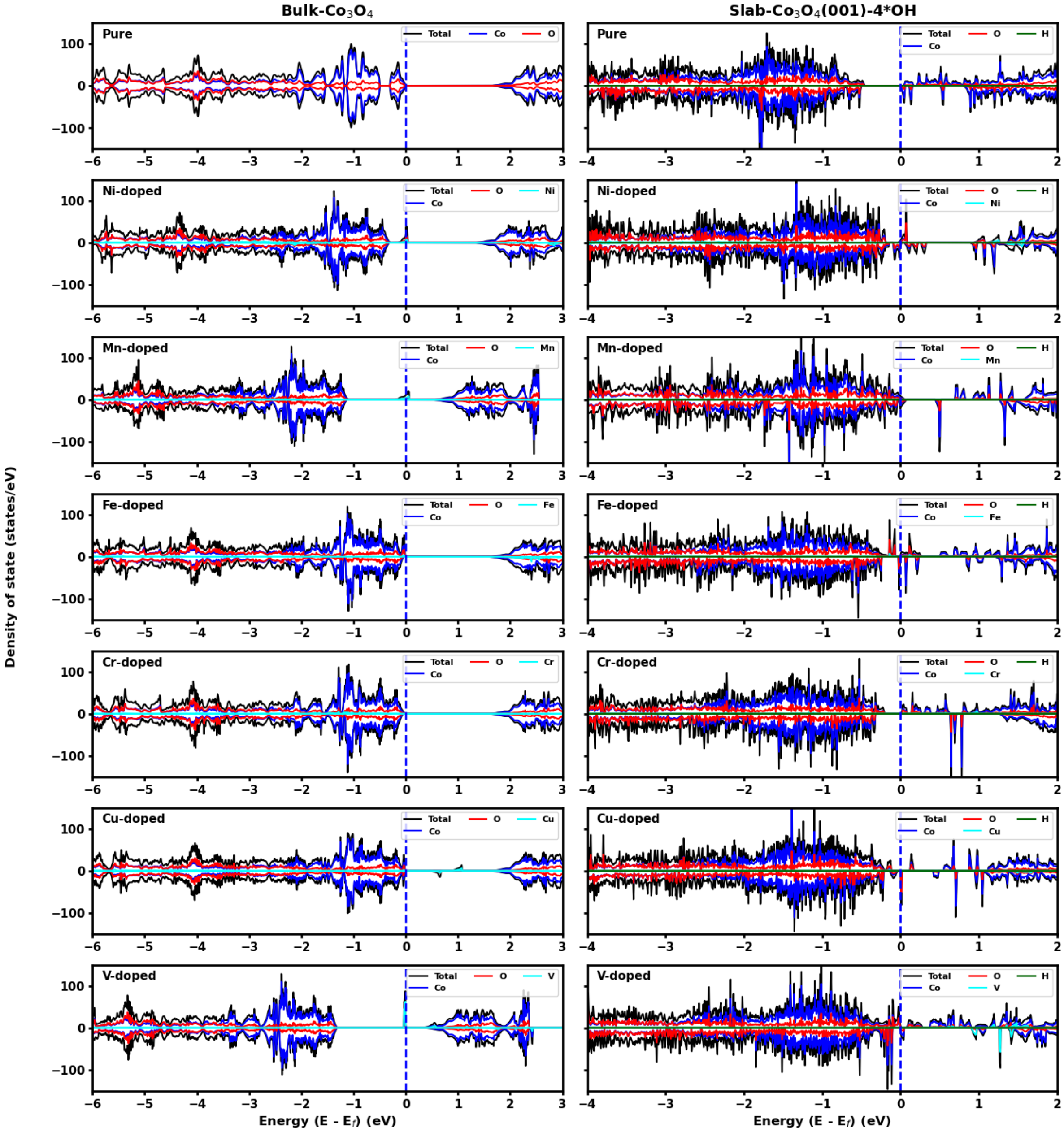

Figure S35. Total and projected density of states (DOS) for pristine and TM-doped Co_3O_4 systems. Left column: bulk Co_3O_4 for pure, Ni-, Mn-, Fe-, Cr-, Cu-, and V-doped cases. Right column: OER-relevant Co_3O_4(001)-4*OH slab models for the corresponding systems in the TM configuration. The total DOS is shown in black, Co-projected states in blue, O in red, H in green (slab only), and dopant-projected states in cyan. The dashed vertical line indicates the shifter Fermi level (at 0eV). The comparison highlights dopant-induced modifications of the electronic structure near the Fermi level in both bulk and surface environments

More directly relevant for catalysis is the electronic structure of the surface models shown in **Figure S35** (right column). The Co_3O_4(001)-4*OH slab represents the stable O/OH-covered surface under OER conditions. Compared to the bulk electronic structure, the surface exhibits a higher density of electronic states near the Fermi level, which arises from the reduced coordination of surface atoms and from interactions between surface sites and adsorbed

species. When a transition-metal dopant is introduced, the distribution of d-states near the Fermi level is modified, indicating changes in the local electronic structure at the catalytic site.

In particular, Cr substitution introduces additional electronic states in the vicinity of the Fermi level and alters the hybridization between metal d-states and oxygen p-states. Although the presence of electronic states near E_f alone does not directly determine catalytic activity, it reflects the availability of electronic states that can participate in interactions with adsorbates. The catalytic performance ultimately depends on a balance between bond formation and bond breaking along the reaction pathway, which is quantified through the adsorption energetics and reaction free-energy diagrams discussed in the work. The DOS analysis therefore provides qualitative electronic-structure insight that supports the energetic trends obtained from the free-energy calculations and clarifies how dopant incorporation modulates the local electronic environment of the catalytic site.

References

[1] G. Kresse, J. Furthmüller, *Comput. Mater. Sci.* **1996**, *6*, 15.

[2] G. Kresse, J. Furthmüller, *Phys. Rev. B* **1996**, *54*, 11169.

[3] G. Kresse, J. Hafner, *Phys. Rev. B* **1993**, *48*, 13115.

[4] M. Perdew, John P and Burke, Kieron and Ernzerhof, *Phys. Rev. Lett.* **1996**, *77*, 3865.

[5] S. Cococcioni, M.; de Gironcoli, *Phys. Rev. B: Condens. Matter Mater. Phys* **2005**, *71*, 035105.

[6] S. Selcuk, A. Selloni, *Journal of Physical Chemistry C* **2015**, *119*, 9973.

[7] M. Calatayud, C. Minot, *The Journal of Physical Chemistry C* **2007**, *111*, 6411.

[8] X. Guo, E. Tiferet, L. Qi, J. M. Solomon, A. Lanzirotti, M. Newville, M. H. Engelhard, R. K. Kukkadapu, D. Wu, E. S. Ilton, M. Asta, S. R. Sutton, H. Xu, A. Navrotsky, *Dalton Transactions* **2016**, *45*, 4622.

[9] X. Chen, M. Vörös, J. C. Garcia, T. T. Fister, D. B. Buchholz, J. Franklin, Y. Du, T. C. Droubay, Z. Feng, H. Iddir, L. A. Curtiss, M. J. Bedzyk, P. Fenter, *ACS Appl. Energy Mater.* **2018**, *1*, 2526.

[10] H. Jani, J. Linghu, S. Hooda, R. V. Chopdekar, C. Li, G. J. Omar, S. Prakash, Y. Du, P. Yang, A. Banas, K. Banas, S. Ghosh, S. Ojha, G. R. Umapathy, D. Kanjilal, A. Ariando, S. J. Pennycook, E. Arenholz, P. G. Radaelli, J. M. D. Coey, Y. P. Feng, T. Venkatesan, *Nat. Commun.* **2021**, *12*, 1668.

[11] A. K. Mishra, A. Roldan, N. H. de Leeuw, *The Journal of Physical Chemistry C* **2016**, *120*, 2198.

[12] K. Dhaka, M. C. Toroker, *The Journal of Physical Chemistry C* **2019**, *123*, 18895.

[13] S. Grimme, J. Antony, S. Ehrlich, H. Krieg, *J. Chem. Phys.* **2010**, *132*.

[14] G. Kresse, *Phys. Rev. B* **1999**, *59*, 1758.

[15] K. Mathew, R. Sundararaman, K. Letchworth-Weaver, T. A. Arias, R. G. Hennig, *J. Chem. Phys.* **2014**, *140*.

[16] J. K. Nørskov, J. Rossmeisl, A. Logadottir, L. Lindqvist, J. R. Kitchin, T. Bligaard, H. Jónsson, *Journal of Physical Chemistry B* **2004**, *108*, 17886.

[17] M. Usama, S. Razzaq, C. Hättig, S. N. Steinmann, Exner K. S., *Nature Communication* **2025**, 6137.

[18] H. N. Nong, L. J. Falling, A. Bergmann, M. Klingenhof, H. P. Tran, C. Spöri, R. Mom, J. Timoshenko, G. Zichittella, A. Knop-Gericke, S. Piccinin, J. Pérez-Ramírez, B. R. Cuenya, R. Schlögl, P. Strasser, D. Teschner, T. E. Jones, *Nature* **2020**, *587*, 408.

[19] Y.-H. Fang, Z.-P. Liu, *J. Am. Chem. Soc.* **2010**, *132*, 18214.

[20] Y. Ping, R. J. Nielsen, W. A. Goddard, *J. Am. Chem. Soc.* **2017**, *139*, 149.

[21] N. B. Halck, V. Petrykin, P. Krtil, J. Rossmeisl, *Phys. Chem. Chem. Phys.* **2014**, *16*, 13682.

[22] M. Busch, *Curr. Opin. Electrochem.* **2018**, *9*, 278.

[23] M. Busch, E. Ahlberg, I. Panas, *Physical Chemistry Chemical Physics* **2011**, *13*, 15069.

[24] T. Binninger, M.-L. Doublet, *Energy Environ. Sci.* **2022**, *15*, 2519.

[25] K. S. Exner, *Journal of Physics: Energy* **2023**, *5*, 014008.

[26] J. Rossmeisl, A. Logadottir, J. K. Nørskov, *Chem. Phys.* **2005**, *319*, 178.

[27] J. Rossmeisl, Z. W. Qu, H. Zhu, G. J. Kroes, J. K. Nørskov, *Journal of Electroanalytical Chemistry* **2007**, *607*, 83.

[28] K. S. Exner, *Advanced Science* **2023**, *10*.

[29] S. Yu, Z. Levell, Z. Jiang, X. Zhao, Y. Liu, *J. Am. Chem. Soc.* **2023**, *145*, 25352.

[30] K. Dhaka, K. S. Exner, *J. Catal.* **2025**, *443*, 115970.

[31] K. Dhaka, S. Kenmoe, A. Füngerlings, R. Pentcheva, K. Tschulik, K. S. Exner, *ChemCatChem* **2025**, *17*, e00992.

[32] Q. Qi, S. Zhu, Z. Liu, C. Zhang, J. Hu, *J. Colloid Interface Sci.* **2026**, *701*, 138669.

[33] Y. Peng, H. Hajiyani, R. Pentcheva, *ACS Catal.* **2021**, *11*, 5601.

[34] Z. Zhang, G. Tan, A. Kumar, H. Liu, X. Yang, W. Gao, L. Bai, H. Chang, Y. Kuang, Y. Li, X. Sun, *Molecular Catalysis* **2023**, *535*, 112852.

[35] D. Gorylewski, F. Zasada, G. Słowik, M. Lofek, G. Grzybek, K. Tyszczuk-Rotko, A. Kotarba, P. Stelmachowski, *ACS Catal.* **2025**, *15*, 4746.

[36] D. Schellenburg, T. Bihnam, C. Placke-Yan, G. Bendt, O. Prymak, T. Sato, D. Jennings, C. Leiva-Leroy, D. Zhang, M. Nachev, K. Dhaka, F. Nkou, U. Hagemann, M. Heidelmann, S. Kenmoe, K. S. Exner, B. Sures, M. Muhler, C. H. Liebscher, A. Schnegg, S. Schulz, S. Barcikowski, S. Reichenberger, *ChemCatChem* **2025**.

[37] E. Budiyanto, S. Zerebecki, C. Weidenthaler, T. Kox, S. Kenmoe, E. Spohr, S. DeBeer, O. Rüdiger, S. Reichenberger, S. Barcikowski, H. Tüysüz, *ACS Appl. Mater. Interfaces* **2021**, *13*, 51962.

[38] S. Zerebecki, K. Schott, S. Salamon, J. Landers, H. Wende, E. Budiyanto, H. Tüysüz, S. Barcikowski, S. Reichenberger, *The Journal of Physical Chemistry C* **2022**, *126*, 15144.

[39] E. Budiyanto, S. Zerebecki, C. Weidenthaler, T. Kox, S. Kenmoe, E. Spohr, S. DeBeer, O. Rüdiger, S. Reichenberger, S. Barcikowski, H. Tüysüz, *ACS Appl. Mater. Interfaces* **2021**, *13*, 51962.

[40] C. Placke-Yan, H. Amin, G. Bendt, U. Hagemann, S. Schulz, *Chemrxiv* **2025**, 10.26434/chemrxiv.

[41] T. Kox, E. Spohr, S. Kenmoe, *Front. Energy Res.* **2020**, *8*.

[42] T. Wiegmann, I. Pacheco, F. Reikowski, J. Stettner, C. Qiu, M. Bouvier, M. Bertram, F. Faisal, O. Brummel, J. Libuda, J. Drnec, P. Allongue, F. Maroun, O. M. Magnussen, *ACS Catal.* **2022**, *12*, 3256.

[43] H. Jiang, Q. He, Y. Zhang, L. Song, *Acc. Chem. Res.* **2018**, *51*, 2968.

[44] B. He, F. Bai, P. Jain, T. Li, *Small* **2025**, *21*.

[45] H. Komiya, K. Obata, O. Sekizawa, K. Nitta, K. Xu, M. Wada, K. Takanabe, *Angew. Chem. Int. Ed.* **2025**, *64*.

[46] M. G. Ahmed, Y. F. Tay, X. Chi, A. S. Razeen, Y. Fang, M. Zhang, A. Sng, S. Y. Chiam, A. Rusydi, L. H. Wong, *Angew. Chem. Int. Ed.* **2025**, *64*.

[47] F. Reikowski, F. Maroun, I. Pacheco, T. Wiegmann, P. Allongue, J. Stettner, O. M. Magnussen, *ACS Catal.* **2019**, *9*, 3811.

[48] R. Zhang, L. Pan, B. Guo, Z.-F. Huang, Z. Chen, L. Wang, X. Zhang, Z. Guo, W. Xu, K. P. Loh, J.-J. Zou, *J. Am. Chem. Soc.* **2023**, *145*, 2271.

[49] E. M. Davis, A. Bergmann, H. Kuhlenbeck, B. Roldan Cuenya, *J. Am. Chem. Soc.* **2024**, *146*, 13770.

[50] J. Halldin Stenlid, M. Görlin, O. Diaz-Morales, B. Davies, V. Grigorev, D. Degerman, A. Kalinko, M. Börner, M. Shipilin, M. Bauer, A. Gallo, F. Abild-Pedersen, M. Bajdich, A. Nilsson, S. Koroidov, *J. Am. Chem. Soc.* **2025**, *147*, 4120.

[51] H.-Y. Wang, S.-F. Hung, H.-Y. Chen, T.-S. Chan, H. M. Chen, B. Liu, *J. Am. Chem. Soc.* **2016**, *138*, 36.

[52] B. He, P. Hosseini, T. Priamushko, O. Trost, E. Budiyanto, C. Bondue, J. Schulwitz, A. Kostka, H. Tüysüz, M. Muhler, S. Cherevko, K. Tschulik, T. Li, *Nat. Commun.* **2025**, *16*, 9895.

[53] S. Razzaq, K. S. Exner, *ACS Catal.* **2023**, *13*, 1740.

[54] K. S. Exner, *ACS Catal.* **2020**, *10*, 12607.
